# Multiplexed promoter and gene editing in wheat using a virus‐based guide RNA delivery system

**DOI:** 10.1111/pbi.13910

**Published:** 2022-09-07

**Authors:** Wei Wang, Zitong Yu, Fei He, Guihua Bai, Harold N. Trick, Alina Akhunova, Eduard Akhunov

**Affiliations:** ^1^ Department of Plant Pathology Kansas State University Manhattan KS USA; ^2^ Wheat Genetic Resources Center Kansas State University Manhattan KS USA; ^3^ Hard Winter Wheat Genetics Research Unit USDA‐ARS Manhattan KS USA; ^4^ Integrated Genomic Facility Kansas State University Manhattan KS USA; ^5^ Present address: State Key Laboratory of Plant Cell and Chromosome Engineering Institute of Genetics and Developmental Biology, Chinese Academy of Sciences Beijing China

**Keywords:** wheat genome editing, barley stripe mosaic virus, promoter editing, multiplex editing

## Abstract

The low efficiency of genetic transformation and gene editing across diverse cultivars hinder the broad application of CRISPR technology for crop improvement. The development of virus‐based methods of CRISPR‐Cas system delivery into the plant cells holds great promise to overcome these limitations. Here, we perform direct inoculation of wheat leaves with the barley stripe mosaic virus (BSMV) transcripts to deliver guide RNAs (sgRNA) into the Cas9‐expressing wheat. We demonstrate that wheat inoculation with the pool of BSMV‐sgRNAs could be used to generate heritable precise deletions in the promoter region of a transcription factor and to perform multiplexed editing of agronomic genes. We transfer the high‐expressing locus of Cas9 into adapted spring and winter cultivars by marker‐assisted introgression and use of the BSMV‐sgRNAs to edit two agronomic genes. A strategy presented in our study could be applied to any adapted cultivar for creating new *cis*‐regulatory diversity or large‐scale editing of multiple genes in biological pathways or QTL regions, opening possibilities for the effective engineering of crop genomes, and accelerating gene discovery and trait improvement efforts.

## Introduction

Crop improvement using the CRISPR‐Cas‐based editing relies on understanding the function of genes involved in the regulation of biological processes affecting phenotypic variation. While major advances were made towards linking genes with major agronomic phenotypes in wheat and other crops, and genome sequences facilitated inter‐species extrapolation of functional information among related species, the mechanistic basis of most of the traits remains poorly characterized. The advances in gene mapping and large‐scale genomic analyses helped to identify a number of quantitative trait loci (QTL) and biological pathways associated with major traits (He *et al*., 2022). However, the number of candidate causal genes detected in these studies still remained beyond the technical capabilities of existing genomic screens available for gene function validation in major crop species. The ability of CRISPR technology to introduce targeted changes into genomes has been broadly utilized in model systems to develop high‐throughput functional screens greatly accelerating the characterization of causal genes and pathways (Shalem *et al*., 2015). Although, the large‐scale CRISPR‐Cas editing is a powerful gene discovery tool, the scope of its application in crop genetics is limited by the complexity and cost of such projects (Liu *et al*., 2020) and warrants the development of more effective gene editing strategies.

In many crops, including wheat, the realization of the full potential of CRISPR technology is hindered by a combination of methodological challenges. While CRISPR technologies based on the Cas9 and Cas12a editors have been successfully applied to edit single and multiple genes in the wheat genome (Gil‐Humanes *et al*., 2017; Liang *et al*., 2017; Sánchez‐León *et al*., 2018; Wang *et al*., 2014; Zhang *et al*., 2019), they show relatively low editing efficiency and required transformation of a large number of plants or screening of large populations in the next generation of transgenic plants to recover desired mutations (Wang *et al*., 2018a, 2021). Moreover, the genetic transformation protocols developed for wheat, and for many other crops, are restricted to few varieties showing high regenerative capabilities (Debernardi *et al*., 2020). This reduces the utility of CRISPR technology for the high‐throughput editing of a large number of genes or the direct editing of adapted cultivars for testing the effects of novel CRISPR‐induced alleles in diverse genetic backgrounds. The recent discovery of growth regulators *Baby boom*, *Wuschel* (Lowe *et al*., 2016), and *GRF‐GIF* (Debernardi *et al*., 2020) significantly improved the regeneration efficiency and broadened the range of wheat cultivars amenable to genetic transformation. However, plant transformation remains a time‐ and resource‐consuming effort that requires specialized expertise, limiting its application in most of the breeding or research programmes.

Recently, virus‐based CRISPR delivery systems were developed and tested for several major crops, including wheat (Čermák *et al*., 2015; Ellison *et al*., 2020; Gil‐Humanes *et al*., 2017; Hu *et al*., 2019; Li *et al*., 2021). Compared with the Agrobacterium‐ or biolistic‐based delivery of editing reagents, these systems rely on the natural ability of viruses to spread across the plant cells and allow for omitting the plant transformation and regeneration steps (Baltes *et al*., 2014). A DNA‐based viral replicon was successfully used for delivering CRISPR, Cas9, and DNA templates for homology‐directed repair into tomato plants (Čermák *et al*., 2015). The RNA viruses used for delivering the guide RNAs could maintain the high editing efficiency in the wheat and tobacco lines expressing Cas9 enzymes (Ellison *et al*., 2020; Li *et al*., 2021). The gene editing system based on the barley stripe mosaic virus (BSMV) was capable of generating both somatic and heritable mutations in single or multiple genes in wheat plants expressing Cas9 (Hu *et al*., 2019; Li *et al*., 2021). Because of the ease of viral infection procedure, the virus‐based guide RNA delivery system could potentially be adopted by any genetic research and breeding programmes and scaled up to edit many targets, overcoming the limitations of genetic transformation in wheat and other crops.

Here, we investigated the ability of the BSMV‐based viral system to induce multiple targeted mutations and fragment deletions in the genomes of wheat cultivars carrying the introgression of a high‐expressing allele of the Cas9 gene. By multiplexing BSMV‐gRNA constructs, we created a series of deletions in the promoter of a transcription factor controlling domestication traits in wheat (Simons *et al*., 2006). According to recent studies, CRISPR/Cas9‐induced *cis*‐regulatory mutations in genes controlling productivity traits could broaden the range of phenotypic variation and help to overcome the negative impact of epistasis on major agronomic traits (Rodríguez‐Leal *et al*., 2017; Soyk *et al*., 2017). However, the application of this strategy for mining beneficial *cis*‐regulatory diversity remained limited in crops mostly due to the difficulties associated with the production of *cis*‐regulatory mutants. In our study, we demonstrate that a BSMV‐based viral system is an effective tool for creating new allelic variants in the regulatory regions of the wheat genome. In addition, using wheat lines with the low‐ and high‐expressing Cas9 loci, we investigated the relationship between (i) the levels of Cas9 expression and the efficiency of BSMV‐based genome editing, (ii) the frequency of somatic editing and the heritability of BSMV‐Cas9‐induced mutations, and (iii) the levels of BSMV‐gRNA construct multiplexing and the efficiency of target editing. Our study shows that marker‐assisted introgression of a high‐expressing Cas9 locus provides efficient means for BSMV‐sgRNA‐based genome editing in any adapted cultivar and opens new possibilities for the analysis and discovery of new allelic diversity for crop improvement.

## Results

### Efficiency of BSMV‐based CRISPR editing depends on the Cas9 expression levels

**Figure 1:**
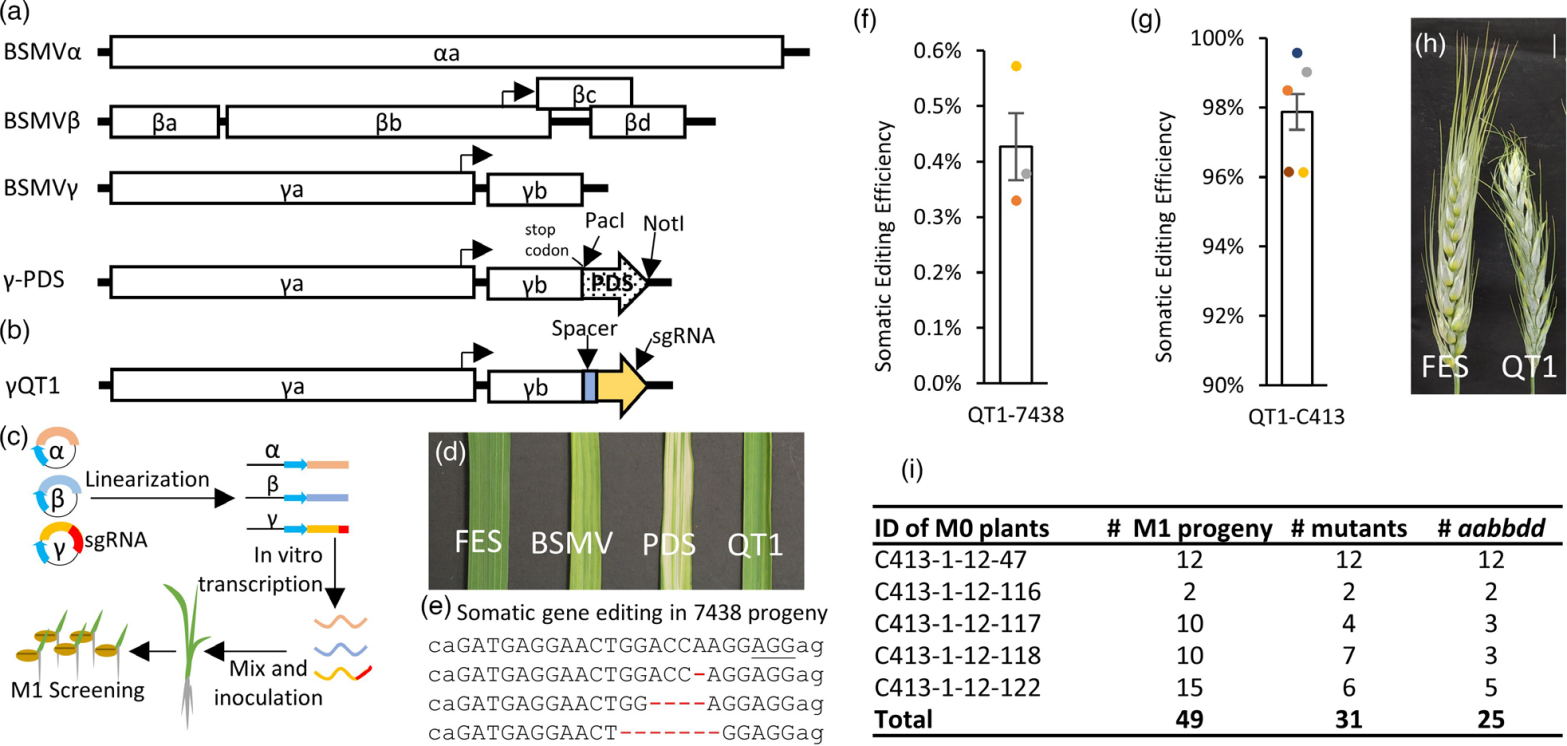
*Q* gene editing using the BSMV‐based sgRNA delivery system. (a) Structure of BSMV and control constructs used for PDS silencing. The open reading frames (ORFs), promoters, and untranslated regions are shown as open boxes, arrows, and hatched boxes, respectively. Orientation of ORFs is depicted by arrow‐shaped boxes. (b) Structure of the gamma chain with QT1 guide targeting the Q gene. (c) BSMV‐gRNA‐based genome editing workflow. (d) The images of the 4th leaf inoculated with the FES buffer, BSMV, BSMV‐PDS, and BSMV‐QT1. The 2nd leaf was used for inoculation. FES buffer is the mock inoculation control; BSMV is the virus without insertion; PDS is BSMV carrying the *PDS* gene fragment; BSMV‐QT1 is the BSMV‐sgRNA construct targeting the *Q* gene. (e) Examples of somatic mutations found in M_0_ plants inoculated with BSMV‐QT1. The AGG PAM sequence is underlined (f) The somatic editing efficiency in 7438‐QT1 plant with low‐Cas9 expression (see Figure S2). (g) The somatic editing efficiency in C413‐QT1 plant with high‐Cas9 expression (see Figure S2). (h) Spike morphology of the wild‐type and mutant plants. The plant showing wild‐type spike morphology was inoculated with FES buffer, whereas the plant with speltoid spikes was inoculated with the BSMV‐QT1 construct. (i) The frequency and genotypes of the *Q* gene mutants are induced by inoculating the C413 line with BSMV‐QT1.

To assess the ability of BSMV transcripts directly applied onto the wheat leaves to deliver sgRNAs into wheat cells and induce mutations, we used a highly efficient QT1 gRNA (Table S1) (Wang *et al*., 2016) previously designed to target the coding regions of the three homoeologous copies of the *Q* gene (*TraesCS5A02G473800*, *TraesCS5B02G486900*, *TraesCS5D02G486600*), which controls major domestication traits in wheat (Simons *et al*., 2006). The Cas9‐induced mutations at the QT1 site are expected to generate the loss‐of‐function *q* alleles, which should result in speltoid spear‐shaped spikes characteristic of the wild relatives of domesticated wheat (Simons *et al*., 2006). The QT1‐sgRNA was subcloned into pBSMVγPDS (Figures 1a and S1) to replace the *TaPDS* gene fragment (henceforth BSMV‐QT1) (Figure 1b). The 2nd leaf of the two‐leaf seedlings from the progeny of a transgenic Bobwhite line (7438) constitutively expressing Cas9 (Wang *et al*., 2016) was inoculated with BSMV‐QT1 (Figure 1c). The leaves of inoculated plants showed white streaks and spots, mosaic symptoms, and necrosis consistent with systemic viral infection (Figure 1d). The next‐generation sequencing (NGS) of the QT1 target site amplicons generated using DNA isolated from the 4th leaf of plants two weeks after inoculation (Figure 1e) was able to detect the Cas9‐induced mutations at the frequency of 0.43% (Figure 1f and Table S2). The target site mutations were not detected in the next generation of these BSMV‐QT1‐inoculated plants. We concluded that the low expression level of Cas9 in transgenic line 7438 was one of the contributing factors to such low editing efficiency (Figure S2).

Measuring the relative levels of Cas9 expression in multiple transgenic Bobwhite lines, we identified lines C413 and 707 (henceforth, high‐Cas9 lines), both expressing Cas9 at levels ~15‐fold higher than line 7438. Five plants derived from the high‐Cas9 line C413 were inoculated with BSMV‐QT1. The high levels of somatic editing efficiency, reaching 98% on average (Figure 1g and Table S3), were detected at the QT1 site in all plants. The recovery of the nonfunctional *q* alleles in these M_0_ plants is also supported by the speltoid spike phenotype (Figure 1h). The NGS of the M_1_ generation plants confirmed the heritability of the BSMV‐induced mutations (Figure 1i). Among 49 analysed M_1_ plants, 31 carried mutations in at least one genome copy and 25 plants were homozygous or heterozygous for mutant alleles in all three homoeologous copies of the *Q* gene (Figure 1i). This result suggests that the high‐Cas9 expression is required to support effective genome editing using sgRNAs delivered via BSMV.

### The efficiency of somatic editing correlates with the heritable mutation rate

**Figure 2:**
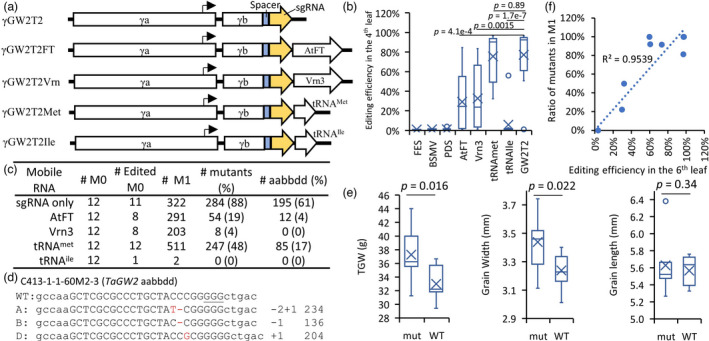
The effects of mobile RNA fusion on gene editing efficiency induced by the BSMV‐sgRNA constructs. (a) The structure of the BSMV‐GW2T2‐based constructs targeting the *TaGW2* gene. The GW2T2‐sgRNA was fused with the *AtFT* (γGW2T2FT), *Vrn3* (γGW2T2Vrn), tRNA^met^ (γGW2T2Met), and tRNA^ile^ (γGW2T2Ile). The open reading frames (ORFs), promoters, and untranslated regions are shown as open boxes, arrows, and hatched boxes, respectively. Orientation of ORFs is depicted by arrow‐shaped boxes. (b) Somatic editing efficiency induced by the BSMV‐ sgRNA constructs with and without mobile RNA in the noninoculated 4th leaf of the C413 line. The mutation frequency at the GW2T2 target was calculated by combining data from all three genomes. The significance of differences between the respective gRNAs with and without mobile RNAs was tested using the Student's *t*‐test (*P*‐values are shown above the plot). (c) The summary of BSMV‐sgRNA inoculation experiment showing the number of inoculated plants and the frequency of mutations in *TaGW2*. The somatic mutations in the M_0_ generation were evaluated using DNA extracted from the noninoculated 4th leaf. The number of mutants in the M_1_ generation corresponds to the number of plants carrying mutations in at least one homoeolog of *TaGW2*. The genotypes of triple *TaGW2* mutants with mutations in each of the homoeologous wheat genomes A, B, and D are shown as lower‐case letters *aabbdd*. (d) The alignments of representative NGS reads from M_1_ plant C413‐1‐1‐60 M2‐3, which carries mutations in each of the homoeologous copies of *TaGW2* from three wheat genomes. The level of divergence between the wheat genomes allows for homoeolog‐specific alignment of NGS reads. The deleted and inserted nucleotides are shown in red. The number of deleted (−1 or −2) or inserted (+1) nucleotides are shown on the right of each read type. The number of wild‐type (WT) and mutated reads aligned to each genome is shown on the right side of mutation types. The PAM sequence is underlined. (e) Phenotypic effects of BSMV‐sgRNA induced mutations in *TaGW2*. The thousand grain weight (TGW), grain width, grain length of triple mutants (*aabbdd*, *n* = 7), and wild‐type plants (*AABBDD*, *n* = 7) are compared. The Student's *t*‐test *P*‐values are shown above the plot. (f) Relationship between the proportion of *TaGW2* mutants in the M_1_ generation and somatic editing efficiency evaluated in the 6th leaf at the booting stage of M_0_ plants. Each data point stands for an individual plant.

It was successfully demonstrated that improvement in gene editing efficiency could be achieved by targeting sgRNAs to shoot apical meristem cells using mobile RNAs (Ellison *et al*., 2020). In these experiments, the endogenous mobile RNA encoded by the *FT* gene was used (Li *et al*., 2011). However, prior studies of mobile RNA fusions on genome editing efficiency produced contradicting results in different plant species (Ellison *et al*., 2020; Li *et al*., 2021). We decided to re‐evaluate the effect of mobile RNAs on editing efficiency in our experimental system by fusing Arabidopsis *FT* (*AtFT*), wheat *Vrn3* (Yan *et al*., 2006), and methionine and isoleucine tRNAs (tRNA^met^ and tRNA^ile^) with the sgRNA targeting the *TaGW2* gene (Figures 2a and S1), which affects grain size and weight in wheat and rice (Song *et al*., 2007; Wang *et al*., 2018b). Compared with the nonfused BSMV‐GW2T2 construct, which showed 77% editing efficiency in inoculated leaves (Figures 2b and S3, Table S4), *AtFT*, *Vrn3*, and tRNA^ile^ fusions, except tRNA^met^, had substantially reduced somatic editing efficiency (29%, 33%, and 6%, respectively). The tRNA^met^ fusion resulted in a minor efficiency reduction reaching 76% compared with nonfused BSMV‐GW2T2. Consistent with these results, gene editing efficiency reduction caused by mobile RNA fusion was also observed for a sgRNA targeting the *PDS* gene in the wheat study (Li *et al*., 2021).

By genotyping the M_1_ generation, we show that the proportion of M_1_ plants with inherited target site mutations correlated with the frequency of somatic mutations detected in the leaves of the M_0_ plants. The BSMV‐GW2T2 produced the highest number of mutants in M_1_ generation (88%), followed by BSMV‐GW2T2‐tRNA^met^ (48%) and BSMV‐GW2T2‐AtFT (19%). The proportion of M_1_ mutants created using BSMV‐GW2T2 with all six copies of *TaGW2* mutated (Figure 2d) reached 61%. Consistent with our previous report (Wang *et al*., 2018c), the *TaGW2* mutants had significantly increased grain weight (13%) and width (6%) compared with wild‐type plants (Figure 2e), though we did not detect significant changes in grain length. We assessed the frequency of somatic mutations induced by BSMV‐GW2T2 and BSMV‐GW2T2‐tRNA^met^ using DNA isolated from the 2nd (inoculated leaf), 4th and 6th leaf. The frequency of somatic editing positively correlated with the frequency of M_1_ mutants in the 6th leaf, while a less obvious correlation was observed in the 2nd and 4th leaves (Figures 2f and S4).

### Application of pooled BSMV‐sgRNAs for editing multiple genes and generating promoter deletions

**Figure 3:**
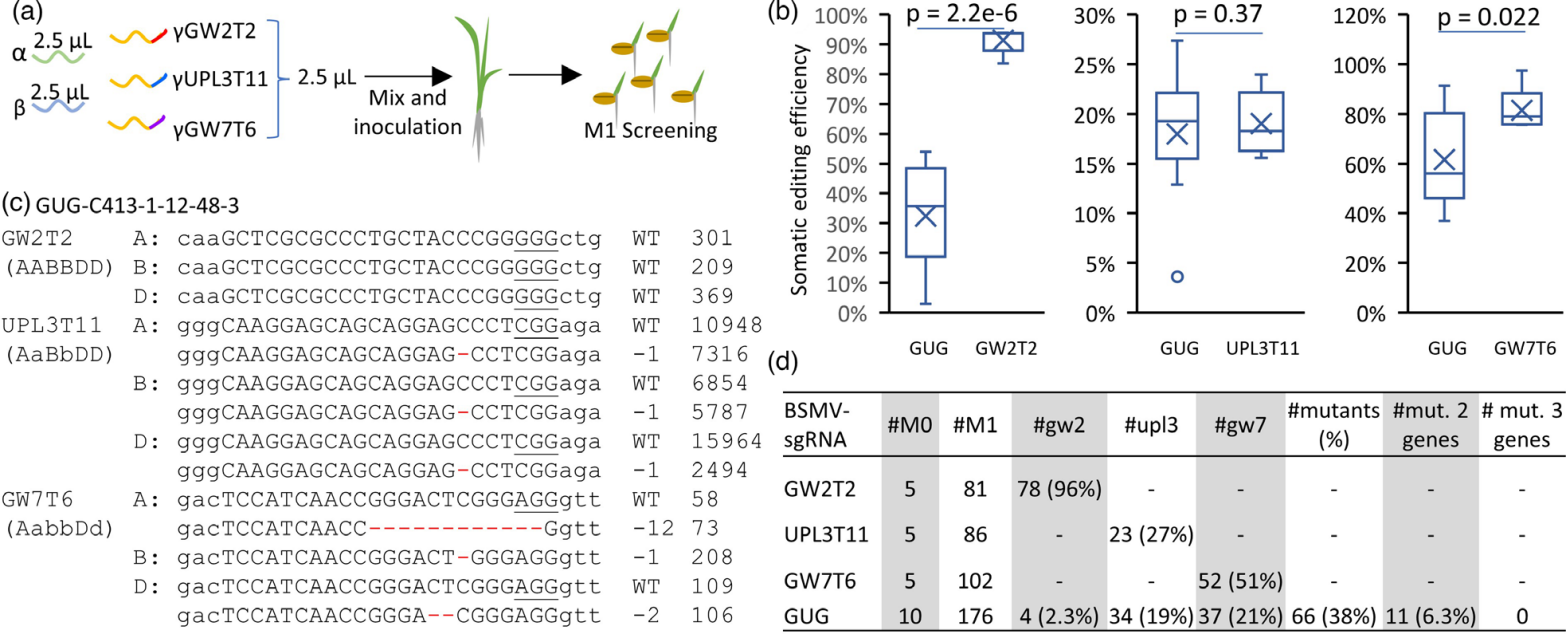
Multiplex gene editing by inoculating with the pooled BSMV‐sgRNAs. (a) A schematic pipeline of the BSMV‐based multiplex editing using the sgRNA pool targeting *TaGW2* (GW2T2‐sgRNA), *TaUPL3* (UPL3T11‐sgRNA), and *TaGW7* (GW7T6‐sgRNA). (b) Comparison of somatic editing efficiency at each target site in plants inoculated with the BSMV‐GUG pool or individual BSMV‐sgRNAs. The data from all three genomes were combined to calculate the somatic editing efficiency. The multiplex and single target editing efficiencies were compared using the Student's *t*‐test (*P*‐values are shown above the plots); NS – *P* > 0.05, * – *P* < 0.05, *** – *P* < 0.0001. (c) Alignment of representative NGS reads from the M_1_ plants derived from the BSMV‐GUG inoculated plants. The NGS reads were aligned to the A, B, and D homoeologs of the *TaGW2*, *TaUPL3*, and *TaGW7T6* genes. The deleted nucleotides are shown in red. The number of deleted nucleotides is shown on the right of the reads. The number of wild‐type (WT) and mutated reads is shown on the right side of mutation types. The PAM sequences are underlined. (d) The number of plants carrying mutations in one, two, or all three genes obtained by inoculating with BSMV‐GUG and the individual BSMV‐sgRNAs.

Previously, it was demonstrated that the BSMV‐based system could be used to simultaneously edit multiple loci in wheat (Li *et al*., 2021). However, it remained unclear whether the efficiency of editing is affected by the BSMV‐sgRNA pooling compared with the individual BSMV‐sgRNA constructs. We performed multiplex editing by inoculating the high‐Cas9 lines with the pool of RNAs synthesized from the BSMV‐sgRNA constructs targeting the regions of *TaGW2*, *TaUPL3* and *TaGW7* conserved across all three wheat genomes (henceforth, the BSMV‐GUG pool) (Figure 3a). Compared with plants inoculated with the BSMV‐sgRNAs targeting a single gene, the BSMV‐GUG pool showed lower somatic editing efficiency for target sites GW2T2 and GW7T6, while the efficiency of UPL3T11 editing remained the same (Figures 3b and S5, Table S5). The number of mutants identified in the M_1_ generation of plants inoculated with the pooled and individual BSMV‐gRNAs showed similar trends (Figure 3c,d). The proportion of plants with mutations at the GW2T2, UPL3T11, and GW7T6 target sites dropped from 96% to 2.3%, from 27% to 19%, and from 51% to 21%, respectively. Only 5.7% of M_1_ plants carried mutations at two target sites, and no mutants with all three targets edited were recovered (Figure 3c). These results suggest that with an increase in the multiplexing level performed by simple pooling of BSMV‐sgRNAs, we should expect a decrease in the efficiency of editing of individual targets and the rate of multiplex gene editing.

**Figure 4:**
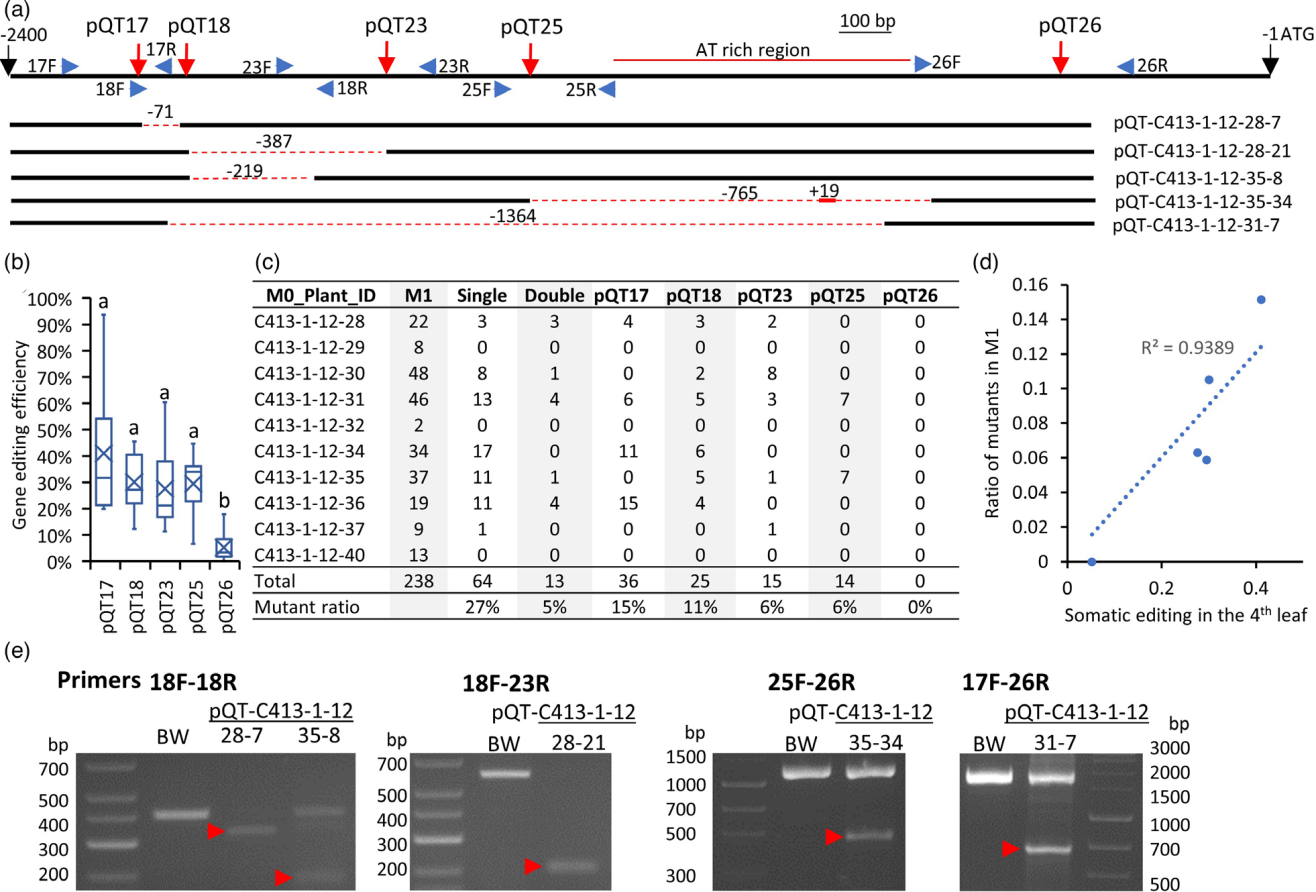
*Q* gene promoter editing using the pool of BSMV‐sgRNAs (BSMV‐pQT). (a) The location of the BSMV‐sgRNA target sites (red arrows) across the *Q* gene promoter region extending 2400 bp upstream of the start codon (ATG). The location of PCR primers used for genotyping is indicated by blue arrows. The location of an A/T‐rich region is shown by a red line. The deletions and insertions detected in five M_1_ plants are shown as red dashed and solid lines, respectively, with the sizes of deletions and insertions shown above. (b) The somatic editing efficiency measured in the 4th leaf (developed after inoculation) of plants inoculated with the BSMV‐pQT pool. The results of the groups' mean comparisons using the Tukey post hoc test in one‐way ANOVA are shown above the boxplots. (c) The summary of mutations discovered in the M_1_ generation plants inoculated with the BSMV‐pQT pool. (d) The relationship between the somatic editing efficiency and the proportion of mutants discovered in the M_1_ generation plants. Each data point stands for one BSMV‐sgRNA target site. (e) The results of PCR‐based screening of the M_1_ generation plants for deletions in the promoter region of the *Q* gene promoter. A total of 11 pairs of primers (17F‐18R, 17F‐23R, 17F‐25R, 17F‐26R, 18F‐18R, 18F‐23R, 18F‐25R, 18F‐26R, 23F‐25R, 23F‐26R, and 25F‐26R) were used in screening. The cultivar Bobwhite (BW) was used as a wild‐type control. The PCR fragments corresponding to deletions are shown by red arrows.

Previously, the multiplexed genome editing strategy was used to generate variation in the promoters of genes controlling productivity traits (Rodríguez‐Leal *et al*., 2017). Here, we tested the ability of the pooled BSMV‐sgRNAs to create deletions in the promoter of the *Q* gene allele on chromosome 5A (Figure 4a and Table S1), which controls a number of domestication traits in wheat (Debernardi *et al*., 2017; Simons *et al*., 2006). Its expression levels positively correlate with compact spike morphology, free‐threshing habit, and rachis fragility. In addition to domestication traits, the *Q* allele was linked with increased spike length, number of spikelets per spike, and number of grains per spikelet and spike (Zhang *et al*., 2020). By editing the *Q* gene's promoter region, we might remove regulatory elements that have positive or negative effects on gene expression levels or regulate distinct biological pathways controlled by the *Q* gene. The *Q* gene promoter region on chromosome 5A was highly divergent from the promoter regions on homoeologous chromosomes 5B and 5D (Figure S6). The Sanger sequencing of the 3 kb region upstream of the *Q* gene region revealed that cultivar Bobwhite has one SNP, one 160 bp deletion, and one 160 bp insertion compared with the reference genome of cultivar Chinese Spring (The International Wheat Genome Sequencing Consortium (IWGSC), 2018) (Figure S7). The *Q* gene promoter was edited by inoculating the high‐Cas9‐expressing line C413 with a pool of five BSMV‐sgRNA transcripts (henceforth, BSMV‐pQT).

The means of somatic editing efficiency for each of the five target sites, including pQT17, pQT18, pQT23, pQT25, and pQT26, were 37%, 28%, 25%, 27%, and 5%, respectively (Figure 4b, Table S6). The proportions of M_1_ plants carrying heritable mutations at each target site were 15%, 11%, 6%, 6%, and 0%, respectively (Figure 4c), and highly correlated with somatic editing efficiency (Figure 4d). About 32% of M_1_ plants carried heritable mutations in at least one target site. There were no M_1_ plants carrying heritable mutations at three or more targets. A total of 5% of M_1_ plants had heritable mutations at two target sites, which is nearly 5 times lower than the proportion of M_1_ plants with mutations at a single target site (27%) (Figure 4c).

Further, we screened the edited plants for deletions spanning the regions between the pairs of the BSMV‐sgRNA target sites. First, we tested M_0_ plants for deletions between target sites pQT17 and pQT18 by performing PCR using a pair of flanking primers 17F and 18R (Figure 4a). The NGS of amplicons showed that 5.2%–19.9% of reads carried the 71‐bp deletion expected upon successful targeting of both the pQT17 and pQT18 sites (Figure 4a, Table S7).

The *Q* gene promoter deletion screening in M_1_ plants was performed using 11 pairs of PCR primers (Table S1). We identified five M_1_ plants that had deletions in the homozygous or heterozygous state or presented as mosaic somatic mutations (Figure 4a,e). Two of these plants, pQT‐C413‐1‐12‐28‐7 and pQT‐C413‐1‐12‐28‐21, were homozygous for deletions between the pQT17 and pQT18 target sites and between the pQT18 and pQT23 target sites, respectively (Figure 4a).

Compared with wild‐type plants, these two deletion mutants did not exhibit visible differences in development or spike morphology (Figure S9). The plants with deletions in heterozygous state or present as nonfixed somatic mutations also did not show visible phenotypic effects on spike morphology. The quantitative RT‐PCR analysis of *Q* gene expression in the 3rd and 6th leaves at the 6‐leaf developmental stage and in the spikes of M_1_ plants also did not detect significant changes compared with the wild‐type plants (Figures S8–S10). The lack of expression changes in mutants could be linked to the lack of overlap between the induced promoter mutations and regulatory regions controlling *Q* gene expression. Alternatively, promoter mutations led to changes in the *Q* gene expression domain without affecting absolute *Q* expression levels. It is also possible that the phenotypic effects of promoter mutations are small and require larger population sizes to detect subtle changes in spike morphology. Further studies aimed at creating additional deletions within the upstream regulatory region of the *Q* gene and more extensive phenotypic evaluation of created mutants are planned in the future.

### BSMV‐sgRNA‐based gene editing in the winter and spring wheat cultivars

**Figure 5:**
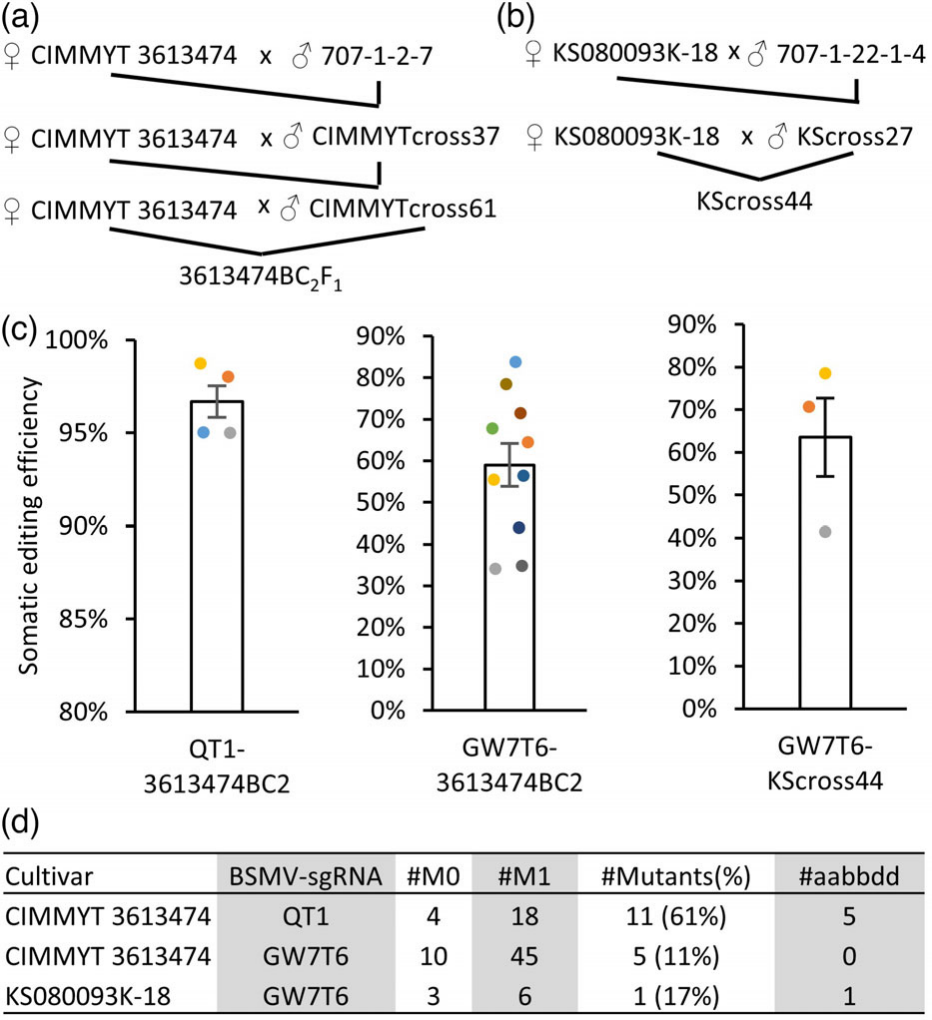
BSMV‐sgRNA‐based gene editing of the spring and winter cultivars. Introgression of the highly expressed Cas9 allele from the high‐Cas9 707 line (Figure S1) into (a) CIMMYT line 3 613 474 (spring wheat) and (b) Kansas line KS080093K‐18 (winter wheat). The BC2F3 and BC1F2 lines derived from the crosses with 3 613 474 and KS080093K‐18, respectively, were used for BSMV inoculation. (c) The somatic editing efficiency was estimated (1) for BSMV‐QT1 and BSMV‐GW7T6 constructs used to inoculate lines derived from 3 613 474 and (2) for BSMV‐GW7T6 used to inoculate lines derived from KS080093K‐18. The editing efficiency was evaluated in the 4th leaf of inoculated plants. The data from all three genomes are combined to calculate the editing efficiency. (d) The summary of the M_1_ generation mutants obtained by the BSMV‐sgRNA‐based editing of wheat cultivars expressing the introgressed high‐Cas9 allele.

Even though the BSMV‐based method of sgRNA delivery method bypasses the wheat transformation and regeneration steps (Li *et al*., 2021), it still depends on the availability of a wheat line expressing the Cas9 gene. Despite recent advances in the wheat regeneration methodology (Debernardi *et al*., 2020), the development of transgenic wheat cultivars remains a time‐consuming process and has varying levels of success. To broaden the range of wheat cultivars amenable to editing using the BSMV‐based sgRNA delivery system, we transferred the highly expressed allele of Cas9 from line 707 into elite spring wheat line 3 613 474 from CIMMYT and winter wheat line KS080093K‐18 from Kansas wheat breeding programme (Figure 5a,b). The backcrossed progeny of both lines, now carrying the highly expressed allele of Cas9, was inoculated with the RNA transcripts synthesized from the BSMV‐sgRNA constructs targeting the *Q* and *TaGW7* genes. Similar to the results obtained by inoculating the high‐Cas9 C413 line with BSMV‐QT1 (Figure 1), the BC_2_F_3_ plants from the 3 613 474 families inoculated with BSMV‐QT1 also exhibited spear‐shaped speltoid spikes suggestive of the presence of the loss‐of‐function mutations in the *Q* gene. Consistent with this result, a high level of somatic editing (97%) was observed for the QT1 target in these plants (Figures 5c and S11, Table S8). In the M_1_ generation, 61% of plants carried mutations in at least one homoeologous copy of the *Q* gene with nearly 30% of plants carrying mutations in all three homoeologs of the *Q* gene (Figure 5d). The plants from the 3 613 474 and KS080093K‐18 backcross families inoculated with BSMV‐GW7T6 showed 59% and 64% somatic editing efficiency, respectively (Figure 5c). In the M_1_ generation, 11% and 17% of 3 613 474‐ and KS080093K‐18‐derived plants inherited mutations in the *TaGW7* gene, respectively (Figure 5d).

## Discussion

Our study shows that the efficiency of the BSMV‐based editing and the chance of obtaining heritable mutations depend on the sgRNA design and the levels of Cas9 expression. This dependency holds for both single BSMV‐sgRNA and multiplexed BSMV‐sgRNA constructs, indicating that the development of the transgenic wheat line with adequate levels of Cas9 expression is critical for successful BSMV‐sgRNA‐based editing. Once a high‐Cas9 line is developed, it can be used either directly in gene editing experiments or as a donor of the high‐expressing Cas9 allele for introgression into other cultivars.

We showed that somatic editing frequency in the M_0_ plants inoculated with the BSMV‐sgRNA transcripts correlates with the frequency of heritable mutations in the M_1_ generation. It appears that in the BSMV‐sgRNA‐based gene editing experiments we can skip the step of protoplast‐based testing of the sgRNA editing efficiency (Wang *et al*., 2019), and instead directly assess the sgRNA editing efficiency by sequencing the DNA extracted from the newly grown seedling leaves of the BSMV‐sgRNA inoculated plants. Such early assessment of somatic editing efficiency can help to quickly adjust ongoing experiments. For example, in case of discovering that a critical sgRNA has low editing efficiency, the number of the BSMV‐sgRNA inoculated plants could be increased to increase the heritable mutation recovery rate or sgRNA could be re‐designed to improve its efficiency in the follow‐up experiments.

A previous study (Li *et al*., 2021) show that the multiplex wheat genome editing is possible with the pooled BSMV‐sgRNAs. Our results indicated that the level of multiplexing for achieving simultaneous editing of all targets could be limited. The likelihood of recovering mutations at multiple sites across a genome appears to be the product of the editing efficiency of individual targets. Probably this happens because the chance for the same plant cell to be infected by multiple BSMV particles with distinct guides is reduced with an increase in the level of multiplexing. Superinfection exclusion could be another factor contributing to the reduced efficiency of multiplexed editing by preventing secondary infection of plant cells already infected by one BSMV particle, thereby reducing the probability of getting a genome of the same cell edited by multiple gRNAs. For effective editing of multiple targets using the BSMV‐sgRNA‐based method, we should explore alternative strategies. One of the possible approaches is to create BSMV‐sgRNA constructs expressing an array of multiple guide RNAs simultaneously. However, the level of multiplexing in this case will likely be limited by the restrictions on the size of a genome that could be packed into a viral particle. This could partially be overcome by arraying sgRNAs in a single viral vector because prior studies suggest that no processing signals are required to separate sgRNA units in the viral transcripts (Cody *et al*., 2017; Ellison *et al*., 2020). The usage of both the β and γ chains of the BSMV genome for subcloning the sgRNAs or development of the BSMV‐sgRNA system based on the Cas12a editors (Wang *et al*., 2021; Zetsche *et al*., 2015), which utilize much shorter guides, could also be used for expanding the multiplexing capacity of the BSMV‐sgRNA‐based editing system.

The utility of CRISPR‐induced *cis*‐regulatory diversity for improving productivity traits was clearly demonstrated in tomato (Rodríguez‐Leal *et al*., 2017). In our study, we showed that the multiplexed BSMV‐sgRNA‐based system could be an effective tool for creating deletions in the upstream regulatory region of the *Q* gene. The approach was highly effective and allowed us to quickly recover deletion variants in the next generation of the BSMV‐sgRNA‐inoculated wheat lines. The distribution of editable sites that could be targeted by Cas9 in the *Q* gene promoter did not allow us to create deletions near the transcription start site and recovered mutants did not show significant changes in the gene expression level or phenotype. Nevertheless, our results demonstrate that this editing strategy opens opportunities for exploring novel *cis*‐regulatory diversity in the wheat genome and modulating the regulatory effects of transcription factors controlling agronomic traits. One of the shortcomings of pooling multiple BSMV‐sgRNAs was the reduction in the efficiency of individual target editing, which resulted in the relatively low frequency of any given deletion variant. To increase the chance of recovering specific deletions using the BSMV‐sgRNA‐based system, the reduced levels of multiplexing should be considered (e.g. pooling only two BSMV‐sgRNAs targeting sites flanking the region of interest). Alternatively, as was discussed above, constructs incorporating multiple guides into a single viral transcript need to be designed (Cody *et al*., 2017; Ellison *et al*., 2020; Wang *et al*., 2021; Zetsche *et al*., 2015).

We showed that introgression of the high‐expressing Cas9 locus into a target cultivar can maintain the effective BSMV‐sgRNA‐induced genome editing, removing the cultivar‐specific constraints on targeted genetic modification of the wheat genome associated with transformation‐based approaches. Introgression of Cas9 locus and recovery of up to 98% recurrent parent genome in crosses could be quickly accomplished in three rounds of backcrossing using locus‐specific and genome‐wide markers (Hospital, 2003). As soon as the donors of high‐expressing Cas9 lines and a virus‐based CRISPR delivery system are available (Ellison *et al*., 2020; Li *et al*., 2021; Yin *et al*., 2015), a similar gene editing approach could be used for modifying the genomes of other crops. Contrary to approaches that are based on random genome‐wide chemical mutagenesis of thousands of lines (Henry *et al*., 2014; Krasileva *et al*., 2017), these functional screens can be used to analyse the effects of mutations without the confounding impact of mutations in other parts of the genome. Considering the ease with which the BSMV‐sgRNA constructs can be designed to target hundreds of targets and the simplicity of BSMV‐sgRNA inoculation procedure, lines with the Cas9 introgression provide an opportunity for establishing the high‐throughput functional screens for genetic studies aimed at identifying causal mutations or novel variants affecting agronomic traits.

## Methods

### Plasmid construction

The previously reported plasmids (Scofield *et al*., 2005) with the α, β, and γ chains of the ND18 strain of barley stripe mosaic virus (BSMV), referred to as pBSMVα, pBSMVβ and pBSMVγ, were used in our study (Figure 1a). The virus‐induced gene silencing (VIGS) constructs pBSMVγPDS previously developed for targeting the barley phytoene desaturase gene (*HvPDS*) was used in our study to silence *TaPDS* in wheat (Holzberg *et al*., 2002; Scofield *et al*., 2005) (Figure 1a). For delivering sgRNAs into plant cells using BSMV, the sgRNA sequences were inserted into the pBSMVγPDS plasmid after removing the *PDS* fragment by PacI and NotI digestion (New England BioLabs, Ipswich, MA, catalogue numbers R0547 and R0189). The sequences targeted in our study are listed in Table S2. All the primers, DNA oligos and double‐stranded DNA fragments are synthesized by Integrated DNA Technologies (Coralville, IA) (Table S1). PCR reactions were performed using NEBNext® High‐Fidelity 2X PCR Master Mix (New England BioLabs, catalogue number M0541) following the manufacturer's protocol unless specified differently. For example, to insert the sgRNA for target QT1 into pBSMVγPDS, the sgRNA was amplified from the pU6sg‐QT1 construct (Wang *et al*., 2016) using a primer pair targetVIGSsF and targetVIGSsR. The PCR fragments and the plasmid pBSMVγPDS were digested using PacI and NotI; the PCR fragment and the plasmid fragment without *PDS* gene were isolated from agarose gel using QIAquick Gel Extraction Kit (Qiagen, catalogue number 28706). The digested PCR products and the pBSMVγ plasmid were ligated using T4 ligase (New England BioLabs, catalogue number M0202). This plasmid was named pBSMVγQT1 (Figure 1b). All inserted DNA fragments were validated by Sanger sequencing. All the Sanger sequencing in this study was done using BigDye™ Terminator v3.1 Cycle Sequencing Kit (ThermoFisher Scientific, catalogue number 4337455).

To test the effect of mobile RNAs on BSMV‐sgRNA‐induced gene editing, the fragments including GW2T2‐sgRNA and mobile RNAs were synthesized at Integrated DNA Technologies (Table S1). The mobile RNAs included the coding sequences of *FT* gene from *Arabidopsis* (Ellison *et al*., 2020) and its wheat ortholog *Vrn3* (Yan *et al*., 2006), and wheat methionine and isoleucine tRNAs tRNA^Met^ and tRNA^Ile^ (Figure 2a). The GW2T2‐sgRNA without mobile elements were amplified from the previously developed pBUN421‐GW2T2 plasmid (Wang *et al*., 2018a) using the primer pair VIGS‐GW2T2‐sgRNA‐F and VIGS‐sgRNA‐R. Both the synthesized DNA fragment and the PCR products were subcloned into PacI‐ and NotI‐digested pBSMVγPDS using NEBuilder® HiFi DNA Assembly Master Mix (New England BioLabs, catalogue number E2621) following the manufacturer's protocol.

The UPL3T11‐sgRNA with flanking sequences overlapping with the PacI‐ and NotI‐digested pBSMVγPDS (Table S1) were synthesized at Integrated DNA Technologies. The sgRNAs with flanking sequences for GW7T6, pQT17, pQT18, pQT23, pQT25, and pQT26 were obtained by amplifying the sgRNA scaffold of the pBUN421 plasmid (Xing *et al*., 2014) using the forward primers paired with reverse primer VIGS‐sgRNA‐R (Table S1). Both synthesized DNA fragments and PCR products were subcloned into PacI‐ and NotI‐digested pBSMVγPDS using NEBuilder® HiFi DNA Assembly Master Mix.

### Sequencing of the *Q* gene promoter in cv. Bobwhite

Three pairs of primers (Table S1) targeting overlapping genomic regions were used to amplify the *Q* gene promoter in cv. Bobwhite. The PCR fragments of primer pair pQT4MiF – pQT17MiR and primer pair pQT17MiF – pQT25MiR were subcloned into pCRblunt using Zero Blunt™ TOPO™ PCR Cloning Kit (ThermoFisher Scientific, catalogue number 450245) following the manufacturer's protocol. For each subcloned fragment, three colonies were sequenced. The PCR product of primer pair pQT25checkF – Q5endR3 was sequenced directly without subcloning.

### Plants and growth conditions

A transgenic line 7438 expressing previously reported wheat‐codon‐optimized Cas9 (Figure S12) in the background of cv. Bobwhite (Wang *et al*., 2016) was utilized for the initial testing of BSMV‐sgRNA‐based gene editing. Later, to improve the BSMV‐based gene editing efficiency, additional transgenic lines in the background of cv. Bobwhite was screened to identify plants with high levels of Cas9 expression (high‐Cas9). The 707 and C413 lines expressing maize‐codon‐optimized Cas9 (Figure S12) at a high level were used for further research (Figure S12). The 707 line was used as a donor of the high‐Cas9 allele for introgression into CIMMYT spring wheat 3 613 474 and Kansas winter wheat KS080093K‐18. These two lines were crossed with 707, and then, Cas9‐positive F_1_ plants were selected by PCR using the primer pair zCas9‐F and zCas9seq1 (Table S1) and backcrossed one and two times to KS080093K‐18 and 3 613 474, respectively. The Cas9‐positive lines from the backcrossed plants were used for BSMV‐sgRNA inoculation.

All plants were grown in ¼ litter square pots filled with the Berger BM1 growing mix (Hummert, catalogue number 10120000) in a growth chamber at 24 °C day/20 °C night conditions with 16 h supplemental light. The plants were grown in ¼ litter square pots for two weeks after inoculation and then transplanted into 1 L square pots. The M_1_ generation plants were grown in the 128‐well plastic trays filled with the Berger BM1 nutrient retention soil.

### In vitro transcription of BSMV‐sgRNA constructs and plant inoculation

The α, β and γ virus chains were transcribed in vitro from plasmids pBSMVα, pBSMVβ, and pBSMVγ linearized by digesting with MluI (New England BioLabs, catalogue number R3198S), SpeI (New England BioLabs, catalogue number R0133S), and MluI enzymes, respectively. The pBSMVγPDS plasmid and all plasmids expressing BSMV γ chain carrying sgRNAs were linearized with BssHII (New England BioLabs, catalogue number R0199S). In vitro transcription was performed in 20 μL reaction volume using HiScribe™ T7 High Yield RNA Synthesis Kit (New England BioLabs, catalogue number E2040S) following the Capped RNA Synthesis protocol provided by the manufacturer. The m7G(5′)ppp(5′)G RNA Cap (New England BioLabs, catalogue number S1404L) was used as Cap Analog with 4:1 of Cap Analog:GTP ratio. The quality and concentration of RNA transcripts (usually within 2–2.5 μg/μL range) were assessed on the agarose gel.

The second leaf of wheat seedlings at the two‐leaf stage was inoculated with the FES buffer, BSMV, BSMV‐PDS, or BSMV‐sgRNAs. Each plant was inoculated using the mixture of 60 μL FES buffer and transcription products of BSMV α, β and γ chain (2.5 μL each). The FES buffer includes 7.51 g/L glycine, 10.45 g/L K_2_HPO_4_ dibasic, 10 g/L sodium pyrophosphate decahydrate, 10 g/L bentonite, and 10 g/L celite. The inoculation was performed by hand‐rubbing the 2nd leaf from the base to the tip while wearing clean nitrile gloves. The procedure was repeated 3 times for each plant, each time applying 20 μL of the mixture. Immediately after inoculation, the plants were covered by plastic bags to keep the moisture. Plastic bags were removed 5–7 days after inoculation. In each experiment, three groups of controls including FES buffer, wild‐type BSMV, and BSMV‐PDS were used. The virus infection symptoms were usually observed 7 days after inoculation, while the PDS RNAi phenotype appeared 12 days after the inoculation.

### Genotyping of BSMV inoculated plants and screening of mutants in M1 generation

To detect target editing events, a 2 cm‐long segment of the 4th leaf was sampled about two weeks after inoculation when BSMV‐PDS‐inoculated control plants showed photobleached leaf symptoms. DNA was isolated using the TPS buffer (100 mm Tris–HCl, 10 mm EDTA, 1 m KCl, pH8.0) as described (Wang *et al*., 2021). The gene editing efficiency in the M_0_ and M_1_ plants was assessed for each subgenome separately using the next‐generation sequencing (NGS) approach, as previously described (Wang *et al*., 2018a). We used the following NGS read coverage thresholds to call the genotypes at the edited gene loci in M_1_ plants. If the proportion of NGS reads with edited alleles was between 30% and 70%, the locus was defined as heterozygous. The homozygous genotype calls had more than 90% of NGS reads carrying either wild‐type or mutated variants. Any M_1_ plant was considered as edited if it had at least one subgenome carrying heterozygous or homozygous mutations.

The deletions in the *Q* gene promoter region were detected using various combinations of PCR primers (Figure 4a,e, Table S1) listed in. The examples of agarose gel images with PCR products showing evidence of deletions in the *Q* gene promoter are shown in Figure 4.

### RNA isolation and qPCR

RNA isolation was performed using the TRIZOL reagent (ThermoFisher Scientific, catalogue number 15596026) following the manufacturer's protocol. The cDNA was obtained by reverse transcription using SuperScript™ III First‐Strand Synthesis SuperMix for qRT‐PCR (ThermoFisher Scientific, catalogue number 11752050). The Cas9 expression in the transgenic plants was measured by qRT‐PCR using the primer pair zCas9‐F and zCas9seq1 and RNA isolated from the 2nd leaf of two‐week‐old seedlings. The *Q* gene expression in the M_1_ plants was measured by isolating RNA from the 3rd and 6th leaf at the 6‐leaf development stage. The samples were frozen in liquid nitrogen immediately and then stored under –80 °C until RNA was isolated. The qPCR reaction was performed using the PowerUP SYBR Green Master Mix (ThermoFisher Scientific, catalogue number A25741) following the manufacturer's protocol. NEBNext® High‐Fidelity 2X PCR Master Mix (New England BioLabs, catalogue number M0541) and SybrGreen were used to assess the expression level of the Q gene on chromosome 5A. The specificity of the Q gene primers, Q5ArtF4 and Q5ArtR4 (Table S1), was validated using nullisomic‐tetrasomic genetic stocks (Figure S10). The *TaActin* gene expression was used as a reference.

## Conflict of interest

The authors declared that they do not have conflicts of interest.

## Author contributions

W.W. designed and conducted gene editing experiments, genotyped and phenotyped transgenic plants, collected and analysed phenotyping data, and helped with drafting of the manuscript; Z.Y. conducted gene editing experiments; F.H. contributed to the analysis of NGS data; G.B. performed Sanger sequencing; H.T. coordinated the biolistic transformation part of the project; A.A. designed and conducted NGS experiments; E.A. conceived the idea, interpreted the results, coordinated the project, and wrote the manuscript. All authors read the manuscript and approved the final version.

## Supporting information


**Figure S1** The sequences of barley stripe mosaic virus (BSMV) plasmids used in the study.
**Figure S2** The expression level of Cas9 in transgenic plants.
**Figure S3** Somatic editing induced by the BSMV‐sgRNA constructs with and without mobile RNA in the noninoculated 4th leaf of the C413 line.
**Figure S4** Relationship between the mutagenesis ratio in the M1 progeny of plants inoculated by BSMV‐GW2T2 (a, b, and d) or BSMV‐GW2T2 (c and e) and the somatic editing efficiency evaluated in the 6th (a), 4th (b and c), and 2nd (d and e) leaf.
**Figure S5** The somatic editing efficiency of each target site in the 4th leaf (developed after inoculation) of plants inoculated by multiplex editing pool (BSMV‐GUG) or BSMV carrying single guide RNAs.
**Figure S6** The alignment of the *Q* gene promoter region from the A, B, and D genomes along with the CRISPR‐Cas9 targets.
**Figure S7** Alignment of the *Q* gene (chromosome 5A) promoter from cultivars Chinese Spring and Bobwhite.
**Figure S8** Expression of the *Q* gene's A genome homoeolog in the M1 plants at the 6‐leaf stage.
**Figure S9** The *Q*‐5A gene expression level in spikes of M2 plants with edits in the *Q*‐5A promoter.
**Figure S10** Validation of the A genome‐specific primers for RT‐PCR of the *Q* gene.
**Figure S11** The somatic editing efficiency of BSMV‐QT1 and BSMV‐GW7T6 in line 3 613 474 and the somatic editing efficiency of BSMV‐GW7T6 in KS080093K‐18.
**Figure S12** The Cas9 plasmids used for creating transgenic plants.
**Table S1** The primers, oligos, and synthesized double‐strand DNA used in this study.
**Table S2** The target sites selected for BSMV‐sgRNA‐based editing in this study.
**Table S3** The efficiency of the *Q* gene (chromosome 5A) editing based on the BSMV‐sgRNA delivery system in transgenic wheat lines with the low (7438) and high (C413) levels of Cas9 expression.
**Table S4** The efficiency of somatic editing in the wheat leaves inoculated with the BSMV transcripts carrying the TaGW2 gRNA with and without mobile elements.
**Table S5** Somatic editing efficiency of individual and pooled (GUG) BSMV transcripts.
**Table S6** Somatic editing efficiency in the promoter region of the *Q* gene (chr. 5A).
**Table S7** Proportion of NGS reads carrying the 71‐bp deletion.
**Table S8** Somatic editing efficiency of targets in the background of other cultivars.
