# Supplementary material for: Multiplexed promoter and gene editing in wheat using a virus‐based guide RNA delivery system
Source: Plant Biotechnol J. 2022 Sep 7;20(12):2332–41. doi: 10.1111/pbi.13910 (PMC9674318; doi:10.1111/pbi.13910)
Supplement: Supplementary file 1 — Figure S1 The sequences of barley stripe mosaic virus (BSMV) plasmids used in the study. Figure S2 The expression level of Cas9 in transgenic plants. Figure S3 Somatic editing induced by the BSMV‐sgRNA constructs with and without mobile RNA in the noninoculated 4th leaf of the C413 line. Figure S4 Relationship between the mutagenesis ratio in the M1 progeny of plants inoculated by BSMV‐GW2T2 (a, b, and d) or BSMV‐GW2T2 (c and e) and the somatic editing efficiency evaluated in the 6th (a), 4th (b and c), and 2nd (d and e) leaf. Figure S5 The somatic editing efficiency of each target site in the 4th leaf (developed after inoculation) of plants inoculated by multiplex editing pool (BSMV‐GUG) or BSMV carrying single guide RNAs. Figure S6 The alignment of the Q gene promoter region from the A, B, and D genomes along with the CRISPR‐Cas9 targets. Figure S7 Alignment of the Q gene (chromosome 5A) promoter from cultivars Chinese Spring and Bobwhite. Figure S8 Expression of the Q gene's A genome homoeolog in the M1 plants at the 6‐leaf stage. Figure S9 The Q‐5A gene expression level in spikes of M2 plants with edits in the Q‐5A promoter. Figure S10 Validation of the A genome‐specific primers for RT‐PCR of the Q gene. Figure S11 The somatic editing efficiency of BSMV‐QT1 and BSMV‐GW7T6 in line 3 613 474 and the somatic editing efficiency of BSMV‐GW7T6 in KS080093K‐18. Figure S12 The Cas9 plasmids used for creating transgenic plants. Table S1 The primers, oligos, and synthesized double‐strand DNA used in this study. Table S2 The target sites selected for BSMV‐sgRNA‐based editing in this study. Table S3 The efficiency of the Q gene (chromosome 5A) editing based on the BSMV‐sgRNA delivery system in transgenic wheat lines with the low (7438) and high (C413) levels of Cas9 expression. Table S4 The efficiency of somatic editing in the wheat leaves inoculated with the BSMV transcripts carrying the TaGW2 gRNA with and without mobile elements. Table S5 Somatic editing efficiency of in [file PBI-20-2332-s001.docx]

**Supplementary Information**

**Multiplexed promoter and gene editing in wheat using a virus-based guide RNA delivery system**

Wei Wang^1,2^, Zitong Yu^1,2^, Fei He^1#^, Guihua Bai^3^, Harold N. Trick^1^, Alina Akhunova^1,4^, and Eduard Akhunov^1,2*^

^1^ Department of Plant Pathology, Kansas State University, Manhattan, KS, USA; ^2^ Wheat Genetics Resource Center, Kansas State University, Manhattan, KS, USA; ^3^ USDA-ARS, Hard Winter Wheat Genetics Research Unit, Manhattan, KS; ^4^ Integrated Genomic Facility, Kansas State University, Manhattan, KS, USA.

*Corresponding author: Eduard Akhunov, [eakhunov@ksu.edu](mailto:eakhunov@ksu.edu)

pBSMVα cgttcagcccgaccgctgcgccttatccggtaactatcgtcttgagtccaacccggtaagacacgacttatcgccactggcagcagccactggtaacaggattagcagagcgaggtatgtaggcggtgctacagagttcttgaagtggtggcctaactacggctacactagaagaacagtatttggtatctgcgctctgctgaagccagttaccttcggaaaaagagttggtagctcttgatccggcaaacaaaccaccgctggtagcggtggtttttttgtttgcaagcagcagattacgcgcagaaaaaaaggatctcaagaagatcctttgatcttttctacggggtctgacgctcagtggaacgaaaactcacgttaagggattttggtcatgagattatcaaaaaggatcttcacctagatccttttaaattaaaaatgaagttttaaatcaatctaaagtatatatgagtaaacttggtctgacagttaccaatgcttaatcagtgaggcacctatctcagcgatctgtctatttcgttcatccatagttgcctgactccccgtcgtgtagataactacgatacgggagggcttaccatctggccccagtgctgcaatgataccgcgagacccacgctcaccggctccagatttatcagcaataaaccagccagccggaagggccgagcgcagaagtggtcctgcaactttatccgcctccatccagtctattaattgttgccgggaagctagagtaagtagttcgccagttaatagtttgcgcaacgttgttgccattgctacaggcatcgtggtgtcacgctcgtcgtttggtatggcttcattcagctccggttcccaacgatcaaggcgagttacatgatcccccatgttgtgcaaaaaagcggttagctccttcggtcctccgatcgttgtcagaagtaagttggccgcagtgttatcactcatggttatggcagcactgcataattctcttactgtcatgccatccgtaagatgcttttctgtgactggtgagtactcaaccaagtcattctgagaatagtgtatgcggcgaccgagttgctcttgcccggcgtcaatacgggataataccgcgccacatagcagaactttaaaagtgctcatcattggaaaacgttcttcggggcgaaaactctcaaggatcttaccgctgttgagatccagttcgatgtaacccactcgtgcacccaactgatcttcagcatcttttactttcaccagcgtttctgggtgagcaaaaacaggaaggcaaaatgccgcaaaaaagggaataagggcgacacggaaatgttgaatactcatactcttcctttttcaatattattgaagcatttatcagggttattgtctcatgagcggatacatatttgaatgtatttagaaaaataaacaaataggggttccgcgcacatttccccgaaaagtgccacctaaattgtaagcgttaatattttgttaaaattcgcgttaaatttttgttaaatcagctcattttttaaccaataggccgaaatcggcaaaatcccttataaatcaaaagaatagaccgagatagggttgagtgttgttccagtttggaacaagagtccactattaaagaacgtggactccaacgtcaaagggcgaaaaaccgtctatcagggcgatggcccactacgtgaaccatcaccctaatcaagttttttggggtcgaggtgccgtaaagcactaaatcggaaccctaaagggagcccccgatttagagcttgacggggaaagccggcgaacgtggcgagaaaggaagggaagaaagcgaaaggagcgggcgctagggcgctggcaagtgtagcggtcacgctgcgcgtaaccaccacacccgccgcgcttaatgcgccgctacagggcgcgtcccattcgccattcaggctgcgcaactgttgggaagggcgatcggtgcgggcctcttcgctattacgccagctggcgaaagggggatgtgctgcaaggcgattaagttgggtaacgccagggttttcccagtcacgacgttgtaaaacgacggccagtgaattaatacgactcactataGTATGTAAGTTGCCTTTGGGTGTAAAATTTCTTGCATGCACATAATCGTAATCGATTCTTCTTGATCTCTAAACAACACTTTCCCGTTAGCATGGCTAGCGATGAGATTGTCCGCAATCTGATCTCCCGTGAGGAGGTGATGGGTAATTTGATTAGCACAGCTTCTAGCTCAGTAAGGTCACCCTTACATGACGTACTGTGCTCGCACGTAAGGACCATCGTCGATTCCGTGGATAAGAAAGCGGTCAGTCGCAAGCATGTTGATGTACGGCGCAACATCTCCTCTGAAGAGTTACAGATGTTGATAAATGCATATCCTGAATATGCCGTTTCATCCTCAGCTTGTGAATCTGGTACTCATAGCATGGCGGCTTGTTTTCGATTTCTGGAGACAGAATACCTCTTAGATATGGTTCCAATGAAAGAGACTTTTGTTTATGACATTGGTGGTAACTGGTTTTCTCATATGAAGTTTCGTGCTGATAGAGAAATTCATTGTTGCTGTCCGATCTTATCTATGAGAGATTCTGAAAGACTGGAAACACGCATGATGGCAATGCAAAAATATATGCGTGGATCGAAAGACAAACCGTTACGCTTGTTAAGCCGTTATCAAAATATCCTGCGTGAACAAGCGGCGAGAACAACTGCCTTTATGGCAGGTGAGGTGAATGCGGGTGTTCTCGATGGAGATGTGTTTTGTGAGAACACTTTTCAAGACTGTGTGAGACAGGTGCCCGAAGGTTTTTTGAAGACAGCTATAGCAGTTCATAGCATCTACGATATCAAAGTGGAAGAATTTGCGTCTGCATTGAAAAGAAAAGGTATAACACAGGCTTATGGGTGCTTCCTGTTTCCTCCTGCTGTATTGATAGGTCAGAAGGAAGGTATTTTACCTTCCGTGGACGGTCATTACTTGGTGGAGAATGGCAGGATTAAGTTCTTCTTTGCGAATGATCCGAATGCCGGTTACTCTCATGACCTTAAGGATTATCTGAAGTATGTGGAAAAAACCTACGTGGATATAAAGGATGGAGTGTTTGCTATTGAGCTGATGCAAATGCGAGGTGATACCATGTTCTTTAAGATCACGGATGTCACCGCAGCAATGTATCATATGAAATACAGAGGTATGAAACGTGATGAAACATTCAAATGCATTCCGTTGCTAAAAAATTCATCCGTTGTCGTACCTCTATTTTCGTGGGACAACCGTTCTTTAAAGATCACAAGTGGTTTATTACCACGAACTTTGGTCGAGCAAGGTGCGGCGTTTATTATGAAAAACAAGGAGAAGGACTTGAACGTTGCTGTGTTGAAGAACTATCTTTCCGCTGTGAACAACTCATACATTTTCAACGGATCCCAGGTTAGAGATGGTGTGAAAATTGCCCCGGATTTAATCTCCAAATTGGCAGTGACTCTGTACCTGAGAGAAAAGGTCTATCGACAAAGAGAAAATTCAATCATAAGTTATTTCGAGCAAGAAATGCTTCACGATCCCAACTTGAAAGCCATGTTTGGAGACTTTCTGTGGTTTGTTCCAAATACTCTCTCGAGTGTCTGGAAGAACATGCGAAAATCACTGATGGAATGGTTTGGCTACGCAGAATTTGACTTGACTACTTTTGATATTTGCGATCCCGTTCTCTACGTAGAGATAGTGGATCGGTATAAGATCATTCAAAAAGGGCGAATTCCACTTGGTGAGTTTTTTGATTGTCATGAAGAATGCGAGAATTACGAACTGCGTGAGAAGGAGAAAAATGACCTAGCGGTGAAAATGGCCCAGAAGGTAACAGGGACGGTGACCGAATGCGAGAAGGACCTGGGACCTCTTGTTCAACCGATAAAACAGATATTGGTTCAACTTGTGATGCCCAATTTGGTCAGAGCGCTGTGTAGACCTCGTAGCCCAACGTCTCCTTTGGACTTAAATATCCCAGGGTCAACTCCATCACACTCAAGTTCAGATTCTGAACAATCTATGACTGAAGAAGCGAGCTGCGCCATTGCGGGTAGCGTACCAACATGGGAAATTGCGACTAAGAAAGATCTAACCTTTCAGCGAATTGATGAAGATATGTCTCGACGAACTGGTATGCCTCCAAGACCAAAAGTAACTTCTAGTTACAACATGAATGCCAGAGCTGAGTTTCTCTACTATCAACTGTGTAGCGTGATTTGTGAAAGGGCTCAGATTTTGAGTGTCATCGAAGACTTTCGTCAGAATTTGATATTCTCAGATAAAGTGGCCGTTCCATTGAACGCTAGATTCTACAGTTTTCAGTCATTGCAACCCGGATGGGTGTTCAAGACTCCATCGCATAGTGAAGTAGGCCACAGTTATGCAGTACATTTTGACTTCAAGACAGTTGGAACCGATTTGGAAGAGAGCCTAGCTTTTTGCCGAATGGTACCGATTTCATGGGATAAAAGCGGCAAATACATCGCGACAACTCCTCATTTTCCCGAGAGACATGGTTACTACGTGATTTGTGACAACACTAAATTGTGTAACAATTGGCTTATTTACAATAAGTTAGTTGATGTCTACGCACGAGTGGCTGATAGACCTCTGAGATTTGAGTTGATTGACGGAGTTCCTGGCTGCGGAAAGTCAACCATGATTTTAAACAGCTGTGATATTCGACGCGAAGTTGTTGTTGGTGAAGGAAGGAATGCAACTGATGACTTAAGGGAGAGGTTCAAGCGTAAGAAAAATTTGAATAGTAAGACTGCTAATCATAGAGTTCGAACGCTTGACAGCTTATTACTTGCTGAAGGACCTTGTGTACCGCAAGCTGATAGGTTTCATTTTGATGAAGCTCTAAAAGTTCATTACGGCGCCATAATGTTCTGTGCTGATAAGCTTGGTGCCTCAGAAATTCTCGCTCAGGGAGATAGGGCTCAACTGCCGATGATCTGTCGTGTAGAAGGTATTGAACTTCAATTTCAATCTCCTGATTACACGAAGACGATCATAAATCCTAAGCTACGATCATACCGTATCCCTGGAGATGTTGCCTTCTATTTGAGTGCTAAGGAATTTTACAAAGTTAAAGGAATACCTCAAAAGGTTACAACTTCTAACAGTGTGAAACGTTCCCTGTACGCTAGAGGCGAAACAACTCCGGAAAGATTCGTGAGTTTGCTTGATGTTCCGGTGAGAAAAAACACCCATTATCTAACCTTCTTACAAGCTGAGAAGGAAAGTTTGATGAGTCATTTGATTCCAAAGGGTGTGAAGAAAGAGTCTATTTCAACGATTCATGAGGCGCAGGGTGGTACCTATGAAAATGTGATTCTGGTCCGTTTGCAACGGACGCCCAATGAAATTTATCCGGGTGGACCTAGGTCCGCCCCTTACATTGTGGTTGGGACTTCAAGGCATACAAAAACTTTCACTTATTGTAGTGTTACGGACGATAAGTTGCTTTTAGATATCGCCGACGTCGGTGGTATTGCACATACACCTATTCGTACTTTTGAATCTCATATAGTTTAAAAAAAAAAAAAAAAAAAAAAAAAAAAAAAAAAAAAAAAATGTTTGATCAGATCATTCAAATCTGATGGTGCCCATCAACCATATGATGGGAGTGTTTGCAAGTCCACTATAATCGAACTTGAAAACGATGCCTGAATTGGAAACCATGAATCTTAACGGATTCTGGAGAGAAAATTTAGGAATTGGTATGTAAGCTACAACTTCCGGTAGCTGCGTCACACTTTAAGAGTGTGCATACTGAGCCGAAGCTCAGCTTCGGTCCCCCAAGGGAAGACCAcgcgtcatgcaagcttggcgtaatcatggtcatagctgtttcctgtgtgaaattgttatccgctcacaattccacacaacatacgagccggaagcataaagtgtaaagcctggggtgcctaatgagtgagctaactcacattaattgcgttgcgctcactgcccgctttccagtcgggaaacctgtcgtgccagctgcattaatgaatcggccaacgcgcggggagaggcggtttgcgtattgggcgctcttccgcttcctcgctcactgactcgctgcgctcggtcgttcggctgcggcgagcggtatcagctcactcaaaggcggtaatacggttatccacagaatcaggggataacgcaggaaagaacatgtgagcaaaaggccagcaaaaggccaggaaccgtaaaaaggccgcgttgctggcgtttttccataggctccgcccccctgacgagcatcacaaaaatcgacgctcaagtcagaggtggcgaaacccgacaggactataaagataccaggcgtttccccctggaagctccctcgtgcgctctcctgttccgaccctgccgcttaccggatacctgtccgcctttctcccttcgggaagcgtggcgctttctcatagctcacgctgtaggtatctcagttcggtgtaggtcgttcgctccaagctgggctgtgtgcacgaaccccccgttcagcccgaccgctgcgccttatccggtaactatcgtcttgagtccaac

pBSMVβ

cgttcagcccgaccgctgcgccttatccggtaactatcgtcttgagtccaacccggtaagacacgacttatcgccactggcagcagccactggtaacaggattagcagagcgaggtatgtaggcggtgctacagagttcttgaagtggtggcctaactacggctacactagaagaacagtatttggtatctgcgctctgctgaagccagttaccttcggaaaaagagttggtagctcttgatccggcaaacaaaccaccgctggtagcggtggtttttttgtttgcaagcagcagattacgcgcagaaaaaaaggatctcaagaagatcctttgatcttttctacggggtctgacgctcagtggaacgaaaactcacgttaagggattttggtcatgagattatcaaaaaggatcttcacctagatccttttaaattaaaaatgaagttttaaatcaatctaaagtatatatgagtaaacttggtctgacagttaccaatgcttaatcagtgaggcacctatctcagcgatctgtctatttcgttcatccatagttgcctgactccccgtcgtgtagataactacgatacgggagggcttaccatctggccccagtgctgcaatgataccgcgagacccacgctcaccggctccagatttatcagcaataaaccagccagccggaagggccgagcgcagaagtggtcctgcaactttatccgcctccatccagtctattaattgttgccgggaagctagagtaagtagttcgccagttaatagtttgcgcaacgttgttgccattgctacaggcatcgtggtgtcacgctcgtcgtttggtatggcttcattcagctccggttcccaacgatcaaggcgagttacatgatcccccatgttgtgcaaaaaagcggttagctccttcggtcctccgatcgttgtcagaagtaagttggccgcagtgttatcactcatggttatggcagcactgcataattctcttactgtcatgccatccgtaagatgcttttctgtgactggtgagtactcaaccaagtcattctgagaatagtgtatgcggcgaccgagttgctcttgcccggcgtcaatacgggataataccgcgccacatagcagaactttaaaagtgctcatcattggaaaacgttcttcggggcgaaaactctcaaggatcttaccgctgttgagatccagttcgatgtaacccactcgtgcacccaactgatcttcagcatcttttactttcaccagcgtttctgggtgagcaaaaacaggaaggcaaaatgccgcaaaaaagggaataagggcgacacggaaatgttgaatactcatactcttcctttttcaatattattgaagcatttatcagggttattgtctcatgagcggatacatatttgaatgtatttagaaaaataaacaaataggggttccgcgcacatttccccgaaaagtgccacctaaattgtaagcgttaatattttgttaaaattcgcgttaaatttttgttaaatcagctcattttttaaccaataggccgaaatcggcaaaatcccttataaatcaaaagaatagaccgagatagggttgagtgttgttccagtttggaacaagagtccactattaaagaacgtggactccaacgtcaaagggcgaaaaaccgtctatcagggcgatggcccactacgtgaaccatcaccctaatcaagttttttggggtcgaggtgccgtaaagcactaaatcggaaccctaaagggagcccccgatttagagcttgacggggaaagccggcgaacgtggcgagaaaggaagggaagaaagcgaaaggagcgggcgctagggcgctggcaagtgtagcggtcacgctgcgcgtaaccaccacacccgccgcgcttaatgcgccgctacagggcgcgtcccattcgccattcaggctgcgcaactgttgggaagggcgatcggtgcgggcctcttcgctattacgccagctggcgaaagggggatgtgctgcaaggcgattaagttgggtaacgccagggttttcccagtcacgacgttgtaaaacgacggccagtgaattaatacgactcactataGTAAAAGAAAAGGAACAACCCTGTTGTTGTTCGACGCTATACTAAATATATATTATCTTATTAGTGCATTTCTTTTACCACTTCACAGTATGCCGAACGTTTCTTTGACTGCTAAGGGTGGAGGACACTACAACGAGGATCAATGGGATACACAAGTTGTGGAAGCCGGAGTATTTGACGATTGGTGGGTCCACGTAGAAGCCTGGAATAAATTTCTAGACAATTTACGTGGTATCAACTTTAGCGTTGCTTCCTCTCGGTCGCAAGTCGCTGAATGCTTAGCTGCGTTAGATCGTGATCTACCTGCTGATGTAGACAGACGGTTTGCAGGTGCTAGAGGACAAATTGGTTTACCCAATTATCTTCCTGCGCCAAAATTCTTTCGTCTCGATAAGCGAACTATCGCTGAACTGACTAGACTCTCTCGTCTTACGGATCAGCCGCACAACAATCGTGATATAGAGCTTAACCGAGCGAAAAGAGCCACAACTAACCCATCTCCCCCGGCGCAGGCACCGTCGGAGAATCTTACTCTTCGTGATGTTCAACCGTTAAAGGATAGTGCGTTGCATTATCAATACGTGTTGATTGACCTACAGAGTGCGAGACTCCCAGTGTATACCAGGAAGACTTTCGAACGTGAACTCGCTTTGGAATGGATCATTCCAGATGCCGAGGAAGCGTGACCTGCTGTTGAAGCGGTAAAAGGATGTACATATGTATCTTATTTATTTTGTTTATCTATTTTCTTTTACTTTTAGTTTTTGCTTTTTACGCGTTAACTAGATGTATTGACTTTAGCCATGGACATGACGAAAACTGTTGAGGAAAAGAAAACAAATGGAACTGATTCAGTGAAAGGTGTTTTTGAAAACTCGACGATTCCCAAAGTTCCGACTGGACAGGAAATGGGTGGTGACGATTCTTCTACTTCTAAATTAAAGGAAACTCTAAAAGTTGCCGATCAGACTCCATTGTCCGTTGACAACGGTGCCAAATCCAAATTGGATTCTTCTGATAGACAAGTTCCTGGTCCTAAGTTGGCAACAACTGTGGAAAAGGAACCTGAGTTGAAACCCAACGTTAAGAAGTCCAAGAAGAAAAGAATCCAAAAACCTGCTCAACCGAGTAGGCCCAATGACCTTAAAGGCGGGACTAAGGGATCATCTCAAGTGGGTGAAAATGTGAGTGAGAACTATACTGGGATTTCTAAGGAAGCAGCTAAGCAAAAGCAGAAGACACCCAAGTCTGTGAAAATGCAAAGCAATCTGGCCGATAAGTTCAAAGCGAATGATACTCGTAGATCGGAATTAATTAACAAGTTTCAGCAATTTGTGCATGAAACCTGTCTTAAATCTGATTTTGAGTACACTGGTCGACAGTATTTCAGAGCTAGATCAAATTTCTTTGAAATGATTAAGCTCGCATCCTTGTATGACAAACATCTAAAGGAATGTATGGCGCGAGCCTGCACCCTAGAACGAGAACGATTGAAGCGTAAGTTACTCCTAGTACGAGCTTTGAAACCAGCAGTTGACTTCCTTACGGGAATCATCTCTGGAGTTCCTGGCTCAGGAAAATCAACCATTGTGCGTACTTTGCTCAAAGGTGAATTTCCGGCTGTTTGTGCTTTGGCCAATCCTGCCTTAATGAACGACTATTCTGGTATTGAAGGCGTTTACGGGTTAGATGACCTGTTGCTTTCTGCAGTTCCGATAACGTCTGATTTATTGATCATAGATGAATATACACTTGCTGAGAGCGCGGAAATCCTGTTGTTACAACGAAGACTCAGAGCCTCTATGGTGTTGTTAGTCGGGGATGTAGCTCAAGGAAAAGCCACCACTGCTTCCAGTATTGAGTATTTAACTCTGCCGGTGATCTACAGATCAGAGACGACTTATCGTTTGGGACAAGAGACTGCTTCGCTTTGCAGCAAGCAGGGTAACAGAATGGTTTCAAAGGGTGGAAGGGACACAGTGATCATTACTGATTACGATGGCGAAACAGATGGAACGGAGAAAAATATCGCTTTTACTGTCGATACAGTTCGAGATGTGAAAGATTGCGGGTACGATTGTGCCCTGGCAATTGATGTGCAAGGGAAAGAATTCGATTCAGTGACTTTATTCCTAAGGAACGAAGACCGGAAAGCTTTAGCAGATAAGCATTTGCGTTTAGTCGCTTTGAGCAGACATAAGTCGAAGTTAATCATCAGGGCCGACGCGGAAATTCGTCAAGCATTCCTGACAGGTGATATTGACTTGAGCTCTAAGGCGAGTAACTCTCATCGTTATTCTGCAAAACCGGATGAAGACCACAGTTGGTTCAAGGCCAAATAAGTATTGGCCAATTGTCGCCGGAATCGGTGTCGTTGGATTGTTTGCGTATTTGATCTTTTCAAATCAAAAACATTCTACGGAATCCGGCGATAATATTCACAAATTCGCCAACGGAGGTAGTTACAGGGACGGGTCAAAGAGTATAAGTTATAATCGTAATCATCCTTTTGCCTATGGCAATGCCTCATCCCCTGGAATGTTGTTGCCCGCAATGCTTACCATCATCGGAATCATTTCCTATTTATGGCGAACAAGAGATTCCGTGCTCGGAGACTCAGGCGGAAACAATTCCTGCGGAGAAGACTGTCAGGGCGAATGTCTTAACGGACATTCTCGACGATCATTACTATGCGATATTGGCTAGTCTTTTTATCATTGCTCTATGGTTATTGTATATATATCTAAGCAGTATACCTACGGAGACTGGTCCCTACTTCTATCAAGATCTGAACTCTGTGAAGATCTATGGAATAGGGGCTACGAATCCAGAAGTTATTGCGGCCATCCACCATTGGCAGAAGTACCCTTTTGGGGAATCTCCGATGTGGGGAGGTTTAGTCAGTGTTTTGAGCGTTCTTCTTAAACCGCTGACGTTAGTTTTTGCGTTAAGCTTTTTTCTCTTACTTTCTTCAAAAAGGTAAAAAAAAAAAAAAAAAAAATGTTTGATCAGATCATTCAAATCTGATGGTGCCCATCAACCATATGATGGGAGTGTTTGCAAGTCCACTATAATCGAACTTGAAAACGATGCCTGAATTGGAAACCATGAATCTTAACGGATTCTGGAGAGAAAATTTAGGAATTGGTATGTAAGCTACAACTTCCGGTAGCTGCGTCACACTTTAAGAGTGTGCATACTGAGCCGAAGCTCAGCTTCGGTCCCCCAAGGGAAGACCActagtcatgcaagcttggcgtaatcatggtcatagctgtttcctgtgtgaaattgttatccgctcacaattccacacaacatacgagccggaagcataaagtgtaaagcctggggtgcctaatgagtgagctaactcacattaattgcgttgcgctcactgcccgctttccagtcgggaaacctgtcgtgccagctgcattaatgaatcggccaacgcgcggggagaggcggtttgcgtattgggcgctcttccgcttcctcgctcactgactcgctgcgctcggtcgttcggctgcggcgagcggtatcagctcactcaaaggcggtaatacggttatccacagaatcaggggataacgcaggaaagaacatgtgagcaaaaggccagcaaaaggccaggaaccgtaaaaaggccgcgttgctggcgtttttccataggctccgcccccctgacgagcatcacaaaaatcgacgctcaagtcagaggtggcgaaacccgacaggactataaagataccaggcgtttccccctggaagctccctcgtgcgctctcctgttccgaccctgccgcttaccggatacctgtccgcctttctcccttcgggaagcgtggcgctttctcatagctcacgctgtaggtatctcagttcggtgtaggtcgttcgctccaagctgggctgtgtgcacgaaccccccgttcagcccgaccgctgcgccttatccggtaactatcgtcttgagtccaac

pBSMVγ

cgttcagcccgaccgctgcgccttatccggtaactatcgtcttgagtccaacccggtaagacacgacttatcgccactggcagcagccactggtaacaggattagcagagcgaggtatgtaggcggtgctacagagttcttgaagtggtggcctaactacggctacactagaagaacagtatttggtatctgcgctctgctgaagccagttaccttcggaaaaagagttggtagctcttgatccggcaaacaaaccaccgctggtagcggtggtttttttgtttgcaagcagcagattacgcgcagaaaaaaaggatctcaagaagatcctttgatcttttctacggggtctgacgctcagtggaacgaaaactcacgttaagggattttggtcatgagattatcaaaaaggatcttcacctagatccttttaaattaaaaatgaagttttaaatcaatctaaagtatatatgagtaaacttggtctgacagttaccaatgcttaatcagtgaggcacctatctcagcgatctgtctatttcgttcatccatagttgcctgactccccgtcgtgtagataactacgatacgggagggcttaccatctggccccagtgctgcaatgataccgcgagacccacgctcaccggctccagatttatcagcaataaaccagccagccggaagggccgagcgcagaagtggtcctgcaactttatccgcctccatccagtctattaattgttgccgggaagctagagtaagtagttcgccagttaatagtttgcgcaacgttgttgccattgctacaggcatcgtggtgtcacgctcgtcgtttggtatggcttcattcagctccggttcccaacgatcaaggcgagttacatgatcccccatgttgtgcaaaaaagcggttagctccttcggtcctccgatcgttgtcagaagtaagttggccgcagtgttatcactcatggttatggcagcactgcataattctcttactgtcatgccatccgtaagatgcttttctgtgactggtgagtactcaaccaagtcattctgagaatagtgtatgcggcgaccgagttgctcttgcccggcgtcaatacgggataataccgcgccacatagcagaactttaaaagtgctcatcattggaaaacgttcttcggggcgaaaactctcaaggatcttaccgctgttgagatccagttcgatgtaacccactcgtgcacccaactgatcttcagcatcttttactttcaccagcgtttctgggtgagcaaaaacaggaaggcaaaatgccgcaaaaaagggaataagggcgacacggaaatgttgaatactcatactcttcctttttcaatattattgaagcatttatcagggttattgtctcatgagcggatacatatttgaatgtatttagaaaaataaacaaataggggttccgcgcacatttccccgaaaagtgccacctaaattgtaagcgttaatattttgttaaaattcgcgttaaatttttgttaaatcagctcattttttaaccaataggccgaaatcggcaaaatcccttataaatcaaaagaatagaccgagatagggttgagtgttgttccagtttggaacaagagtccactattaaagaacgtggactccaacgtcaaagggcgaaaaaccgtctatcagggcgatggcccactacgtgaaccatcaccctaatcaagttttttggggtcgaggtgccgtaaagcactaaatcggaaccctaaagggagcccccgatttagagcttgacggggaaagccggcgaacgtggcgagaaaggaagggaagaaagcgaaaggagcgggcgctagggcgctggcaagtgtagcggtcacgctgcgcgtaaccaccacacccgccgcgcttaatgcgccgctacagggcgcgtcccattcgccattcaggctgcgcaactgttgggaagggcgatcggtgcgggcctcttcgctattacgccagctggcgaaagggggatgtgctgcaaggcgattaagttgggtaacgccagggttttcccagtcacgacgttgtaaaacgacggccagtgaattaatacgactcactataGTATAGCTTGAGCATTACCGTCGTGTAATTGCAACACTTGGCTTGCCAAATAACGCTAAAGCGTTCACGAAACAAACAACACTTCGGCATGGATGTTGTGAAGAAATTCGCCGTCATGTCAGTGACTGTAGTAGCAGGTCCCGTCCTTACGCTTTCATCACCTGTGGTGGTGACGTTTGGAACAGGCTTAATTGCCGTATCTTTGGTGAAACGGTTGCTACAGGAACAACCCCGTGTAATTGCTCACGATCACGAACATTACCCAGGTGGTTCTGAGAGCAGTTCTAGCTCTTGTGCTACCGCGCCTATTTTACGTAATCTTTCGCGAGATCAGTGCGATTCAGAGAATATTGGATGCAGTTCTAGCGCCTGTTCTCCGTCTGAAATTGTGAAAGTTACAAGGCAGGTAGTGGGAGTTGAACGTGGTCTTTACCGGGACATTTTTCAGGACAACGAAATCCCATCAGTCATGGAAGAGAAACTGCAGAAACTCCTTTACTCTGAGGGTGAGAAGATTCGAAGACGTTGCCAATTTGAAGCATCAACGATGCACTCACGCAAAGTAAAGGTTCCGGAGGTAGGTACTATCCCAGATATCCAAACTTGGTTCGATGCTACGTTTCCTGGTAACTCCGTTAGGTTTTCTGATTTCGACGGTTATACTGTTGCTACGGAGGACATTAACATGGATGTTCAGGATTGTAGACTTAAGTTCGGGAAGACTTTTCGACCTTATGAATTTAAGGAATCACTGAAACCAGTACTGAGGACAGCAATGCCAGAAAAACGACAGGGTAGTTTGATTGAAAGTGTGCTGGCCTTTCGTAAAAGAAATTTGGCTGCGCCCAGATTACAAGGAGCTTTGAATGAATGGCACACAATTGAGAATGTGCTAACGAAGGCGTTAAAGGTATTCTTCTTTGAAGATTTAATTGATCGAACGGATCACTGCACTTACGAGTCAGCGCTCAGATGGTGGGATAAACAATCAGTGACAGCTCGAGCGCAGCTCGTGGCGGATCAGCGGAGGTTATGTGATGTTGACTTCACGACTTATAACTTCATGATAAAAAATGATGTAAAGCCGAAGTTAGATCTAACACCTCAAGTTGAATATGCAGCTTTGCAGACTGTTGTATATCCTGATAAGATAGTCAATGCTTTCTTTGGTCCGATCATAAAGGAGATTAATGAACGGATCATCAGAGCGCTTAGACCTCATGTGGTCTTTAATTCTCGTATGACTGCTGATGAACTGAATGAAACAGCTGCCTTTTTGACACCTCATAAGTACAGAGCCTTAGAGATTGATTTTTCAAAATTTGATAAATCAAAGACTGGGCTTCATATCAAAGCTGTCATTGGACTCTATAAGCTCTTTGGCCTAGATGGCCTGTTAAAAGTGCTCTGGGAAAAATCGCAATATCAGACTTACGTGAAAGATAGAAACTTCGGTCTCGAGGCATATCTATTGTATCAGCAAAAGTCAGGAAATTGTGACACTTACGGTTCGAACACCTGGTCTGCCGCCTTGGCGTTGTTAGATTGTCTTCCTTTGGAAGATGCACATTTCTGTGTATTTGGTGGTGATGATTCATTGATATTGTTTGATCAGGGATACATAATTTCCGACCCATGCCGGCAACTTGCCGGTACTTGGAATCTTGAATGTAAAGTGTTCGACTTCAAGTACCCCGCATTTTGTGGTAAATTTCTGCTGTGCATAGATGGAAAATATCAATTTGTTCCAGATGCGGCAAAATTTATCACAAAATTAGGTAGAACTGATGTGAGAGATGTAGAAGTTTTGAGTGAGATTTATATCTCTATCAATGACAATTACAAATCTTACAAAGACTTTAAGGTGCTTGATGCTTTGGATAAGGCTTTAGTGGATAGATATCGATCCCCTTATAGTGCTATTTCTGCTTTGGTTTCTTTATGTTATCATATCTTTGACTTTAATAAGTTTAAGTTGCTGTTTAATTGTGAAGGGAAATTTGTGGATAAGAAGCTGAGAAAAGACTTCGAGTGGTGAACTCTAGGTCCTGATGTTTAAATCTACTGTATTTACCTTCGCATGATGGCTACTTTCTCTTGTGTGTGTTGTGGTACCTTAACTACAAGTACTTACTGTGGTAAGAGATGTGAGCGAAAGCATGTATATTCTGAAACAAGAAATAAGAGATTGGAACTTTACAAGAAGTATCTATTGGAACCGCAAAAATGCGCCCTGAATGGAATCGTTGGACACAGTTGTGGAATGCCATGCTCCATTGCGGAAGAGGCTTGTGATCAACTGCCAATCGTGAGTAGGTTCTGTGGCCAAAAGCATGCGGATCTGTATGATTCACTTCTGAAACGTTCTGAACAGGAGTTACTTCTTGAATTTCTCCAGAAGAAGATGCAGGAGCTGAAACTTTCTCATATCGTAAAAATGGCTAAGCTTGAAAGTGAGGTTAACGCAATACGTAAGTCCGTAGCTTCTTCTTTTGAAGATTCTGTTGGATGTGATGATTCTTCTTCCGTTTCTAAGTAAAAAAAAAAAAAAATGTTTGATCAGATCATTCAAATCTGATGGTGCCCATCAACCATATGATGGGAGTGTTTGCAAGTCCACTATAATCGAACTTGAAAACGATGCCTGAATTGGAAACCATGAATCTTAACGGACTCTGGAGAGAAAATTTAGGAATTGGTATGTAAGCTACAACTTCCGGTAGCTGCGTCACACTTTAAGAGTGTGCATACTGAGCCGAAGCTCAGCTTCGGTCCCCCAAGGGAAGACCAcgcgtcatgcaagcttggcgtaatcatggtcatagctgtttcctgtgtgaaattgttatccgctcacaattccacacaacatacgagccggaagcataaagtgtaaagcctggggtgcctaatgagtgagctaactcacattaattgcgttgcgctcactgcccgctttccagtcgggaaacctgtcgtgccagctgcattaatgaatcggccaacgcgcggggagaggcggtttgcgtattgggcgctcttccgcttcctcgctcactgactcgctgcgctcggtcgttcggctgcggcgagcggtatcagctcactcaaaggcggtaatacggttatccacagaatcaggggataacgcaggaaagaacatgtgagcaaaaggccagcaaaaggccaggaaccgtaaaaaggccgcgttgctggcgtttttccataggctccgcccccctgacgagcatcacaaaaatcgacgctcaagtcagaggtggcgaaacccgacaggactataaagataccaggcgtttccccctggaagctccctcgtgcgctctcctgttccgaccctgccgcttaccggatacctgtccgcctttctcccttcgggaagcgtggcgctttctcatagctcacgctgtaggtatctcagttcggtgtaggtcgttcgctccaagctgggctgtgtgcacgaaccccccgttcagcccgaccgctgcgccttatccggtaactatcgtcttgagtccaac

pBSMVγPDS

cgttcagcccgaccgctgcgccttatccggtaactatcgtcttgagtccaacccggtaagacacgacttatcgccactggcagcagccactggtaacaggattagcagagcgaggtatgtaggcggtgctacagagttcttgaagtggtggcctaactacggctacactagaagaacagtatttggtatctgcgctctgctgaagccagttaccttcggaaaaagagttggtagctcttgatccggcaaacaaaccaccgctggtagcggtggtttttttgtttgcaagcagcagattacgcgcagaaaaaaaggatctcaagaagatcctttgatcttttctacggggtctgacgctcagtggaacgaaaactcacgttaagggattttggtcatgagattatcaaaaaggatcttcacctagatccttttaaattaaaaatgaagttttaaatcaatctaaagtatatatgagtaaacttggtctgacagttaccaatgcttaatcagtgaggcacctatctcagcgatctgtctatttcgttcatccatagttgcctgactccccgtcgtgtagataactacgatacgggagggcttaccatctggccccagtgctgcaatgataccgcgagacccacgctcaccggctccagatttatcagcaataaaccagccagccggaagggccgagcgcagaagtggtcctgcaactttatccgcctccatccagtctattaattgttgccgggaagctagagtaagtagttcgccagttaatagtttgcgcaacgttgttgccattgctacaggcatcgtggtgtcacgctcgtcgtttggtatggcttcattcagctccggttcccaacgatcaaggcgagttacatgatcccccatgttgtgcaaaaaagcggttagctccttcggtcctccgatcgttgtcagaagtaagttggccgcagtgttatcactcatggttatggcagcactgcataattctcttactgtcatgccatccgtaagatgcttttctgtgactggtgagtactcaaccaagtcattctgagaatagtgtatgcggcgaccgagttgctcttgcccggcgtcaatacgggataataccgcgccacatagcagaactttaaaagtgctcatcattggaaaacgttcttcggggcgaaaactctcaaggatcttaccgctgttgagatccagttcgatgtaacccactcgtgcacccaactgatcttcagcatcttttactttcaccagcgtttctgggtgagcaaaaacaggaaggcaaaatgccgcaaaaaagggaataagggcgacacggaaatgttgaatactcatactcttcctttttcaatattattgaagcatttatcagggttattgtctcatgagcggatacatatttgaatgtatttagaaaaataaacaaataggggttccgcgcacatttccccgaaaagtgccacctaaattgtaagcgttaatattttgttaaaattcgcgttaaatttttgttaaatcagctcattttttaaccaataggccgaaatcggcaaaatcccttataaatcaaaagaatagaccgagatagggttgagtgttgttccagtttggaacaagagtccactattaaagaacgtggactccaacgtcaaagggcgaaaaaccgtctatcagggcgatggcccactacgtgaaccatcaccctaatcaagttttttggggtcgaggtgccgtaaagcactaaatcggaaccctaaagggagcccccgatttagagcttgacggggaaagccggcgaacgtggcgagaaaggaagggaagaaagcgaaaggagcgggcgctagggcgctggcaagtgtagcggtcacgctgcgcgtaaccaccacacccgccgcgcttaatgcgccgctacagggcgcgtcccattcgccattcaggctgcgcaactgttgggaagggcgatcggtgcgggcctcttcgctattacgccagctggcgaaagggggatgtgctgcaaggcgattaagttgggtaacgccagggttttcccagtcacgacgttgtaaaacgacggccagtgaattaatacgactcactataGTATAGCTTGAGCATTACCGTCGTGTAATTGCAACACTTGGCTTGCCAAATAACGCTAAAGCGTTCACGAAACAAACAACACTTCGGCATGGATGTTGTGAAGAAATTCGCCGTCATGTCAGTGACTGTAGTAGCAGGTCCCGTCCTTACGCTTTCATCACCTGTGGTGGTGACGTTTGGAACAGGCTTAATTGCCGTATCTTTGGTGAAACGGTTGCTACAGGAACAACCCCGTGTAATTGCTCACGATCACGAACATTACCCAGGTGGTTCTGAGAGCAGTTCTAGCTCTTGTGCTACCGCGCCTATTTTACGTAATCTTTCGCGAGATCAGTGCGATTCAGAGAATATTGGATGCAGTTCTAGCGCCTGTTCTCCGTCTGAAATTGTGAAAGTTACAAGGCAGGTAGTGGGAGTTGAACGTGGTCTTTACCGGGACATTTTTCAGGACAACGAAATCCCATCAGTCATGGAAGAGAAACTGCAGAAACTCCTTTACTCTGAGGGTGAGAAGATTCGAAGACGTTGCCAATTTGAAGCATCAACGATGCACTCACGCAAAGTAAAGGTTCCGGAGGTAGGTACTATCCCAGATATCCAAACTTGGTTCGATGCTACGTTTCCTGGTAACTCCGTTAGGTTTTCTGATTTCGACGGTTATACTGTTGCTACGGAGGACATTAACATGGATGTTCAGGATTGTAGACTTAAGTTCGGGAAGACTTTTCGACCTTATGAATTTAAGGAATCACTGAAACCAGTACTGAGGACAGCAATGCCAGAAAAACGACAGGGTAGTTTGATTGAAAGTGTGCTGGCCTTTCGTAAAAGAAATTTGGCTGCGCCCAGATTACAAGGAGCTTTGAATGAATGGCACACAATTGAGAATGTGCTAACGAAGGCGTTAAAGGTATTCTTCTTTGAAGATTTAATTGATCGAACGGATCACTGCACTTACGAGTCAGCGCTCAGATGGTGGGATAAACAATCAGTGACAGCTCGAGCGCAGCTCGTGGCGGATCAGCGGAGGTTATGTGATGTTGACTTCACGACTTATAACTTCATGATAAAAAATGATGTAAAGCCGAAGTTAGATCTAACACCTCAAGTTGAATATGCAGCTTTGCAGACTGTTGTATATCCTGATAAGATAGTCAATGCTTTCTTTGGTCCGATCATAAAGGAGATTAATGAACGGATCATCAGAGCGCTTAGACCTCATGTGGTCTTTAATTCTCGTATGACTGCTGATGAACTGAATGAAACAGCTGCCTTTTTGACACCTCATAAGTACAGAGCCTTAGAGATTGATTTTTCAAAATTTGATAAATCAAAGACTGGGCTTCATATCAAAGCTGTCATTGGACTCTATAAGCTCTTTGGCCTAGATGGCCTGTTAAAAGTGCTCTGGGAAAAATCGCAATATCAGACTTACGTGAAAGATAGAAACTTCGGTCTCGAGGCATATCTATTGTATCAGCAAAAGTCAGGAAATTGTGACACTTACGGTTCGAACACCTGGTCTGCCGCCTTGGCGTTGTTAGATTGTCTTCCTTTGGAAGATGCACATTTCTGTGTATTTGGTGGTGATGATTCATTGATATTGTTTGATCAGGGATACATAATTTCCGACCCATGCCGGCAACTTGCCGGTACTTGGAATCTTGAATGTAAAGTGTTCGACTTCAAGTACCCCGCATTTTGTGGTAAATTTCTGCTGTGCATAGATGGAAAATATCAATTTGTTCCAGATGCGGCAAAATTTATCACAAAATTAGGTAGAACTGATGTGAGAGATGTAGAAGTTTTGAGTGAGATTTATATCTCTATCAATGACAATTACAAATCTTACAAAGACTTTAAGGTGCTTGATGCTTTGGATAAGGCTTTAGTGGATAGATATCGATCCCCTTATAGTGCTATTTCTGCTTTGGTTTCTTTATGTTATCATATCTTTGACTTTAATAAGTTTAAGTTGCTGTTTAATTGTGAAGGGAAATTTGTGGATAAGAAGCTGAGAAAAGACTTCGAGTGGTGAACTCTAGGTCCTGATGTTTAAATCTACTGTATTTACCTTCGCATGATGGCTACTTTCTCTTGTGTGTGTTGTGGTACCTTAACTACAAGTACTTACTGTGGTAAGAGATGTGAGCGAAAGCATGTATATTCTGAAACAAGAAATAAGAGATTGGAACTTTACAAGAAGTATCTATTGGAACCGCAAAAATGCGCCCTGAATGGAATCGTTGGACACAGTTGTGGAATGCCATGCTCCATTGCGGAAGAGGCTTGTGATCAACTGCCAATCGTGAGTAGGTTCTGTGGCCAAAAGCATGCGGATCTGTATGATTCACTTCTGAAACGTTCTGAACAGGAGTTACTTCTTGTATTTCTCCAGAAGAAGATGCAGGAGCTGAAACTTTCTCATATCGTAAAAATGGCTAAGCTTGAAAGTGAGGTTAACGCAATACGTAAGTCCGTAGCTTCTTCTTTTGAAGATTCTGTTGGATGTGATGATTCTTCTTCCGTTGCTAGCTGATTAATTAA*ctggatgaaaaagcagggtgttcctgatcgagtcaacgacgaggtttttattgcaatgtccaaggccctcaatttcataaaccctgacgagttatccatgcagtgcattctgattgctctaaaccgttctccaggagacgcatggctcgaaaatggcattcttggatggtaatcctcctgaaagtag*GCGGCCGCTCAGCTAGCTAAAAAAAAAAAAAAATGTTTGATCAGATCATTCAAATCTGATGGTGCCCATCAACCATATGATGGGAGTGTTTGCAAGTCCACTATAATCGAACTTGAAAACGATGCCTGAATTGGAAACCATGAATCTTAACGGACTCTGGAGAGAAAATTTAGGAATTGGTATGTAAGCTACAACTTCCGGTAGCTGCGTCACACTTTAAGAGTGTGCATACTGAGCCGAAGCTCAGCTTCGGTCCCCCAAGGGAAGACCAcgcgcgcgtcatgcaagcttggcgtaatcatggtcatagctgtttcctgtgtgaaattgttatccgctcacaattccacacaacatacgagccggaagcataaagtgtaaagcctggggtgcctaatgagtgagctaactcacattaattgcgttgcgctcactgcccgctttccagtcgggaaacctgtcgtgccagctgcattaatgaatcggccaacgcgcggggagaggcggtttgcgtattgggcgctcttccgcttcctcgctcactgactcgctgcgctcggtcgttcggctgcggcgagcggtatcagctcactcaaaggcggtaatacggttatccacagaatcaggggataacgcaggaaagaacatgtgagcaaaaggccagcaaaaggccaggaaccgtaaaaaggccgcgttgctggcgtttttccataggctccgcccccctgacgagcatcacaaaaatcgacgctcaagtcagaggtggcgaaacccgacaggactataaagataccaggcgtttccccctggaagctccctcgtgcgctctcctgttccgaccctgccgcttaccggatacctgtccgcctttctcccttcgggaagcgtggcgctttctcatagctcacgctgtaggtatctcagttcggtgtaggtcgttcgctccaagctgggctgtgtgcacgaaccccccgttcagcccgaccgctgcgccttatccggtaactatcgtcttgagtccaac

pBSMVγQT1 (γQT1 for short)

cgttcagcccgaccgctgcgccttatccggtaactatcgtcttgagtccaacccggtaagacacgacttatcgccactggcagcagccactggtaacaggattagcagagcgaggtatgtaggcggtgctacagagttcttgaagtggtggcctaactacggctacactagaagaacagtatttggtatctgcgctctgctgaagccagttaccttcggaaaaagagttggtagctcttgatccggcaaacaaaccaccgctggtagcggtggtttttttgtttgcaagcagcagattacgcgcagaaaaaaaggatctcaagaagatcctttgatcttttctacggggtctgacgctcagtggaacgaaaactcacgttaagggattttggtcatgagattatcaaaaaggatcttcacctagatccttttaaattaaaaatgaagttttaaatcaatctaaagtatatatgagtaaacttggtctgacagttaccaatgcttaatcagtgaggcacctatctcagcgatctgtctatttcgttcatccatagttgcctgactccccgtcgtgtagataactacgatacgggagggcttaccatctggccccagtgctgcaatgataccgcgagacccacgctcaccggctccagatttatcagcaataaaccagccagccggaagggccgagcgcagaagtggtcctgcaactttatccgcctccatccagtctattaattgttgccgggaagctagagtaagtagttcgccagttaatagtttgcgcaacgttgttgccattgctacaggcatcgtggtgtcacgctcgtcgtttggtatggcttcattcagctccggttcccaacgatcaaggcgagttacatgatcccccatgttgtgcaaaaaagcggttagctccttcggtcctccgatcgttgtcagaagtaagttggccgcagtgttatcactcatggttatggcagcactgcataattctcttactgtcatgccatccgtaagatgcttttctgtgactggtgagtactcaaccaagtcattctgagaatagtgtatgcggcgaccgagttgctcttgcccggcgtcaatacgggataataccgcgccacatagcagaactttaaaagtgctcatcattggaaaacgttcttcggggcgaaaactctcaaggatcttaccgctgttgagatccagttcgatgtaacccactcgtgcacccaactgatcttcagcatcttttactttcaccagcgtttctgggtgagcaaaaacaggaaggcaaaatgccgcaaaaaagggaataagggcgacacggaaatgttgaatactcatactcttcctttttcaatattattgaagcatttatcagggttattgtctcatgagcggatacatatttgaatgtatttagaaaaataaacaaataggggttccgcgcacatttccccgaaaagtgccacctaaattgtaagcgttaatattttgttaaaattcgcgttaaatttttgttaaatcagctcattttttaaccaataggccgaaatcggcaaaatcccttataaatcaaaagaatagaccgagatagggttgagtgttgttccagtttggaacaagagtccactattaaagaacgtggactccaacgtcaaagggcgaaaaaccgtctatcagggcgatggcccactacgtgaaccatcaccctaatcaagttttttggggtcgaggtgccgtaaagcactaaatcggaaccctaaagggagcccccgatttagagcttgacggggaaagccggcgaacgtggcgagaaaggaagggaagaaagcgaaaggagcgggcgctagggcgctggcaagtgtagcggtcacgctgcgcgtaaccaccacacccgccgcgcttaatgcgccgctacagggcgcgtcccattcgccattcaggctgcgcaactgttgggaagggcgatcggtgcgggcctcttcgctattacgccagctggcgaaagggggatgtgctgcaaggcgattaagttgggtaacgccagggttttcccagtcacgacgttgtaaaacgacggccagtgaattaatacgactcactataGTATAGCTTGAGCATTACCGTCGTGTAATTGCAACACTTGGCTTGCCAAATAACGCTAAAGCGTTCACGAAACAAACAACACTTCGGCATGGATGTTGTGAAGAAATTCGCCGTCATGTCAGTGACTGTAGTAGCAGGTCCCGTCCTTACGCTTTCATCACCTGTGGTGGTGACGTTTGGAACAGGCTTAATTGCCGTATCTTTGGTGAAACGGTTGCTACAGGAACAACCCCGTGTAATTGCTCACGATCACGAACATTACCCAGGTGGTTCTGAGAGCAGTTCTAGCTCTTGTGCTACCGCGCCTATTTTACGTAATCTTTCGCGAGATCAGTGCGATTCAGAGAATATTGGATGCAGTTCTAGCGCCTGTTCTCCGTCTGAAATTGTGAAAGTTACAAGGCAGGTAGTGGGAGTTGAACGTGGTCTTTACCGGGACATTTTTCAGGACAACGAAATCCCATCAGTCATGGAAGAGAAACTGCAGAAACTCCTTTACTCTGAGGGTGAGAAGATTCGAAGACGTTGCCAATTTGAAGCATCAACGATGCACTCACGCAAAGTAAAGGTTCCGGAGGTAGGTACTATCCCAGATATCCAAACTTGGTTCGATGCTACGTTTCCTGGTAACTCCGTTAGGTTTTCTGATTTCGACGGTTATACTGTTGCTACGGAGGACATTAACATGGATGTTCAGGATTGTAGACTTAAGTTCGGGAAGACTTTTCGACCTTATGAATTTAAGGAATCACTGAAACCAGTACTGAGGACAGCAATGCCAGAAAAACGACAGGGTAGTTTGATTGAAAGTGTGCTGGCCTTTCGTAAAAGAAATTTGGCTGCGCCCAGATTACAAGGAGCTTTGAATGAATGGCACACAATTGAGAATGTGCTAACGAAGGCGTTAAAGGTATTCTTCTTTGAAGATTTAATTGATCGAACGGATCACTGCACTTACGAGTCAGCGCTCAGATGGTGGGATAAACAATCAGTGACAGCTCGAGCGCAGCTCGTGGCGGATCAGCGGAGGTTATGTGATGTTGACTTCACGACTTATAACTTCATGATAAAAAATGATGTAAAGCCGAAGTTAGATCTAACACCTCAAGTTGAATATGCAGCTTTGCAGACTGTTGTATATCCTGATAAGATAGTCAATGCTTTCTTTGGTCCGATCATAAAGGAGATTAATGAACGGATCATCAGAGCGCTTAGACCTCATGTGGTCTTTAATTCTCGTATGACTGCTGATGAACTGAATGAAACAGCTGCCTTTTTGACACCTCATAAGTACAGAGCCTTAGAGATTGATTTTTCAAAATTTGATAAATCAAAGACTGGGCTTCATATCAAAGCTGTCATTGGACTCTATAAGCTCTTTGGCCTAGATGGCCTGTTAAAAGTGCTCTGGGAAAAATCGCAATATCAGACTTACGTGAAAGATAGAAACTTCGGTCTCGAGGCATATCTATTGTATCAGCAAAAGTCAGGAAATTGTGACACTTACGGTTCGAACACCTGGTCTGCCGCCTTGGCGTTGTTAGATTGTCTTCCTTTGGAAGATGCACATTTCTGTGTATTTGGTGGTGATGATTCATTGATATTGTTTGATCAGGGATACATAATTTCCGACCCATGCCGGCAACTTGCCGGTACTTGGAATCTTGAATGTAAAGTGTTCGACTTCAAGTACCCCGCATTTTGTGGTAAATTTCTGCTGTGCATAGATGGAAAATATCAATTTGTTCCAGATGCGGCAAAATTTATCACAAAATTAGGTAGAACTGATGTGAGAGATGTAGAAGTTTTGAGTGAGATTTATATCTCTATCAATGACAATTACAAATCTTACAAAGACTTTAAGGTGCTTGATGCTTTGGATAAGGCTTTAGTGGATAGATATCGATCCCCTTATAGTGCTATTTCTGCTTTGGTTTCTTTATGTTATCATATCTTTGACTTTAATAAGTTTAAGTTGCTGTTTAATTGTGAAGGGAAATTTGTGGATAAGAAGCTGAGAAAAGACTTCGAGTGGTGAACTCTAGGTCCTGATGTTTAAATCTACTGTATTTACCTTCGCATGATGGCTACTTTCTCTTGTGTGTGTTGTGGTACCTTAACTACAAGTACTTACTGTGGTAAGAGATGTGAGCGAAAGCATGTATATTCTGAAACAAGAAATAAGAGATTGGAACTTTACAAGAAGTATCTATTGGAACCGCAAAAATGCGCCCTGAATGGAATCGTTGGACACAGTTGTGGAATGCCATGCTCCATTGCGGAAGAGGCTTGTGATCAACTGCCAATCGTGAGTAGGTTCTGTGGCCAAAAGCATGCGGATCTGTATGATTCACTTCTGAAACGTTCTGAACAGGAGTTACTTCTTGAATTTCTCCAGAAGAAGATGCAGGAGCTGAAACTTTCTCATATCGTAAAAATGGCTAAGCTTGAAAGTGAGGTTAACGCAATACGTAAGTCCGTAGCTTCTTCTTTTGAAGATTCTGTTGGATGTGATGATTCTTCTTCCGTTGCTAGCTGATTAATTAAcgcttgctgcatcagacttgATGAGGAACTGGACCAAGGgttttagagctagaaatagcaagttaaaataaggctagtccgttatcaacttgaaaaagtggcaccgagtcggtgcGCGGCCGCTCAGCTAGCTAAAAAAAAAAAAAAATGTTTGATCAGATCATTCAAATCTGATGGTGCCCATCAACCATATGATGGGAGTGTTTGCAAGTCCACTATAATCGAACTTGAAAACGATGCCTGAATTGGAAACCATGAATCTTAACGGACTCTGGAGAGAAAATTTAGGAATTGGTATGTAAGCTACAACTTCCGGTAGCTGCGTCACACTTTAAGAGTGTGCATACTGAGCCGAAGCTCAGCTTCGGTCCCCCAAGGGAAGACCAcgcgcgcgtcatgcaagcttggcgtaatcatggtcatagctgtttcctgtgtgaaattgttatccgctcacaattccacacaacatacgagccggaagcataaagtgtaaagcctggggtgcctaatgagtgagctaactcacattaattgcgttgcgctcactgcccgctttccagtcgggaaacctgtcgtgccagctgcattaatgaatcggccaacgcgcggggagaggcggtttgcgtattgggcgctcttccgcttcctcgctcactgactcgctgcgctcggtcgttcggctgcggcgagcggtatcagctcactcaaaggcggtaatacggttatccacagaatcaggggataacgcaggaaagaacatgtgagcaaaaggccagcaaaaggccaggaaccgtaaaaaggccgcgttgctggcgtttttccataggctccgcccccctgacgagcatcacaaaaatcgacgctcaagtcagaggtggcgaaacccgacaggactataaagataccaggcgtttccccctggaagctccctcgtgcgctctcctgttccgaccctgccgcttaccggatacctgtccgcctttctcccttcgggaagcgtggcgctttctcatagctcacgctgtaggtatctcagttcggtgtaggtcgttcgctccaagctgggctgtgtgcacgaaccccccgttcagcccgaccgctgcgccttatccggtaactatcgtcttgagtccaac

pBSMVγGW2T2 (γGW2T2 for short, constructs targeting all target sites except for QT1 are constructed in the same way)

cgttcagcccgaccgctgcgccttatccggtaactatcgtcttgagtccaacccggtaagacacgacttatcgccactggcagcagccactggtaacaggattagcagagcgaggtatgtaggcggtgctacagagttcttgaagtggtggcctaactacggctacactagaagaacagtatttggtatctgcgctctgctgaagccagttaccttcggaaaaagagttggtagctcttgatccggcaaacaaaccaccgctggtagcggtggtttttttgtttgcaagcagcagattacgcgcagaaaaaaaggatctcaagaagatcctttgatcttttctacggggtctgacgctcagtggaacgaaaactcacgttaagggattttggtcatgagattatcaaaaaggatcttcacctagatccttttaaattaaaaatgaagttttaaatcaatctaaagtatatatgagtaaacttggtctgacagttaccaatgcttaatcagtgaggcacctatctcagcgatctgtctatttcgttcatccatagttgcctgactccccgtcgtgtagataactacgatacgggagggcttaccatctggccccagtgctgcaatgataccgcgagacccacgctcaccggctccagatttatcagcaataaaccagccagccggaagggccgagcgcagaagtggtcctgcaactttatccgcctccatccagtctattaattgttgccgggaagctagagtaagtagttcgccagttaatagtttgcgcaacgttgttgccattgctacaggcatcgtggtgtcacgctcgtcgtttggtatggcttcattcagctccggttcccaacgatcaaggcgagttacatgatcccccatgttgtgcaaaaaagcggttagctccttcggtcctccgatcgttgtcagaagtaagttggccgcagtgttatcactcatggttatggcagcactgcataattctcttactgtcatgccatccgtaagatgcttttctgtgactggtgagtactcaaccaagtcattctgagaatagtgtatgcggcgaccgagttgctcttgcccggcgtcaatacgggataataccgcgccacatagcagaactttaaaagtgctcatcattggaaaacgttcttcggggcgaaaactctcaaggatcttaccgctgttgagatccagttcgatgtaacccactcgtgcacccaactgatcttcagcatcttttactttcaccagcgtttctgggtgagcaaaaacaggaaggcaaaatgccgcaaaaaagggaataagggcgacacggaaatgttgaatactcatactcttcctttttcaatattattgaagcatttatcagggttattgtctcatgagcggatacatatttgaatgtatttagaaaaataaacaaataggggttccgcgcacatttccccgaaaagtgccacctaaattgtaagcgttaatattttgttaaaattcgcgttaaatttttgttaaatcagctcattttttaaccaataggccgaaatcggcaaaatcccttataaatcaaaagaatagaccgagatagggttgagtgttgttccagtttggaacaagagtccactattaaagaacgtggactccaacgtcaaagggcgaaaaaccgtctatcagggcgatggcccactacgtgaaccatcaccctaatcaagttttttggggtcgaggtgccgtaaagcactaaatcggaaccctaaagggagcccccgatttagagcttgacggggaaagccggcgaacgtggcgagaaaggaagggaagaaagcgaaaggagcgggcgctagggcgctggcaagtgtagcggtcacgctgcgcgtaaccaccacacccgccgcgcttaatgcgccgctacagggcgcgtcccattcgccattcaggctgcgcaactgttgggaagggcgatcggtgcgggcctcttcgctattacgccagctggcgaaagggggatgtgctgcaaggcgattaagttgggtaacgccagggttttcccagtcacgacgttgtaaaacgacggccagtgaattaatacgactcactataGTATAGCTTGAGCATTACCGTCGTGTAATTGCAACACTTGGCTTGCCAAATAACGCTAAAGCGTTCACGAAACAAACAACACTTCGGCATGGATGTTGTGAAGAAATTCGCCGTCATGTCAGTGACTGTAGTAGCAGGTCCCGTCCTTACGCTTTCATCACCTGTGGTGGTGACGTTTGGAACAGGCTTAATTGCCGTATCTTTGGTGAAACGGTTGCTACAGGAACAACCCCGTGTAATTGCTCACGATCACGAACATTACCCAGGTGGTTCTGAGAGCAGTTCTAGCTCTTGTGCTACCGCGCCTATTTTACGTAATCTTTCGCGAGATCAGTGCGATTCAGAGAATATTGGATGCAGTTCTAGCGCCTGTTCTCCGTCTGAAATTGTGAAAGTTACAAGGCAGGTAGTGGGAGTTGAACGTGGTCTTTACCGGGACATTTTTCAGGACAACGAAATCCCATCAGTCATGGAAGAGAAACTGCAGAAACTCCTTTACTCTGAGGGTGAGAAGATTCGAAGACGTTGCCAATTTGAAGCATCAACGATGCACTCACGCAAAGTAAAGGTTCCGGAGGTAGGTACTATCCCAGATATCCAAACTTGGTTCGATGCTACGTTTCCTGGTAACTCCGTTAGGTTTTCTGATTTCGACGGTTATACTGTTGCTACGGAGGACATTAACATGGATGTTCAGGATTGTAGACTTAAGTTCGGGAAGACTTTTCGACCTTATGAATTTAAGGAATCACTGAAACCAGTACTGAGGACAGCAATGCCAGAAAAACGACAGGGTAGTTTGATTGAAAGTGTGCTGGCCTTTCGTAAAAGAAATTTGGCTGCGCCCAGATTACAAGGAGCTTTGAATGAATGGCACACAATTGAGAATGTGCTAACGAAGGCGTTAAAGGTATTCTTCTTTGAAGATTTAATTGATCGAACGGATCACTGCACTTACGAGTCAGCGCTCAGATGGTGGGATAAACAATCAGTGACAGCTCGAGCGCAGCTCGTGGCGGATCAGCGGAGGTTATGTGATGTTGACTTCACGACTTATAACTTCATGATAAAAAATGATGTAAAGCCGAAGTTAGATCTAACACCTCAAGTTGAATATGCAGCTTTGCAGACTGTTGTATATCCTGATAAGATAGTCAATGCTTTCTTTGGTCCGATCATAAAGGAGATTAATGAACGGATCATCAGAGCGCTTAGACCTCATGTGGTCTTTAATTCTCGTATGACTGCTGATGAACTGAATGAAACAGCTGCCTTTTTGACACCTCATAAGTACAGAGCCTTAGAGATTGATTTTTCAAAATTTGATAAATCAAAGACTGGGCTTCATATCAAAGCTGTCATTGGACTCTATAAGCTCTTTGGCCTAGATGGCCTGTTAAAAGTGCTCTGGGAAAAATCGCAATATCAGACTTACGTGAAAGATAGAAACTTCGGTCTCGAGGCATATCTATTGTATCAGCAAAAGTCAGGAAATTGTGACACTTACGGTTCGAACACCTGGTCTGCCGCCTTGGCGTTGTTAGATTGTCTTCCTTTGGAAGATGCACATTTCTGTGTATTTGGTGGTGATGATTCATTGATATTGTTTGATCAGGGATACATAATTTCCGACCCATGCCGGCAACTTGCCGGTACTTGGAATCTTGAATGTAAAGTGTTCGACTTCAAGTACCCCGCATTTTGTGGTAAATTTCTGCTGTGCATAGATGGAAAATATCAATTTGTTCCAGATGCGGCAAAATTTATCACAAAATTAGGTAGAACTGATGTGAGAGATGTAGAAGTTTTGAGTGAGATTTATATCTCTATCAATGACAATTACAAATCTTACAAAGACTTTAAGGTGCTTGATGCTTTGGATAAGGCTTTAGTGGATAGATATCGATCCCCTTATAGTGCTATTTCTGCTTTGGTTTCTTTATGTTATCATATCTTTGACTTTAATAAGTTTAAGTTGCTGTTTAATTGTGAAGGGAAATTTGTGGATAAGAAGCTGAGAAAAGACTTCGAGTGGTGAACTCTAGGTCCTGATGTTTAAATCTACTGTATTTACCTTCGCATGATGGCTACTTTCTCTTGTGTGTGTTGTGGTACCTTAACTACAAGTACTTACTGTGGTAAGAGATGTGAGCGAAAGCATGTATATTCTGAAACAAGAAATAAGAGATTGGAACTTTACAAGAAGTATCTATTGGAACCGCAAAAATGCGCCCTGAATGGAATCGTTGGACACAGTTGTGGAATGCCATGCTCCATTGCGGAAGAGGCTTGTGATCAACTGCCAATCGTGAGTAGGTTCTGTGGCCAAAAGCATGCGGATCTGTATGATTCACTTCTGAAACGTTCTGAACAGGAGTTACTTCTTGAATTTCTCCAGAAGAAGATGCAGGAGCTGAAACTTTCTCATATCGTAAAAATGGCTAAGCTTGAAAGTGAGGTTAACGCAATACGTAAGTCCGTAGCTTCTTCTTTTGAAGATTCTGTTGGATGTGATGATTCTTCTTCCGTTGCTAGCTGATTAATTAAGCTCGCGCCCTGCTACCCGGgttttagagctagaaatagcaagttaaaataaggctagtccgttatcaacttgaaaaagtggcaccgagtcggtgcGCGGCCGCTCAGCTAGCTAAAAAAAAAAAAAAATGTTTGATCAGATCATTCAAATCTGATGGTGCCCATCAACCATATGATGGGAGTGTTTGCAAGTCCACTATAATCGAACTTGAAAACGATGCCTGAATTGGAAACCATGAATCTTAACGGACTCTGGAGAGAAAATTTAGGAATTGGTATGTAAGCTACAACTTCCGGTAGCTGCGTCACACTTTAAGAGTGTGCATACTGAGCCGAAGCTCAGCTTCGGTCCCCCAAGGGAAGACCAcgcgcgcgtcatgcaagcttggcgtaatcatggtcatagctgtttcctgtgtgaaattgttatccgctcacaattccacacaacatacgagccggaagcataaagtgtaaagcctggggtgcctaatgagtgagctaactcacattaattgcgttgcgctcactgcccgctttccagtcgggaaacctgtcgtgccagctgcattaatgaatcggccaacgcgcggggagaggcggtttgcgtattgggcgctcttccgcttcctcgctcactgactcgctgcgctcggtcgttcggctgcggcgagcggtatcagctcactcaaaggcggtaatacggttatccacagaatcaggggataacgcaggaaagaacatgtgagcaaaaggccagcaaaaggccaggaaccgtaaaaaggccgcgttgctggcgtttttccataggctccgcccccctgacgagcatcacaaaaatcgacgctcaagtcagaggtggcgaaacccgacaggactataaagataccaggcgtttccccctggaagctccctcgtgcgctctcctgttccgaccctgccgcttaccggatacctgtccgcctttctcccttcgggaagcgtggcgctttctcatagctcacgctgtaggtatctcagttcggtgtaggtcgttcgctccaagctgggctgtgtgcacgaaccccccgttcagcccgaccgctgcgccttatccggtaactatcgtcttgagtccaac

pBSMVγGW2T2FT (γGW2T2FT for short)

cgttcagcccgaccgctgcgccttatccggtaactatcgtcttgagtccaacccggtaagacacgacttatcgccactggcagcagccactggtaacaggattagcagagcgaggtatgtaggcggtgctacagagttcttgaagtggtggcctaactacggctacactagaagaacagtatttggtatctgcgctctgctgaagccagttaccttcggaaaaagagttggtagctcttgatccggcaaacaaaccaccgctggtagcggtggtttttttgtttgcaagcagcagattacgcgcagaaaaaaaggatctcaagaagatcctttgatcttttctacggggtctgacgctcagtggaacgaaaactcacgttaagggattttggtcatgagattatcaaaaaggatcttcacctagatccttttaaattaaaaatgaagttttaaatcaatctaaagtatatatgagtaaacttggtctgacagttaccaatgcttaatcagtgaggcacctatctcagcgatctgtctatttcgttcatccatagttgcctgactccccgtcgtgtagataactacgatacgggagggcttaccatctggccccagtgctgcaatgataccgcgagacccacgctcaccggctccagatttatcagcaataaaccagccagccggaagggccgagcgcagaagtggtcctgcaactttatccgcctccatccagtctattaattgttgccgggaagctagagtaagtagttcgccagttaatagtttgcgcaacgttgttgccattgctacaggcatcgtggtgtcacgctcgtcgtttggtatggcttcattcagctccggttcccaacgatcaaggcgagttacatgatcccccatgttgtgcaaaaaagcggttagctccttcggtcctccgatcgttgtcagaagtaagttggccgcagtgttatcactcatggttatggcagcactgcataattctcttactgtcatgccatccgtaagatgcttttctgtgactggtgagtactcaaccaagtcattctgagaatagtgtatgcggcgaccgagttgctcttgcccggcgtcaatacgggataataccgcgccacatagcagaactttaaaagtgctcatcattggaaaacgttcttcggggcgaaaactctcaaggatcttaccgctgttgagatccagttcgatgtaacccactcgtgcacccaactgatcttcagcatcttttactttcaccagcgtttctgggtgagcaaaaacaggaaggcaaaatgccgcaaaaaagggaataagggcgacacggaaatgttgaatactcatactcttcctttttcaatattattgaagcatttatcagggttattgtctcatgagcggatacatatttgaatgtatttagaaaaataaacaaataggggttccgcgcacatttccccgaaaagtgccacctaaattgtaagcgttaatattttgttaaaattcgcgttaaatttttgttaaatcagctcattttttaaccaataggccgaaatcggcaaaatcccttataaatcaaaagaatagaccgagatagggttgagtgttgttccagtttggaacaagagtccactattaaagaacgtggactccaacgtcaaagggcgaaaaaccgtctatcagggcgatggcccactacgtgaaccatcaccctaatcaagttttttggggtcgaggtgccgtaaagcactaaatcggaaccctaaagggagcccccgatttagagcttgacggggaaagccggcgaacgtggcgagaaaggaagggaagaaagcgaaaggagcgggcgctagggcgctggcaagtgtagcggtcacgctgcgcgtaaccaccacacccgccgcgcttaatgcgccgctacagggcgcgtcccattcgccattcaggctgcgcaactgttgggaagggcgatcggtgcgggcctcttcgctattacgccagctggcgaaagggggatgtgctgcaaggcgattaagttgggtaacgccagggttttcccagtcacgacgttgtaaaacgacggccagtgaattaatacgactcactataGTATAGCTTGAGCATTACCGTCGTGTAATTGCAACACTTGGCTTGCCAAATAACGCTAAAGCGTTCACGAAACAAACAACACTTCGGCATGGATGTTGTGAAGAAATTCGCCGTCATGTCAGTGACTGTAGTAGCAGGTCCCGTCCTTACGCTTTCATCACCTGTGGTGGTGACGTTTGGAACAGGCTTAATTGCCGTATCTTTGGTGAAACGGTTGCTACAGGAACAACCCCGTGTAATTGCTCACGATCACGAACATTACCCAGGTGGTTCTGAGAGCAGTTCTAGCTCTTGTGCTACCGCGCCTATTTTACGTAATCTTTCGCGAGATCAGTGCGATTCAGAGAATATTGGATGCAGTTCTAGCGCCTGTTCTCCGTCTGAAATTGTGAAAGTTACAAGGCAGGTAGTGGGAGTTGAACGTGGTCTTTACCGGGACATTTTTCAGGACAACGAAATCCCATCAGTCATGGAAGAGAAACTGCAGAAACTCCTTTACTCTGAGGGTGAGAAGATTCGAAGACGTTGCCAATTTGAAGCATCAACGATGCACTCACGCAAAGTAAAGGTTCCGGAGGTAGGTACTATCCCAGATATCCAAACTTGGTTCGATGCTACGTTTCCTGGTAACTCCGTTAGGTTTTCTGATTTCGACGGTTATACTGTTGCTACGGAGGACATTAACATGGATGTTCAGGATTGTAGACTTAAGTTCGGGAAGACTTTTCGACCTTATGAATTTAAGGAATCACTGAAACCAGTACTGAGGACAGCAATGCCAGAAAAACGACAGGGTAGTTTGATTGAAAGTGTGCTGGCCTTTCGTAAAAGAAATTTGGCTGCGCCCAGATTACAAGGAGCTTTGAATGAATGGCACACAATTGAGAATGTGCTAACGAAGGCGTTAAAGGTATTCTTCTTTGAAGATTTAATTGATCGAACGGATCACTGCACTTACGAGTCAGCGCTCAGATGGTGGGATAAACAATCAGTGACAGCTCGAGCGCAGCTCGTGGCGGATCAGCGGAGGTTATGTGATGTTGACTTCACGACTTATAACTTCATGATAAAAAATGATGTAAAGCCGAAGTTAGATCTAACACCTCAAGTTGAATATGCAGCTTTGCAGACTGTTGTATATCCTGATAAGATAGTCAATGCTTTCTTTGGTCCGATCATAAAGGAGATTAATGAACGGATCATCAGAGCGCTTAGACCTCATGTGGTCTTTAATTCTCGTATGACTGCTGATGAACTGAATGAAACAGCTGCCTTTTTGACACCTCATAAGTACAGAGCCTTAGAGATTGATTTTTCAAAATTTGATAAATCAAAGACTGGGCTTCATATCAAAGCTGTCATTGGACTCTATAAGCTCTTTGGCCTAGATGGCCTGTTAAAAGTGCTCTGGGAAAAATCGCAATATCAGACTTACGTGAAAGATAGAAACTTCGGTCTCGAGGCATATCTATTGTATCAGCAAAAGTCAGGAAATTGTGACACTTACGGTTCGAACACCTGGTCTGCCGCCTTGGCGTTGTTAGATTGTCTTCCTTTGGAAGATGCACATTTCTGTGTATTTGGTGGTGATGATTCATTGATATTGTTTGATCAGGGATACATAATTTCCGACCCATGCCGGCAACTTGCCGGTACTTGGAATCTTGAATGTAAAGTGTTCGACTTCAAGTACCCCGCATTTTGTGGTAAATTTCTGCTGTGCATAGATGGAAAATATCAATTTGTTCCAGATGCGGCAAAATTTATCACAAAATTAGGTAGAACTGATGTGAGAGATGTAGAAGTTTTGAGTGAGATTTATATCTCTATCAATGACAATTACAAATCTTACAAAGACTTTAAGGTGCTTGATGCTTTGGATAAGGCTTTAGTGGATAGATATCGATCCCCTTATAGTGCTATTTCTGCTTTGGTTTCTTTATGTTATCATATCTTTGACTTTAATAAGTTTAAGTTGCTGTTTAATTGTGAAGGGAAATTTGTGGATAAGAAGCTGAGAAAAGACTTCGAGTGGTGAACTCTAGGTCCTGATGTTTAAATCTACTGTATTTACCTTCGCATGATGGCTACTTTCTCTTGTGTGTGTTGTGGTACCTTAACTACAAGTACTTACTGTGGTAAGAGATGTGAGCGAAAGCATGTATATTCTGAAACAAGAAATAAGAGATTGGAACTTTACAAGAAGTATCTATTGGAACCGCAAAAATGCGCCCTGAATGGAATCGTTGGACACAGTTGTGGAATGCCATGCTCCATTGCGGAAGAGGCTTGTGATCAACTGCCAATCGTGAGTAGGTTCTGTGGCCAAAAGCATGCGGATCTGTATGATTCACTTCTGAAACGTTCTGAACAGGAGTTACTTCTTGAATTTCTCCAGAAGAAGATGCAGGAGCTGAAACTTTCTCATATCGTAAAAATGGCTAAGCTTGAAAGTGAGGTTAACGCAATACGTAAGTCCGTAGCTTCTTCTTTTGAAGATTCTGTTGGATGTGATGATTCTTCTTCCGTTGCTAGCTGATTAATTAAGCTCGCGCCCTGCTACCCGGgttttagagctagaaatagcaagttaaaataaggctagtccgttatcaacttgaaaaagtggcaccgagtcggtgcatgtctataaatataagagaccctcttatagtaagcagagttgttggagacgttcttgatccgtttaatagatcaatcactctaaaggttacttatggccaaagagaggtgactaatggcttggatctaaggccttctcaggttcaaaacaagccaagagttgagattggtggagaagacctcaggaacttctatactttggttatggtggatccagatgttccaagtcctagcaaccctcacctccgagaatatctccattggttggtgactgatatccctgctacaactggaacaacctttggcaatgagattgtgtgttacgaaaatccaagtcccactgcaggaattcatcgtgtcgtgtttatattgtttcgacagcttggcaggcaaacagtgtatgcaccagggtggcgccagaacttcaacactcgcgagtttgctgagatctacaatctcggccttcccgtggccgcagttttctacaattgtcagagggagagtggctgcggaggaagaagactttagGCGGCCGCTCAGCTAGCTAAAAAAAAAAAAAAATGTTTGATCAGATCATTCAAATCTGATGGTGCCCATCAACCATATGATGGGAGTGTTTGCAAGTCCACTATAATCGAACTTGAAAACGATGCCTGAATTGGAAACCATGAATCTTAACGGACTCTGGAGAGAAAATTTAGGAATTGGTATGTAAGCTACAACTTCCGGTAGCTGCGTCACACTTTAAGAGTGTGCATACTGAGCCGAAGCTCAGCTTCGGTCCCCCAAGGGAAGACCAcgcgcgcgtcatgcaagcttggcgtaatcatggtcatagctgtttcctgtgtgaaattgttatccgctcacaattccacacaacatacgagccggaagcataaagtgtaaagcctggggtgcctaatgagtgagctaactcacattaattgcgttgcgctcactgcccgctttccagtcgggaaacctgtcgtgccagctgcattaatgaatcggccaacgcgcggggagaggcggtttgcgtattgggcgctcttccgcttcctcgctcactgactcgctgcgctcggtcgttcggctgcggcgagcggtatcagctcactcaaaggcggtaatacggttatccacagaatcaggggataacgcaggaaagaacatgtgagcaaaaggccagcaaaaggccaggaaccgtaaaaaggccgcgttgctggcgtttttccataggctccgcccccctgacgagcatcacaaaaatcgacgctcaagtcagaggtggcgaaacccgacaggactataaagataccaggcgtttccccctggaagctccctcgtgcgctctcctgttccgaccctgccgcttaccggatacctgtccgcctttctcccttcgggaagcgtggcgctttctcatagctcacgctgtaggtatctcagttcggtgtaggtcgttcgctccaagctgggctgtgtgcacgaaccccccgttcagcccgaccgctgcgccttatccggtaactatcgtcttgagtccaac

pBSMVγGW2T2Vrn (γGW2T2Vrn for short)

cgttcagcccgaccgctgcgccttatccggtaactatcgtcttgagtccaacccggtaagacacgacttatcgccactggcagcagccactggtaacaggattagcagagcgaggtatgtaggcggtgctacagagttcttgaagtggtggcctaactacggctacactagaagaacagtatttggtatctgcgctctgctgaagccagttaccttcggaaaaagagttggtagctcttgatccggcaaacaaaccaccgctggtagcggtggtttttttgtttgcaagcagcagattacgcgcagaaaaaaaggatctcaagaagatcctttgatcttttctacggggtctgacgctcagtggaacgaaaactcacgttaagggattttggtcatgagattatcaaaaaggatcttcacctagatccttttaaattaaaaatgaagttttaaatcaatctaaagtatatatgagtaaacttggtctgacagttaccaatgcttaatcagtgaggcacctatctcagcgatctgtctatttcgttcatccatagttgcctgactccccgtcgtgtagataactacgatacgggagggcttaccatctggccccagtgctgcaatgataccgcgagacccacgctcaccggctccagatttatcagcaataaaccagccagccggaagggccgagcgcagaagtggtcctgcaactttatccgcctccatccagtctattaattgttgccgggaagctagagtaagtagttcgccagttaatagtttgcgcaacgttgttgccattgctacaggcatcgtggtgtcacgctcgtcgtttggtatggcttcattcagctccggttcccaacgatcaaggcgagttacatgatcccccatgttgtgcaaaaaagcggttagctccttcggtcctccgatcgttgtcagaagtaagttggccgcagtgttatcactcatggttatggcagcactgcataattctcttactgtcatgccatccgtaagatgcttttctgtgactggtgagtactcaaccaagtcattctgagaatagtgtatgcggcgaccgagttgctcttgcccggcgtcaatacgggataataccgcgccacatagcagaactttaaaagtgctcatcattggaaaacgttcttcggggcgaaaactctcaaggatcttaccgctgttgagatccagttcgatgtaacccactcgtgcacccaactgatcttcagcatcttttactttcaccagcgtttctgggtgagcaaaaacaggaaggcaaaatgccgcaaaaaagggaataagggcgacacggaaatgttgaatactcatactcttcctttttcaatattattgaagcatttatcagggttattgtctcatgagcggatacatatttgaatgtatttagaaaaataaacaaataggggttccgcgcacatttccccgaaaagtgccacctaaattgtaagcgttaatattttgttaaaattcgcgttaaatttttgttaaatcagctcattttttaaccaataggccgaaatcggcaaaatcccttataaatcaaaagaatagaccgagatagggttgagtgttgttccagtttggaacaagagtccactattaaagaacgtggactccaacgtcaaagggcgaaaaaccgtctatcagggcgatggcccactacgtgaaccatcaccctaatcaagttttttggggtcgaggtgccgtaaagcactaaatcggaaccctaaagggagcccccgatttagagcttgacggggaaagccggcgaacgtggcgagaaaggaagggaagaaagcgaaaggagcgggcgctagggcgctggcaagtgtagcggtcacgctgcgcgtaaccaccacacccgccgcgcttaatgcgccgctacagggcgcgtcccattcgccattcaggctgcgcaactgttgggaagggcgatcggtgcgggcctcttcgctattacgccagctggcgaaagggggatgtgctgcaaggcgattaagttgggtaacgccagggttttcccagtcacgacgttgtaaaacgacggccagtgaattaatacgactcactataGTATAGCTTGAGCATTACCGTCGTGTAATTGCAACACTTGGCTTGCCAAATAACGCTAAAGCGTTCACGAAACAAACAACACTTCGGCATGGATGTTGTGAAGAAATTCGCCGTCATGTCAGTGACTGTAGTAGCAGGTCCCGTCCTTACGCTTTCATCACCTGTGGTGGTGACGTTTGGAACAGGCTTAATTGCCGTATCTTTGGTGAAACGGTTGCTACAGGAACAACCCCGTGTAATTGCTCACGATCACGAACATTACCCAGGTGGTTCTGAGAGCAGTTCTAGCTCTTGTGCTACCGCGCCTATTTTACGTAATCTTTCGCGAGATCAGTGCGATTCAGAGAATATTGGATGCAGTTCTAGCGCCTGTTCTCCGTCTGAAATTGTGAAAGTTACAAGGCAGGTAGTGGGAGTTGAACGTGGTCTTTACCGGGACATTTTTCAGGACAACGAAATCCCATCAGTCATGGAAGAGAAACTGCAGAAACTCCTTTACTCTGAGGGTGAGAAGATTCGAAGACGTTGCCAATTTGAAGCATCAACGATGCACTCACGCAAAGTAAAGGTTCCGGAGGTAGGTACTATCCCAGATATCCAAACTTGGTTCGATGCTACGTTTCCTGGTAACTCCGTTAGGTTTTCTGATTTCGACGGTTATACTGTTGCTACGGAGGACATTAACATGGATGTTCAGGATTGTAGACTTAAGTTCGGGAAGACTTTTCGACCTTATGAATTTAAGGAATCACTGAAACCAGTACTGAGGACAGCAATGCCAGAAAAACGACAGGGTAGTTTGATTGAAAGTGTGCTGGCCTTTCGTAAAAGAAATTTGGCTGCGCCCAGATTACAAGGAGCTTTGAATGAATGGCACACAATTGAGAATGTGCTAACGAAGGCGTTAAAGGTATTCTTCTTTGAAGATTTAATTGATCGAACGGATCACTGCACTTACGAGTCAGCGCTCAGATGGTGGGATAAACAATCAGTGACAGCTCGAGCGCAGCTCGTGGCGGATCAGCGGAGGTTATGTGATGTTGACTTCACGACTTATAACTTCATGATAAAAAATGATGTAAAGCCGAAGTTAGATCTAACACCTCAAGTTGAATATGCAGCTTTGCAGACTGTTGTATATCCTGATAAGATAGTCAATGCTTTCTTTGGTCCGATCATAAAGGAGATTAATGAACGGATCATCAGAGCGCTTAGACCTCATGTGGTCTTTAATTCTCGTATGACTGCTGATGAACTGAATGAAACAGCTGCCTTTTTGACACCTCATAAGTACAGAGCCTTAGAGATTGATTTTTCAAAATTTGATAAATCAAAGACTGGGCTTCATATCAAAGCTGTCATTGGACTCTATAAGCTCTTTGGCCTAGATGGCCTGTTAAAAGTGCTCTGGGAAAAATCGCAATATCAGACTTACGTGAAAGATAGAAACTTCGGTCTCGAGGCATATCTATTGTATCAGCAAAAGTCAGGAAATTGTGACACTTACGGTTCGAACACCTGGTCTGCCGCCTTGGCGTTGTTAGATTGTCTTCCTTTGGAAGATGCACATTTCTGTGTATTTGGTGGTGATGATTCATTGATATTGTTTGATCAGGGATACATAATTTCCGACCCATGCCGGCAACTTGCCGGTACTTGGAATCTTGAATGTAAAGTGTTCGACTTCAAGTACCCCGCATTTTGTGGTAAATTTCTGCTGTGCATAGATGGAAAATATCAATTTGTTCCAGATGCGGCAAAATTTATCACAAAATTAGGTAGAACTGATGTGAGAGATGTAGAAGTTTTGAGTGAGATTTATATCTCTATCAATGACAATTACAAATCTTACAAAGACTTTAAGGTGCTTGATGCTTTGGATAAGGCTTTAGTGGATAGATATCGATCCCCTTATAGTGCTATTTCTGCTTTGGTTTCTTTATGTTATCATATCTTTGACTTTAATAAGTTTAAGTTGCTGTTTAATTGTGAAGGGAAATTTGTGGATAAGAAGCTGAGAAAAGACTTCGAGTGGTGAACTCTAGGTCCTGATGTTTAAATCTACTGTATTTACCTTCGCATGATGGCTACTTTCTCTTGTGTGTGTTGTGGTACCTTAACTACAAGTACTTACTGTGGTAAGAGATGTGAGCGAAAGCATGTATATTCTGAAACAAGAAATAAGAGATTGGAACTTTACAAGAAGTATCTATTGGAACCGCAAAAATGCGCCCTGAATGGAATCGTTGGACACAGTTGTGGAATGCCATGCTCCATTGCGGAAGAGGCTTGTGATCAACTGCCAATCGTGAGTAGGTTCTGTGGCCAAAAGCATGCGGATCTGTATGATTCACTTCTGAAACGTTCTGAACAGGAGTTACTTCTTGAATTTCTCCAGAAGAAGATGCAGGAGCTGAAACTTTCTCATATCGTAAAAATGGCTAAGCTTGAAAGTGAGGTTAACGCAATACGTAAGTCCGTAGCTTCTTCTTTTGAAGATTCTGTTGGATGTGATGATTCTTCTTCCGTTGCTAGCTGATTAATTAAGCTCGCGCCCTGCTACCCGGgttttagagctagaaatagcaagttaaaataaggctagtccgttatcaacttgaaaaagtggcaccgagtcggtgcatggccggtagggatagggacccgctggtggttggcagggttgtgggggacgtgctggaccccttcgtccggaccaccaacctcagggtgaccttcgggaacaggaccgtgtccaacggctgcgagctcaagccgtccatggtcgcccagcagcccagggttgaggtgggcggcaatgagatgaggaccttctacacactcgtgatggtagacccagatgctccaagtccaagcgatcccaaccttagggagtatctccactggcttgtgacagatatccccggtacaactggtgcgtcgttcgggcaggaggtgatgtgctacgagagccctcgtccgaccatggggatccaccgcttcgtgctcgtactcttccagcagctcgggcggcagacggtgtacgcccccgggtggcgccagaacttcaacaccagggacttcgccgagctctacaacctcggcccgcctgtcgccgccgtctacttcaactgccagcgtgaggccggctccggcggcaggaggatgtacaattgaGCGGCCGCTCAGCTAGCTAAAAAAAAAAAAAAATGTTTGATCAGATCATTCAAATCTGATGGTGCCCATCAACCATATGATGGGAGTGTTTGCAAGTCCACTATAATCGAACTTGAAAACGATGCCTGAATTGGAAACCATGAATCTTAACGGACTCTGGAGAGAAAATTTAGGAATTGGTATGTAAGCTACAACTTCCGGTAGCTGCGTCACACTTTAAGAGTGTGCATACTGAGCCGAAGCTCAGCTTCGGTCCCCCAAGGGAAGACCAcgcgcgcgtcatgcaagcttggcgtaatcatggtcatagctgtttcctgtgtgaaattgttatccgctcacaattccacacaacatacgagccggaagcataaagtgtaaagcctggggtgcctaatgagtgagctaactcacattaattgcgttgcgctcactgcccgctttccagtcgggaaacctgtcgtgccagctgcattaatgaatcggccaacgcgcggggagaggcggtttgcgtattgggcgctcttccgcttcctcgctcactgactcgctgcgctcggtcgttcggctgcggcgagcggtatcagctcactcaaaggcggtaatacggttatccacagaatcaggggataacgcaggaaagaacatgtgagcaaaaggccagcaaaaggccaggaaccgtaaaaaggccgcgttgctggcgtttttccataggctccgcccccctgacgagcatcacaaaaatcgacgctcaagtcagaggtggcgaaacccgacaggactataaagataccaggcgtttccccctggaagctccctcgtgcgctctcctgttccgaccctgccgcttaccggatacctgtccgcctttctcccttcgggaagcgtggcgctttctcatagctcacgctgtaggtatctcagttcggtgtaggtcgttcgctccaagctgggctgtgtgcacgaaccccccgttcagcccgaccgctgcgccttatccggtaactatcgtcttgagtccaac

pBSMVγGW2T2tRNA^met^ (γGW2T2Met for short)

cgttcagcccgaccgctgcgccttatccggtaactatcgtcttgagtccaacccggtaagacacgacttatcgccactggcagcagccactggtaacaggattagcagagcgaggtatgtaggcggtgctacagagttcttgaagtggtggcctaactacggctacactagaagaacagtatttggtatctgcgctctgctgaagccagttaccttcggaaaaagagttggtagctcttgatccggcaaacaaaccaccgctggtagcggtggtttttttgtttgcaagcagcagattacgcgcagaaaaaaaggatctcaagaagatcctttgatcttttctacggggtctgacgctcagtggaacgaaaactcacgttaagggattttggtcatgagattatcaaaaaggatcttcacctagatccttttaaattaaaaatgaagttttaaatcaatctaaagtatatatgagtaaacttggtctgacagttaccaatgcttaatcagtgaggcacctatctcagcgatctgtctatttcgttcatccatagttgcctgactccccgtcgtgtagataactacgatacgggagggcttaccatctggccccagtgctgcaatgataccgcgagacccacgctcaccggctccagatttatcagcaataaaccagccagccggaagggccgagcgcagaagtggtcctgcaactttatccgcctccatccagtctattaattgttgccgggaagctagagtaagtagttcgccagttaatagtttgcgcaacgttgttgccattgctacaggcatcgtggtgtcacgctcgtcgtttggtatggcttcattcagctccggttcccaacgatcaaggcgagttacatgatcccccatgttgtgcaaaaaagcggttagctccttcggtcctccgatcgttgtcagaagtaagttggccgcagtgttatcactcatggttatggcagcactgcataattctcttactgtcatgccatccgtaagatgcttttctgtgactggtgagtactcaaccaagtcattctgagaatagtgtatgcggcgaccgagttgctcttgcccggcgtcaatacgggataataccgcgccacatagcagaactttaaaagtgctcatcattggaaaacgttcttcggggcgaaaactctcaaggatcttaccgctgttgagatccagttcgatgtaacccactcgtgcacccaactgatcttcagcatcttttactttcaccagcgtttctgggtgagcaaaaacaggaaggcaaaatgccgcaaaaaagggaataagggcgacacggaaatgttgaatactcatactcttcctttttcaatattattgaagcatttatcagggttattgtctcatgagcggatacatatttgaatgtatttagaaaaataaacaaataggggttccgcgcacatttccccgaaaagtgccacctaaattgtaagcgttaatattttgttaaaattcgcgttaaatttttgttaaatcagctcattttttaaccaataggccgaaatcggcaaaatcccttataaatcaaaagaatagaccgagatagggttgagtgttgttccagtttggaacaagagtccactattaaagaacgtggactccaacgtcaaagggcgaaaaaccgtctatcagggcgatggcccactacgtgaaccatcaccctaatcaagttttttggggtcgaggtgccgtaaagcactaaatcggaaccctaaagggagcccccgatttagagcttgacggggaaagccggcgaacgtggcgagaaaggaagggaagaaagcgaaaggagcgggcgctagggcgctggcaagtgtagcggtcacgctgcgcgtaaccaccacacccgccgcgcttaatgcgccgctacagggcgcgtcccattcgccattcaggctgcgcaactgttgggaagggcgatcggtgcgggcctcttcgctattacgccagctggcgaaagggggatgtgctgcaaggcgattaagttgggtaacgccagggttttcccagtcacgacgttgtaaaacgacggccagtgaattaatacgactcactataGTATAGCTTGAGCATTACCGTCGTGTAATTGCAACACTTGGCTTGCCAAATAACGCTAAAGCGTTCACGAAACAAACAACACTTCGGCATGGATGTTGTGAAGAAATTCGCCGTCATGTCAGTGACTGTAGTAGCAGGTCCCGTCCTTACGCTTTCATCACCTGTGGTGGTGACGTTTGGAACAGGCTTAATTGCCGTATCTTTGGTGAAACGGTTGCTACAGGAACAACCCCGTGTAATTGCTCACGATCACGAACATTACCCAGGTGGTTCTGAGAGCAGTTCTAGCTCTTGTGCTACCGCGCCTATTTTACGTAATCTTTCGCGAGATCAGTGCGATTCAGAGAATATTGGATGCAGTTCTAGCGCCTGTTCTCCGTCTGAAATTGTGAAAGTTACAAGGCAGGTAGTGGGAGTTGAACGTGGTCTTTACCGGGACATTTTTCAGGACAACGAAATCCCATCAGTCATGGAAGAGAAACTGCAGAAACTCCTTTACTCTGAGGGTGAGAAGATTCGAAGACGTTGCCAATTTGAAGCATCAACGATGCACTCACGCAAAGTAAAGGTTCCGGAGGTAGGTACTATCCCAGATATCCAAACTTGGTTCGATGCTACGTTTCCTGGTAACTCCGTTAGGTTTTCTGATTTCGACGGTTATACTGTTGCTACGGAGGACATTAACATGGATGTTCAGGATTGTAGACTTAAGTTCGGGAAGACTTTTCGACCTTATGAATTTAAGGAATCACTGAAACCAGTACTGAGGACAGCAATGCCAGAAAAACGACAGGGTAGTTTGATTGAAAGTGTGCTGGCCTTTCGTAAAAGAAATTTGGCTGCGCCCAGATTACAAGGAGCTTTGAATGAATGGCACACAATTGAGAATGTGCTAACGAAGGCGTTAAAGGTATTCTTCTTTGAAGATTTAATTGATCGAACGGATCACTGCACTTACGAGTCAGCGCTCAGATGGTGGGATAAACAATCAGTGACAGCTCGAGCGCAGCTCGTGGCGGATCAGCGGAGGTTATGTGATGTTGACTTCACGACTTATAACTTCATGATAAAAAATGATGTAAAGCCGAAGTTAGATCTAACACCTCAAGTTGAATATGCAGCTTTGCAGACTGTTGTATATCCTGATAAGATAGTCAATGCTTTCTTTGGTCCGATCATAAAGGAGATTAATGAACGGATCATCAGAGCGCTTAGACCTCATGTGGTCTTTAATTCTCGTATGACTGCTGATGAACTGAATGAAACAGCTGCCTTTTTGACACCTCATAAGTACAGAGCCTTAGAGATTGATTTTTCAAAATTTGATAAATCAAAGACTGGGCTTCATATCAAAGCTGTCATTGGACTCTATAAGCTCTTTGGCCTAGATGGCCTGTTAAAAGTGCTCTGGGAAAAATCGCAATATCAGACTTACGTGAAAGATAGAAACTTCGGTCTCGAGGCATATCTATTGTATCAGCAAAAGTCAGGAAATTGTGACACTTACGGTTCGAACACCTGGTCTGCCGCCTTGGCGTTGTTAGATTGTCTTCCTTTGGAAGATGCACATTTCTGTGTATTTGGTGGTGATGATTCATTGATATTGTTTGATCAGGGATACATAATTTCCGACCCATGCCGGCAACTTGCCGGTACTTGGAATCTTGAATGTAAAGTGTTCGACTTCAAGTACCCCGCATTTTGTGGTAAATTTCTGCTGTGCATAGATGGAAAATATCAATTTGTTCCAGATGCGGCAAAATTTATCACAAAATTAGGTAGAACTGATGTGAGAGATGTAGAAGTTTTGAGTGAGATTTATATCTCTATCAATGACAATTACAAATCTTACAAAGACTTTAAGGTGCTTGATGCTTTGGATAAGGCTTTAGTGGATAGATATCGATCCCCTTATAGTGCTATTTCTGCTTTGGTTTCTTTATGTTATCATATCTTTGACTTTAATAAGTTTAAGTTGCTGTTTAATTGTGAAGGGAAATTTGTGGATAAGAAGCTGAGAAAAGACTTCGAGTGGTGAACTCTAGGTCCTGATGTTTAAATCTACTGTATTTACCTTCGCATGATGGCTACTTTCTCTTGTGTGTGTTGTGGTACCTTAACTACAAGTACTTACTGTGGTAAGAGATGTGAGCGAAAGCATGTATATTCTGAAACAAGAAATAAGAGATTGGAACTTTACAAGAAGTATCTATTGGAACCGCAAAAATGCGCCCTGAATGGAATCGTTGGACACAGTTGTGGAATGCCATGCTCCATTGCGGAAGAGGCTTGTGATCAACTGCCAATCGTGAGTAGGTTCTGTGGCCAAAAGCATGCGGATCTGTATGATTCACTTCTGAAACGTTCTGAACAGGAGTTACTTCTTGAATTTCTCCAGAAGAAGATGCAGGAGCTGAAACTTTCTCATATCGTAAAAATGGCTAAGCTTGAAAGTGAGGTTAACGCAATACGTAAGTCCGTAGCTTCTTCTTTTGAAGATTCTGTTGGATGTGATGATTCTTCTTCCGTTGCTAGCTGATTAATTAAGCTCGCGCCCTGCTACCCGGgttttagagctagaaatagcaagttaaaataaggctagtccgttatcaacttgaaaaagtggcaccgagtcggtgcatcagagtggcgcagcggaagcgtggtgggcccataacccacaggtcccaggatcgaaacctggctctgataGCGGCCGCTCAGCTAGCTAAAAAAAAAAAAAAATGTTTGATCAGATCATTCAAATCTGATGGTGCCCATCAACCATATGATGGGAGTGTTTGCAAGTCCACTATAATCGAACTTGAAAACGATGCCTGAATTGGAAACCATGAATCTTAACGGACTCTGGAGAGAAAATTTAGGAATTGGTATGTAAGCTACAACTTCCGGTAGCTGCGTCACACTTTAAGAGTGTGCATACTGAGCCGAAGCTCAGCTTCGGTCCCCCAAGGGAAGACCAcgcgcgcgtcatgcaagcttggcgtaatcatggtcatagctgtttcctgtgtgaaattgttatccgctcacaattccacacaacatacgagccggaagcataaagtgtaaagcctggggtgcctaatgagtgagctaactcacattaattgcgttgcgctcactgcccgctttccagtcgggaaacctgtcgtgccagctgcattaatgaatcggccaacgcgcggggagaggcggtttgcgtattgggcgctcttccgcttcctcgctcactgactcgctgcgctcggtcgttcggctgcggcgagcggtatcagctcactcaaaggcggtaatacggttatccacagaatcaggggataacgcaggaaagaacatgtgagcaaaaggccagcaaaaggccaggaaccgtaaaaaggccgcgttgctggcgtttttccataggctccgcccccctgacgagcatcacaaaaatcgacgctcaagtcagaggtggcgaaacccgacaggactataaagataccaggcgtttccccctggaagctccctcgtgcgctctcctgttccgaccctgccgcttaccggatacctgtccgcctttctcccttcgggaagcgtggcgctttctcatagctcacgctgtaggtatctcagttcggtgtaggtcgttcgctccaagctgggctgtgtgcacgaaccccccgttcagcccgaccgctgcgccttatccggtaactatcgtcttgagtccaac

pBSMVγGW2T2Trna^Ile^ (γGW2T2Ile for short)

cgttcagcccgaccgctgcgccttatccggtaactatcgtcttgagtccaacccggtaagacacgacttatcgccactggcagcagccactggtaacaggattagcagagcgaggtatgtaggcggtgctacagagttcttgaagtggtggcctaactacggctacactagaagaacagtatttggtatctgcgctctgctgaagccagttaccttcggaaaaagagttggtagctcttgatccggcaaacaaaccaccgctggtagcggtggtttttttgtttgcaagcagcagattacgcgcagaaaaaaaggatctcaagaagatcctttgatcttttctacggggtctgacgctcagtggaacgaaaactcacgttaagggattttggtcatgagattatcaaaaaggatcttcacctagatccttttaaattaaaaatgaagttttaaatcaatctaaagtatatatgagtaaacttggtctgacagttaccaatgcttaatcagtgaggcacctatctcagcgatctgtctatttcgttcatccatagttgcctgactccccgtcgtgtagataactacgatacgggagggcttaccatctggccccagtgctgcaatgataccgcgagacccacgctcaccggctccagatttatcagcaataaaccagccagccggaagggccgagcgcagaagtggtcctgcaactttatccgcctccatccagtctattaattgttgccgggaagctagagtaagtagttcgccagttaatagtttgcgcaacgttgttgccattgctacaggcatcgtggtgtcacgctcgtcgtttggtatggcttcattcagctccggttcccaacgatcaaggcgagttacatgatcccccatgttgtgcaaaaaagcggttagctccttcggtcctccgatcgttgtcagaagtaagttggccgcagtgttatcactcatggttatggcagcactgcataattctcttactgtcatgccatccgtaagatgcttttctgtgactggtgagtactcaaccaagtcattctgagaatagtgtatgcggcgaccgagttgctcttgcccggcgtcaatacgggataataccgcgccacatagcagaactttaaaagtgctcatcattggaaaacgttcttcggggcgaaaactctcaaggatcttaccgctgttgagatccagttcgatgtaacccactcgtgcacccaactgatcttcagcatcttttactttcaccagcgtttctgggtgagcaaaaacaggaaggcaaaatgccgcaaaaaagggaataagggcgacacggaaatgttgaatactcatactcttcctttttcaatattattgaagcatttatcagggttattgtctcatgagcggatacatatttgaatgtatttagaaaaataaacaaataggggttccgcgcacatttccccgaaaagtgccacctaaattgtaagcgttaatattttgttaaaattcgcgttaaatttttgttaaatcagctcattttttaaccaataggccgaaatcggcaaaatcccttataaatcaaaagaatagaccgagatagggttgagtgttgttccagtttggaacaagagtccactattaaagaacgtggactccaacgtcaaagggcgaaaaaccgtctatcagggcgatggcccactacgtgaaccatcaccctaatcaagttttttggggtcgaggtgccgtaaagcactaaatcggaaccctaaagggagcccccgatttagagcttgacggggaaagccggcgaacgtggcgagaaaggaagggaagaaagcgaaaggagcgggcgctagggcgctggcaagtgtagcggtcacgctgcgcgtaaccaccacacccgccgcgcttaatgcgccgctacagggcgcgtcccattcgccattcaggctgcgcaactgttgggaagggcgatcggtgcgggcctcttcgctattacgccagctggcgaaagggggatgtgctgcaaggcgattaagttgggtaacgccagggttttcccagtcacgacgttgtaaaacgacggccagtgaattaatacgactcactataGTATAGCTTGAGCATTACCGTCGTGTAATTGCAACACTTGGCTTGCCAAATAACGCTAAAGCGTTCACGAAACAAACAACACTTCGGCATGGATGTTGTGAAGAAATTCGCCGTCATGTCAGTGACTGTAGTAGCAGGTCCCGTCCTTACGCTTTCATCACCTGTGGTGGTGACGTTTGGAACAGGCTTAATTGCCGTATCTTTGGTGAAACGGTTGCTACAGGAACAACCCCGTGTAATTGCTCACGATCACGAACATTACCCAGGTGGTTCTGAGAGCAGTTCTAGCTCTTGTGCTACCGCGCCTATTTTACGTAATCTTTCGCGAGATCAGTGCGATTCAGAGAATATTGGATGCAGTTCTAGCGCCTGTTCTCCGTCTGAAATTGTGAAAGTTACAAGGCAGGTAGTGGGAGTTGAACGTGGTCTTTACCGGGACATTTTTCAGGACAACGAAATCCCATCAGTCATGGAAGAGAAACTGCAGAAACTCCTTTACTCTGAGGGTGAGAAGATTCGAAGACGTTGCCAATTTGAAGCATCAACGATGCACTCACGCAAAGTAAAGGTTCCGGAGGTAGGTACTATCCCAGATATCCAAACTTGGTTCGATGCTACGTTTCCTGGTAACTCCGTTAGGTTTTCTGATTTCGACGGTTATACTGTTGCTACGGAGGACATTAACATGGATGTTCAGGATTGTAGACTTAAGTTCGGGAAGACTTTTCGACCTTATGAATTTAAGGAATCACTGAAACCAGTACTGAGGACAGCAATGCCAGAAAAACGACAGGGTAGTTTGATTGAAAGTGTGCTGGCCTTTCGTAAAAGAAATTTGGCTGCGCCCAGATTACAAGGAGCTTTGAATGAATGGCACACAATTGAGAATGTGCTAACGAAGGCGTTAAAGGTATTCTTCTTTGAAGATTTAATTGATCGAACGGATCACTGCACTTACGAGTCAGCGCTCAGATGGTGGGATAAACAATCAGTGACAGCTCGAGCGCAGCTCGTGGCGGATCAGCGGAGGTTATGTGATGTTGACTTCACGACTTATAACTTCATGATAAAAAATGATGTAAAGCCGAAGTTAGATCTAACACCTCAAGTTGAATATGCAGCTTTGCAGACTGTTGTATATCCTGATAAGATAGTCAATGCTTTCTTTGGTCCGATCATAAAGGAGATTAATGAACGGATCATCAGAGCGCTTAGACCTCATGTGGTCTTTAATTCTCGTATGACTGCTGATGAACTGAATGAAACAGCTGCCTTTTTGACACCTCATAAGTACAGAGCCTTAGAGATTGATTTTTCAAAATTTGATAAATCAAAGACTGGGCTTCATATCAAAGCTGTCATTGGACTCTATAAGCTCTTTGGCCTAGATGGCCTGTTAAAAGTGCTCTGGGAAAAATCGCAATATCAGACTTACGTGAAAGATAGAAACTTCGGTCTCGAGGCATATCTATTGTATCAGCAAAAGTCAGGAAATTGTGACACTTACGGTTCGAACACCTGGTCTGCCGCCTTGGCGTTGTTAGATTGTCTTCCTTTGGAAGATGCACATTTCTGTGTATTTGGTGGTGATGATTCATTGATATTGTTTGATCAGGGATACATAATTTCCGACCCATGCCGGCAACTTGCCGGTACTTGGAATCTTGAATGTAAAGTGTTCGACTTCAAGTACCCCGCATTTTGTGGTAAATTTCTGCTGTGCATAGATGGAAAATATCAATTTGTTCCAGATGCGGCAAAATTTATCACAAAATTAGGTAGAACTGATGTGAGAGATGTAGAAGTTTTGAGTGAGATTTATATCTCTATCAATGACAATTACAAATCTTACAAAGACTTTAAGGTGCTTGATGCTTTGGATAAGGCTTTAGTGGATAGATATCGATCCCCTTATAGTGCTATTTCTGCTTTGGTTTCTTTATGTTATCATATCTTTGACTTTAATAAGTTTAAGTTGCTGTTTAATTGTGAAGGGAAATTTGTGGATAAGAAGCTGAGAAAAGACTTCGAGTGGTGAACTCTAGGTCCTGATGTTTAAATCTACTGTATTTACCTTCGCATGATGGCTACTTTCTCTTGTGTGTGTTGTGGTACCTTAACTACAAGTACTTACTGTGGTAAGAGATGTGAGCGAAAGCATGTATATTCTGAAACAAGAAATAAGAGATTGGAACTTTACAAGAAGTATCTATTGGAACCGCAAAAATGCGCCCTGAATGGAATCGTTGGACACAGTTGTGGAATGCCATGCTCCATTGCGGAAGAGGCTTGTGATCAACTGCCAATCGTGAGTAGGTTCTGTGGCCAAAAGCATGCGGATCTGTATGATTCACTTCTGAAACGTTCTGAACAGGAGTTACTTCTTGAATTTCTCCAGAAGAAGATGCAGGAGCTGAAACTTTCTCATATCGTAAAAATGGCTAAGCTTGAAAGTGAGGTTAACGCAATACGTAAGTCCGTAGCTTCTTCTTTTGAAGATTCTGTTGGATGTGATGATTCTTCTTCCGTTGCTAGCTGATTAATTAAGCTCGCGCCCTGCTACCCGGgttttagagctagaaatagcaagttaaaataaggctagtccgttatcaacttgaaaaagtggcaccgagtcggtgcatcagagtggcgcagcggaagcgtggtgggcccataacccacaggtcccaggatcgaaacctggctctgataGCGGCCGCTCAGCTAGCTAAAAAAAAAAAAAAATGTTTGATCAGATCATTCAAATCTGATGGTGCCCATCAACCATATGATGGGAGTGTTTGCAAGTCCACTATAATCGAACTTGAAAACGATGCCTGAATTGGAAACCATGAATCTTAACGGACTCTGGAGAGAAAATTTAGGAATTGGTATGTAAGCTACAACTTCCGGTAGCTGCGTCACACTTTAAGAGTGTGCATACTGAGCCGAAGCTCAGCTTCGGTCCCCCAAGGGAAGACCAcgcgcgcgtcatgcaagcttggcgtaatcatggtcatagctgtttcctgtgtgaaattgttatccgctcacaattccacacaacatacgagccggaagcataaagtgtaaagcctggggtgcctaatgagtgagctaactcacattaattgcgttgcgctcactgcccgctttccagtcgggaaacctgtcgtgccagctgcattaatgaatcggccaacgcgcggggagaggcggtttgcgtattgggcgctcttccgcttcctcgctcactgactcgctgcgctcggtcgttcggctgcggcgagcggtatcagctcactcaaaggcggtaatacggttatccacagaatcaggggataacgcaggaaagaacatgtgagcaaaaggccagcaaaaggccaggaaccgtaaaaaggccgcgttgctggcgtttttccataggctccgcccccctgacgagcatcacaaaaatcgacgctcaagtcagaggtggcgaaacccgacaggactataaagataccaggcgtttccccctggaagctccctcgtgcgctctcctgttccgaccctgccgcttaccggatacctgtccgcctttctcccttcgggaagcgtggcgctttctcatagctcacgctgtaggtatctcagttcggtgtaggtcgttcgctccaagctgggctgtgtgcacgaaccccccgttcagcccgaccgctgcgccttatccggtaactatcgtcttgagtccaac

**Figure S1.** The sequences of Barley Stripe Mosaic Virus (BSMV) plasmids used in the study. The plasmid backbones are shown in black lower-case letters. The BSMV genomic sequences are shown in black uppercase letters. The *PDS* gene coding sequence fragment is shown in italic black lowercase letters. The spacers of sgRNA are shown in red uppercase letters, while the scaffold of sgRNA is shown in blue lowercase letters. The mobile RNAs are shown in red lowercase letters. The *Not*I and *Pac*I cut sites used for sgRNA and sgRNA-Mobile RNA subcloning are underlined. The enzyme cut sites for plasmid linearization before *in vitro* transcription are highlighted by yellow color.

**Figure S2.** The expression level of Cas9 in transgenic plants. The quantitative PCR of Cas9 was conducted using Cas9F and Cas9seq1 (Table S1). The *TaActin* gene was adopted as reference. The relative expression level of Cas9 is shown as means ± SE based. The T3 progeny of line 7438 and line C413 were used, the T4 progeny of line 707 were used. The biological replicate number for line 7438, C413 and 707 are 6, 9 and 4, respectively. Three biological replicates were used for cv. Bobwhite in both bar plots.

**Figure S3.** Somatic editing induced by the BSMV- sgRNA constructs with and without mobile RNA in the non-inoculated 4th leaf of the C413 line. The mutation frequency at the GW2T2 target was calculated separately for each of the three genomes.

**Figure S4.** Relationship between the mutagenesis ratio in the M1 progeny of plants inoculated by BSMV-GW2T2 (a, b, and d) or BSMV-GW2T2 (c and e) and the somatic editing efficiency evaluated in the 6^th^ (a), 4^th^ (b and c), and 2^nd^ (d and e) leaf. The 2^nd^ and 4^th^ leaf were sampled at 4-leaf stage. The 6^th^ leaf was sampled at 6-leaf stage. Each data point represents an individual plant.

**Figure S5.** The somatic editing efficiency of each target site in the 4^th^ leaf (developed after inoculation) of plants inoculated by multiplex editing pool (BSMV-GUG) or BSMV carrying single guide RNAs. From left to right, the three box-whisker plots show the editing efficiency of GW2T2, UPL3T11 and GW7T6, respectively. The data from all three genomes are separated to calculate the somatic editing efficiency for all three targets.

10 20 30 40 50 60 70 80

....|....|....|....|....|....|....|....|....|....|....|....|....|....|....|....|

pQ_Chr5A CAGACACAGGCCGGAGGGGGCGTTCGGGCCGGCACGTACCCCCGCCCCACGTCCCGATCACCGGGTCGCCTCACCCTCAC

pQ_Chr5B ---................T.......C........C.--------..........C...........------......

pQ_Chr5D ---..G.............T.......C........C................G--------------------.....-

90 100 110 120 130 140 150 160

....|....|....|....|....|....|....|....|....|....|....|....|....|....|....|....|

pQ_Chr5A GGGCTCGCTCTCAACCGGTCAACGTACTGTACCGCACCGGTGCAGCCCATTTACGGCGCTGTCGCGGTGTCGCCGTCCTC

pQ_Chr5B ...............G..C.........................................--------............

pQ_Chr5D --------------------------------...................................C..........--

170 180 190 200 210 220 230 240

....|....|....|....|....|....|....|....|....|....|....|....|....|....|....|....|

pQ_Chr5A CCTCCGTCCGTCCATTCCATCGGGTCCTCCCGTGGCCGTGCCGAGCCCCCACGCGGTGCCGTGACCGCGACCGTACGCGA

pQ_Chr5B .G..............-............------....................C.....C..................

pQ_Chr5D --................G.........G..A..T................A.........C..................

250 260 270 280 290 300 310 320

....|....|....|....|....|....|....|....|....|....|....|....|....|....|....|....|

pQ_Chr5A GCCCGCCCGCCCGGCCGGCCCGTGACCACGAGCGTAAG--GTTACGAGGATGCTACATACATGCGCGTACGGCTTACGTA

pQ_Chr5B .G...----------T.A.AA.C....CATC.....C.CTT....C.....-----......A....C.....G.....-

pQ_Chr5D ------------------.AA.C....CATC....G..CTT....C...-------......A..........G.....-

**pQT17checkF**

330 340 350 360 370 380 390 400

....|....|....|....|....|....|....|....|....|....|....|....|....|....|....|....|

pQ_Chr5A GACAGAGCGAGTATACATGGCAGACTTTGGCGCGGGTG-----GGCGTCT----CTCGGGTGGTGGGGGATGCATTTGTC

pQ_Chr5B ---------------------..GT.............GCGTA.C.--------.........................G

pQ_Chr5D ---------------------..GT.............GCGTA.....T.CTAC.........................G

410 420 430 440 450 460 470 480

....|....|....|....|....|....|....|....|....|....|....|....|....|....|....|....|

pQ_Chr5A GAAAGGAACAACGTTTCCGATGGGGCGAGCGGGGGCGTGACCGGTCGACCGGAACGGCACAGTGGCCCGGACAGGTACGC

pQ_Chr5B ...............GG..........G...TAC.----------...................C....A.....-----

pQ_Chr5D ...............G...........GA.-------C.G........................C...............

**pQT18checkF** **pQT17 pQT17checkR**

490 500 510 520 530 540 550 560

....|....|....|....|....|....|....|....|....|....|....|....|....|....|....|....|

pQ_Chr5A CTGTGGCTGCGTACCCGCCCTCGCGTGGG--TCTACGTCGAATAAT-------TGCACCCATCCCATTACACCCGGGCCC

pQ_Chr5B --...............----..T.....TC...C...........-------...............T...........

pQ_Chr5D .................----..T.....TC...C...........TGCACCC.......G...................

**pQT18 pQT23checkF**

570 580 590 600 610 620 630 640

....|....|....|....|....|....|....|....|....|....|....|....|....|....|....|....|

pQ_Chr5A CG----------GCGAGCAAAACAGTACCCGGACCTAGCCTGCAACCCCCAAGCCC-------------CC---------

pQ_Chr5B ..CGCCCCCCGACGAGAA.....C......A.C...CA..................AGTAGTACCACCA..ACCGATGTG

pQ_Chr5D ..-----------------------------A...............T........AGTACCACCAGCA..---------

650 660 670 680 690 700 710 720

....|....|....|....|....|....|....|....|....|....|....|....|....|....|....|....|

pQ_Chr5A GACGCGGTACATGCACGCATACACACACACGAAAGAGAGCGGTGCACGCAGTACGT-ACACACCGGCAGGCG--------

pQ_Chr5B ..T.T.A......................T....A.............AG....A.A.A.TC...T...A..CTGCTGCC

pQ_Chr5D ..T.T................TG......T....A.............AG....A.-----........A..CTGCTGCG

**pQT18checkR**

730 740 750 760 770 780 790 800

....|....|....|....|....|....|....|....|....|....|....|....|....|....|....|....|

pQ_Chr5A --GACGCGGTACGTGCTAGGCTAGGCCAGGCTAGATTGGTCCAGCTGCCGGCTCCCCC------------CGTGCTCTCG

pQ_Chr5B AT....-.......A..----CCTA.T.......GC....................G.CTCGCCAACCGG.TC.......

pQ_Chr5D CG....-.......A..-----....T...A...G.....................G.CTCGCCAACCGG.TC.......

**pQT23**

810 820 830 840 850 860 870 880

....|....|....|....|....|....|....|....|....|....|....|....|....|....|....|....|

pQ_Chr5A TCGGCACGGCGCG---ACATGCCGTCACGTACCTG--CTCCGCCTGTGGCCCTTGGCGCTTGCG----------CCCGGC

pQ_Chr5B ............A--------------..C....TAC..G....GC............T.....TAGCGGCGCT------

pQ_Chr5D ............ACGC..................TAC..G....GT............T.....TAGCGGCGCT......

890 900 910 920 930 940 950 960

....|....|....|....|....|....|....|....|....|....|....|....|....|....|....|....|

pQ_Chr5A CGGCCGCGGTCGGGTCAACACACGAGGCCTCCCAGATCGGGCGCG------------GCATGCATGTGCCGCCGG-----

pQ_Chr5B ----------------------------------------------------------.................AGCAA

pQ_Chr5D ......T....AA.GG.GGC.T..CAA--G...............GCATGCATCCAT..................AGCAA

**pQT23checkR**

970 980 990 1000 1010 1020 1030 1040

....|....|....|....|....|....|....|....|....|....|....|....|....|....|....|....|

pQ_Chr5A ------------------------------TACGTATGTACGTATACCGGCGCGGATTAATTTAGAGTTCGATTTGATTA

pQ_Chr5B GCGCCGGTACGTAACGTATAGTACTATGTA....C.C....A....G......T......-......-----........

pQ_Chr5D GCGCCGGTACGTAACGTATAGTACTATGTA..T...C....A....G.............-......-----.G......

1050 1060 1070 1080 1090 1100 1110 1120

....|....|....|....|....|....|....|....|....|....|....|....|....|....|....|....|

pQ_Chr5A GAGGGAGGG-----------------------------------------------------------------------

pQ_Chr5B ..C....A.CATGCTTGGATACGTTTTAGTCCCATGACTAAAAGTAGTGGGACTAGAACTTGCTAGTCTCACCCATGCTT

pQ_Chr5D ..C......-----------------------------------------------------------------------

1130 1140 1150 1160 1170 1180 1190 1200

....|....|....|....|....|....|....|....|....|....|....|....|....|....|....|....|

pQ_Chr5A --------------------------------------------------------------------------------

pQ_Chr5B GGATCCAAATACTAAAGAGACTAAAATCTAGTTATTGAGCATTTATTATCCTCCAAACCCTTCAATCCAGAACTAAGGAG

pQ_Chr5D --------------------------------------------------------------------------------

1210 1220 1230 1240 1250 1260 1270 1280

....|....|....|....|....|....|....|....|....|....|....|....|....|....|....|....|

pQ_Chr5A --------------------------------------------------------------------------------

pQ_Chr5B AGGAATTAAATGAGGAGAGAGAGAGCTAATACATATTTTAATAGGTTTCCCATGACTAAAAGATTTTAGTCTCAAGACTA

pQ_Chr5D --------------------------------------------------------------------------------

1290 1300 1310 1320 1330 1340 1350 1360

....|....|....|....|....|....|....|....|....|....|....|....|....|....|....|....|

pQ_Chr5A --------------------------------------------------------------------------------

pQ_Chr5B GTCCTAGCCTCTCTTTAGTCAGGGGTGCTTGGAACTTTAGCCTTTAAAAGAGACTATTTTTAGTCAGACTAAAAATAGTC

pQ_Chr5D --------------------------------------------------------------------------------

1370 1380 1390 1400 1410 1420 1430 1440

....|....|....|....|....|....|....|....|....|....|....|....|....|....|....|....|

pQ_Chr5A ----------------------------AGGGGGGCGTGCGGTGCACTTGGGCAATGTAATGCGGTCCTGCGAGGAGGGA

pQ_Chr5B CCTTGTATCCAAGCACCCTCGGAGTCACG.A.........A...........T.G......................C..

pQ_Chr5D ----------------------------------......A........-..T........................C..

**pQT25checkF**

1450 1460 1470 1480 1490 1500 1510 1520

....|....|....|....|....|....|....|....|....|....|....|....|....|....|....|....|

pQ_Chr5A TC---TCATCTAACCTAGCAGCACAGGGCGTACGGCCGGGGGCTTATCTTACTCTCGCTAGGTGCCTAAGATAACCAGCA

pQ_Chr5B ..TAT.A...G--------...GT...A..........................G..A...A..G....A....-..A..

pQ_Chr5D ..---.A...G--------....T...A....................................G....-....-..A..

**pQT25**

1530 1540 1550 1560 1570 1580 1590 1600

....|....|....|....|....|....|....|....|....|....|....|....|....|....|....|....|

pQ_Chr5A TGAGTTGATGGTGCCGGCCGTTAACAATTCCAACCAAAAGCTAATCGTCTGGTGCACACGCAATGGTGGACACTATCATA

pQ_Chr5B ............................G......G.........T.....C.....T.A.CG................G

pQ_Chr5D ........................T......................C...........A.CG....C...........G

1610 1620 1630 1640 1650 1660 1670 1680

....|....|....|....|....|....|....|....|....|....|....|....|....|....|....|....|

pQ_Chr5A CCATGGATCACGTGGGTGGTTCTTTGTCCAT---------GCCACTGGTTTTTCCT---------CTTTCGCTAGAGTTA

pQ_Chr5B ..........--..........C.G..TG..AACTG----.T..GA.......TT.TTCCTTCTA....A.T.C......

pQ_Chr5D ......G...--..........C.G..TG..AATTGGTCA.T..G.......CTT.---CTTCTT....G.T.CA.....

**pQT25checkR**

1690 1700 1710 1720 1730 1740 1750 1760

....|....|....|....|....|....|....|....|....|....|....|....|....|....|....|....|

pQ_Chr5A AT------TAAGCGAATGGCTTTTGAATCCGTGTTTGTTCA----TGTCGGTCAAATCAAATCTCATGAATATGTT---G

pQ_Chr5B .CAGA---CC.T.A.GG............TTGC.CCA.CTGCGTC..................CT.G..CA.A...GAG.

pQ_Chr5D .CAAACATC..TTA.TC........G...TCG..CCACC.GTGTC...................T.G..CA.A...GTG.

1770 1780 1790 1800 1810 1820 1830 1840

....|....|....|....|....|....|....|....|....|....|....|....|....|....|....|....|

pQ_Chr5A TACCGTTTGTCCTA-GATGGAGCTGGATTAAATAA-TTTCGGCA-TTTGTGGGTGCCCCTCCATACGATGTACTGGAGAG

pQ_Chr5B ....A.....T.C.CA..A.G....A.C....C.GC....TT.GT......A...TT.............C..C.T.A.A

pQ_Chr5D ....A.....T...CA..A.G....A.....G...C....TT..-...A..A...TT...G.G.......C.TCAT.A..

1850 1860 1870 1880 1890 1900 1910 1920

....|....|....|....|....|....|....|....|....|....|....|....|....|....|....|....|

pQ_Chr5A G-------------------------------GTTTTAAAACATT-----------------------------TTGTTG

pQ_Chr5B A-AAATTAGTATGTTTTCTTGGATGAAAGATTT.....GT.....TTCTTGGAGGAAAGCTTTTTTTTAGTACG......

pQ_Chr5D ATTTTTTAGTACGTTTTTCTCGAGGAAAGGT-T.....GT.....TTCTTGGAGGAAAGGTTTTTTTTAGTACG......

1930 1940 1950 1960 1970 1980 1990 2000

....|....|....|....|....|....|....|....|....|....|....|....|....|....|....|....|

pQ_Chr5A CTTAGCGTTTGGCTAG------------------CGATGTAATAACAAAATAGTGTCATGAC-------ATCTGCCACAA

pQ_Chr5B G.....A.....T.G.A--------TCGACGACA..G.....---A....C.A.T.......ATTGAAG.......G...

pQ_Chr5D G.....A.C...T.G.ATCGATGTGTCGATGACAT.G.....-.CA....C.A.T.......ATCGAAG......TG...

2010 2020 2030 2040 2050 2060 2070 2080

....|....|....|....|....|....|....|....|....|....|....|....|....|....|....|....|

pQ_Chr5A AGGTGCGTCGACATCCATACTTTACTC-TTCGTTGCACTACAAACATATTTTGTGATGTACGCTCCGTGTGATATATATA

pQ_Chr5B ....A.A...G.T...G....C.....-..T......T.GG..........----.....GTACTT.G.C.G.T.T....

pQ_Chr5D ....A.......G...G........C.T...T.....T.GG..........----.....GTACTT.G.C.G.T.T....

2090 2100 2110 2120 2130 2140 2150 2160

....|....|....|....|....|....|....|....|....|....|....|....|....|....|....|....|

pQ_Chr5A CTTGTATCTTTAGAAAGGCCTAGAGTCTTAGGTGTAAGACCTTTCTAGCTGCAGGCCTGATTCGCACAAGGTGCTACTTG

pQ_Chr5B T...C.....CGAG..C.T.C........C..CAA....................A........................

pQ_Chr5D ....C.....CGAG..C.T.C........C..CA...C..........T..................G............

2170 2180 2190 2200 2210 2220 2230 2240

....|....|....|....|....|....|....|....|....|....|....|....|....|....|....|....|

pQ_Chr5A GTTGTTGCATATAAAAAACGGTAATACTATAATACTCTGTCCCTCCGGGACTAAATTACTTAGCAAGTTGACATTTGTTT

pQ_Chr5B ......TT........TG--...............C......TGAT..............................A...

pQ_Chr5D ......TT........TGATA..............C.....TTGAT...........................C..A...

2250 2260 2270 2280 2290 2300 2310 2320

....|....|....|....|....|....|....|....|....|....|....|....|....|....|....|....|

pQ_Chr5A ATATTTAGCTTCGGGAACGTCCAACGTCTCCGGTGTAAGACCTTTCTAGTCGCATTCCTGATTCAAACAAGATGCTACTT

pQ_Chr5B ..G........GA...G............TT.......................CAT.......................

pQ_Chr5D ..G........GA................T...................C.....AT.................G.....

2330 2340 2350 2360 2370 2380 2390 2400

....|....|....|....|....|....|....|....|....|....|....|....|....|....|....|....|

pQ_Chr5A GGTTGTATAT-AAAATGATTGGGCTATAATACTCCTCTTCGCAACAATTAAATTACTCAGTAGGTGAACATTTTATATAT

pQ_Chr5B .A..C.....-....CA...ATAT..........A........GTG............T...AC.TG...........C.

pQ_Chr5D .A..C.....A....CA.A..TAT..........A...C...................T...AC.TG.T.........C.

2410 2420 2430 2440 2450 2460 2470 2480

....|....|....|....|....|....|....|....|....|....|....|....|....|....|....|....|

pQ_Chr5A TCAACTCTAGCATATAATA-CAAATTTGTTGTCGGCATAGAAATGAGTTGCAATCATTTCATAAATAGGAAGAACACATG

pQ_Chr5B .T.G..T.G..C.C...C.C....CCATA.....A.........C................-CG.G..A..........C

pQ_Chr5D .T.GT.T.G....C.....C..G.CCA.AC....A.................---......---.G..AT..........

2490 2500 2510 2520 2530 2540 2550 2560

....|....|....|....|....|....|....|....|....|....|....|....|....|....|....|....|

pQ_Chr5A GCTTAT----------------------ACCAAACTAGCAGCCTAAAAAGGTTTTTT-------TTTCTTCTTTCTGAGA

pQ_Chr5B AT....-------------------------.GGTC.TTTAT..C.-----.....CTCAAACG...T..T..AT...A.

pQ_Chr5D .T..G.TCCAAACTCGAGCTCTAGATCG.................T.----.A...CTGAGTCA...T.A.C..T..TA.

2570 2580 2590 2600 2610 2620 2630 2640

....|....|....|....|....|....|....|....|....|....|....|....|....|....|....|....|

pQ_Chr5A GGAGGCATTTAGCTTGTGGAGCAAAATGTTGAA-GCGGCT----GGGC---GAAAAAACCCTCGGCTGATCCGCGTGAGG

pQ_Chr5B ...........C.....................G......GTCC....CAAA......AA.....T........C..G.A

pQ_Chr5D A..A------------------...........G......GTCC....C--A......A......T........C.CG.A

**pQT26checkF**

2650 2660 2670 2680 2690 2700 2710 2720

....|....|....|....|....|....|....|....|....|....|....|....|....|....|....|....|

pQ_Chr5A GCACGACACGTGGCGTCCCGGTCCACGGGGTGTGTGGCCGTAGCGATTAGCGAGGCTCCGCTGCACAAAAATAGTTTACC

pQ_Chr5B .GG................CA...GG...........G...............A.................A........

pQ_Chr5D .GG................CA...G............G...............A..........................

**pQT26**

2730 2740 2750 2760 2770 2780 2790 2800

....|....|....|....|....|....|....|....|....|....|....|....|....|....|....|....|

pQ_Chr5A TCTGATGCCCTTGGCCTCCCCGACGTCCCATCTCG----CTTTCTCTCTTTCTCTTTCTCCCACTGGCCT----GGCCCC

pQ_Chr5B .G............G....T...............----......T........................GGGA......

pQ_Chr5D ...................................CTTT..........C....................----......

**pQT26checkR**

2810 2820 2830 2840 2850 2860 2870 2880

....|....|....|....|....|....|....|....|....|....|....|....|....|....|....|....|

pQ_Chr5A CTCTCCTCGTCGTCCTCCAGTCCTCAT-CCCCCGCCCCATGGCCACCCCCACCACGGCC-GCCCCCCCCCCCCCTCCCCC

pQ_Chr5B ...........................C...................A.......C...TC.T.....T...........

pQ_Chr5D ...........................C...................A.......C...------T..T...........

2890 2900 2910 2920 2930 2940 2950 2960

....|....|....|....|....|....|....|....|....|....|....|....|....|....|....|....|

pQ_Chr5A CACCACTACTTCTACTCCCCCCGCCCCGCCCTCGCAGCCCGCGGCCACCGCGCTCCCATGCCATAGACGCGACCCCACTC

pQ_Chr5B T........................TT.....................................................

pQ_Chr5D T........................T....................G.................................

2970 2980 2990 3000 3010 3020 3030 3040

....|....|....|....|....|....|....|....|....|....|....|....|....|....|....|....|

pQ_Chr5A ATCGGTCCAGGTCGGTCGCTCGGAGCCGAGCGGCGGCGGGCGGGCGAG------GAGTGCGTTTTATTCGGTCCCGGCGG

pQ_Chr5B .........----........C......................GCG.---GTCA..C.A....CGGAG.CG........

pQ_Chr5D .......G.............C...G..............A.A.GCG.TGGGTCA..C.A....CGGAG.CG........

3050

....|....|....

pQ_Chr5A GCCTCGGATCGGAG

pQ_Chr5B ...C..........

pQ_Chr5D ..............

**Figure S6.** The alignment of the *Q* gene promoter region from the A, B and D genomes along with the CRISPR-Cas9 targets. The sequences from the Chinese Spring TGACv1 were used to create alignment. The CRISPR-Cas9 targets are marked with red arrows, which shows the orientation of each target relative to PAMs. The checking primers of each target site are indicated by blue arrows.

TAAATCATGGTAGTTACATCTCGTCATATTCTATGAACAATCATATTTCTTATGAAATCTTGCACAACTATCACGCCATAGCATGACCGACACATAACTA 100

TAAATCATGGTAGTTACATCTCGTCATATTCTATGAACAATCATATTTCTTATGAAATCTTGCACAACTATCACGCCATAGCATGACCGACACATAACTA 100

TAAGACACTATTTTTCTTTCAAAAGCTTGATAGTACTTTCTTCAAAAGCTCATACTACTACTAAACACGAGCAAGTCTTGACCAAGATGAAGCGCGCGCG 200

TAAGACACTATTTTTCTTTCAAAAGCTTGATAGTACTTTCTTCAAAAGCTCATACTACTACTAAACACGAGCAAGTCTTGACCAAGATGAAGCGCGCGCG 200

GGTGTAGCAAGGCTCCGTTTTCGTAGTAAAAACCATCTCCTTTTCTCGTTCCATCTTTTCTCAACCCGTCAAATCAAGCGAAAGCAATACTAGAACAGCG 300

GGTGTAGCAAGGCTCCGTTTTCGTAGTAAAAACCATCTCCTTTTCTCGTTCCATCTTTTCTCAACCCGTCAAATCAAGCGAAAGCAATACTAGAACAG-- 298

CTGCTATACACACGATGGTGTGTGGACGATTCCTGAACGATAACGCAGATCAACTCATCCATGCACAACAGTAAAGTGAAGCAGACGGCTGGATTTGGCC 400

---------------------------------------------------------------------------------------------------- 298

GTCGTCTCCTTGTCGTGCACGCAGTATCGTCTTTACAGCAGTTTCGATACTAGAACAGAACCATGTGCTCCAGTACGCAACAAGCGAAGCCATCGCGACA 500

----------------------------------------------------------AACCATGTGCTCCAGTACGCAACAAGCGAAGCCATCGCGACA 340

AAAACAAAATGAAGAAAAAGAACGGTGCGCTCCACCTCCCCCTCCGAGGCTCCGACCCGCCCGCCCGCAGCTCAGCTCAAGGCGCGCACACAGACACAGG 600

AAAACAAAATGAAGAAAAAGAACGGTGCGCTCCACCTCCCCCTCCGAGGCTCCGACCCGCCCGCCCGCAGCTCAGCTCAAGGCGCGCACACAGACACAGG 440

CCGGAGGGGGCGTTCGGGCCGGCACGTACCCCCGCCCCACGTCCCGATCACCGGGTCGCCTCACCCTCACGGGCTCGCTCTCAACCGGTCAACGTACTGT 700

CCGGAGGGGGCGTTCGGGCCGGCACGTACCCCCGCCCCACGTCCCGATCACCGGGTCGCCTCACCCTCACGGGCTCGCTCTCAACCGGTCAACGTACTGT 540

ACCGCACCGGTGCAGCCCATTTACGGCGCTGTCGCGGTGTCGCCGTCCTCCCTCCGTCCGTCCATTCCATCGGGTCCTCCCGTGGCCGTGCCGAGCCCCC 800

ACCGCACCGGTGCAGCCCATTTACGGCGCTGTCGCGGTGTCGCCGTCCTCCCTCCGTCCGTCCATTCCATCGGGTCCTCCCGTGGCCGTGCCGAGCCCCC 640

ACGCGGTGCCGTGACCGCGACCGTACGCGAGCCCGCCCGCCCGGCCGGCCCGTGACCACGAGCGTAAGGTTACGAGGATGCTACATACATGCGCGTACGG 900

ACGCGGTGCCGTGACCGCGACCGTACGCGAGCCCGCCCGCCCGGCCGGCCCGTGACCACGAGCGTAAGGTTACGAGGATGCTACATACATGCGCGTACGG 740

CTTACGTAGACAGAGCGAGTATACATGGCAGACTTTGGCGCGGGTGGGCGTCTCTCGGGTGGTGGGGGATGCATTTGTCGAAAGGAACAACGTTTCCGAT 1000

CTTACGTAGACAGAGCGAGTATACATGGCAGACTTTGGCGCGGGTGGGCGTCTCTCGGGTGGTGGGGGATGCATTTGTCGAAAGGAACAACGTTTCCGAT 840

GGGGCGAGCGGGGGCGTGACCGGTCGACCGGAACGGCACAGTGGCCCGGACAGGTACGCCTGTGGCTGCGTACCCGCCCTCGCGTGGGTCTACGTCGAAT 1100

GGGGCGAGCGGGGGCGTGACCGGTCGACCGGAACGGCACAGTGGCCCGGACAGGTACGCCTGTGGCTGCGTACCCGCCCTCGCGTGGGTCTACGTCGAAT 940

AATTGCAC-------------------------------------------------------------------------------------------- 1108

AATTGCACGAGCGCTGCTATGCACACGACGCATAGCAGACGATTTGTGCACGATAGGGCATATCCGTGCGTCCGTGCATATTATCAAAGTGCAGCAGACG 1040

--------------------------------------------------------------------CCATCCCATTACACCCGGGCCCCGGCGAGCAA 1140

GCTAGATGAAAATCGTGCACCTGTCGTCCATGGAGTGCATCGTCTGCATAGCTGTTTCGTAATTGCACCCATCCCATTACACCCGGGCCCCGGCGAGCAA 1140

AACAGTACCCGGACCTAGCCTGCAACCCCCAAGCCCCCGACGCGGTACATGCACGCATACACACACACGAAAGAGAGCGGTGCACGCAGTACGTACACAC 1240

AACAGTACCCGGACCTAGCCTGCAACCCCCAAGCCCCCGACGCGGTACATGCACGCATACACACACACGAAAGAGAGCGGTGCACGCAGTACGTACACAC 1240

CGGCAGGCGGACGCGGTACGTGCTAGGCTAGGCCAGGCTAGATTGGTCCAGCTGCCGGCTCCCCCCGTGCTCTCGTCGGCACGGCGCGACATGCCGTCAC 1340

CGGCAGGCGGACGCGGTACGTGCTAGGCTAGGCCAGGCTAGATTGGTCCAGCTGCCGGCTCCCCCCGTGCTCTCGTCGGCACGGCGCGACATGCCGTCAC 1340

GTACCTGCTCCGCCTGTGGCCCTTGGCGCTTGCGCCCGGCCGGCCGCGGTCGGGTCAACACACGAGGCCTCCCAGATCGGGCGCGGCATGCATGTGCCGC 1440

GTACCTGCTCCGCCTGTGGCCCTTGGCGCTTGCGCCCGGCCGGCCGCGGTCGGGTCAACACACGAGGCCTCCCAGATCGGGCGCGGCATGCATGTGCCGC 1440

CGGTACGTATGTACGTATACCGGCGCGGATTAATTTAGAGTTCGATTTGATTAGAGGGAGGGAGGGGGGCGTGCGGTGCACTTGGGCAATGTAATGCGGT 1540

CGGTACGTATGTACGTATACCGGCGCGGATTAATTTAGAGTTCGATTTGATTAGAGGGAGGGAGGGGGGCGTGCGGTGCACTTGGGCAATGTAATGCGGT 1540

CCTGCGAGGAGGGATCTCATCTAACCTAGCAGCACAGGGCGTACGGCCGGGGGCTTATCTTACTCTCGCTAGGTGCCTAAGATAACCAGCATGAGTTGAT 1640

CCTGCGAGGAGGGATCTCATCTAACCTAGCAGCACAGGGCGTACGGCCGGGGGCTTATCTTACTCTCGCTAGGTGCCTAAGATAACCAGCATGAGTTGAT 1640

GGTGCCGGCCGTTAACAATTCCAACCAAAAGCTAATCGTCTGGTGCACACGCAATGGTGGACACTATCATACCATGGATCACGTGGGTGGTTCTTTGTCC 1740

GGTGCCGGCCGTTAACAATTCCAACCAAAAGCTAATCGTCTGGTGCACACGCAATGGTGGACACTATCATACCATGGATCACGTGGGTGGTTCTTTGTTC 1740

ATGCCACTGGTTTTTCCTCTTTCGCTAGAGTTAATTAAGCGAATGGCTTTTGAATCCGTGTTTGTTCATGTCGGTCAAATCAAATCTCATGAATATGTTG 1840

ATGCCACTGGTTTTTCCTCTTTCGCTAGAGTTAATTAAGCGAATGGCTTTTGAATCCGTGTTTGTTCATGTCGGTCAAATCAAATCTCATGAATATGTTG 1840

TACCGTTTGTCCTAGATGGAGCTGGATTAAATAATTTCGGCATTTGTGGGTGCCCCTCCATACGATGTACTGGAGAGGGTTTTAAAACATTTTGTTGCTT 1940

TACCGTTTGTCCTAGATGGAGCTGGATTAAATAATTTCGGCATTTGTGGGTGCCCCTCCATACGATGTACTGGAGAGGGTTTTAAAACATTTTGTTGCTT 1940

AGCGTTTGGCTAGCGATGTAATAACAAAATAGTGTCATGACATCTGCCACAAAGGTGCGTCGACATCCATACTTTACTCTTCGTTGCACTACAAACATAT 2040

AGCGTTTGGCTAGCGATGTAATAACAAAATAGTGTCATGACATCTGCCACAAAGGTGCGTCGACATCCATACTTTACTCTTCGTTGCACTACAAACATAT 2040

TTTGTGATGTACGCTCCGTGTGATATATATACTTGTATCTTTAGAAAGGCCTAGAGTCTTAGGTGTAAGACCTTTCTAGCTGCAGGCCTGATTCGCACAA 2140

TTTGTGATGTACGCTCCGTGTGATATATATACTTGTATCTTTAGAAAGGCCTAGAGTCTTAGGTGTAAGACCTTTCTAGCTGCAGGCCTGATTCGCACAA 2140

GGTGCTACTTGGTTGTTGCATATAAAAAACGGTAATACTATAATACTCTGTCCCTCCGGGACTAAATTACTTAGCAAGTTGACATTTGTTTATATTTAGC 2240

GGTGCTACTTGGTTGTTGCATATAAAAAACGGTAATACTATAATACTCTGTCCCTCCGGGACTAAATTACTTAGCAAGTTGACATTTGTTTATATTTAGC 2240

TTCGGGAACGTCCAACGTCTCCGGTGTAAGACCTTTCTAGTCGCATTCCTGATTCAAACAAGATGCTACTTGGTTGTATATAAAATGATTGGGCTATAAT 2340

TTCGGGAACGTCCAACGTCTCCGGTGTAAGACCTTTCTAGTCGCATTCCTGATTCAAACAAGATGCTACTTGGTTGTATATAAAATGATTGGGCTATAAT 2340

ACTCCTCTTCGCAACAATTAAATTACTCAGTAGGTGAACATTTTATATATTCAACTCTAGCATATAATACAAATTTGTTGTCGGCATAGAAATGAGTTGC 2440

ACTCCTCTTCGCAACAATTAAATTACTCAGTAGGTGAACATTTTATATATTCAACTCTAGCATATAATACAAATTTGTTGTCGGCATAGAAATGAGTTGC 2440

AATCATTTCATAAATAGGAAGAACACATGGCTTATACCAAACTAGCAGCCTAAAAAGGTTTTTTTTTCTTCTTTCTGAGAGGAGGCATTTAGCTTGTGGA 2540

AATCATTTCATAAATAGGAAGAACACATGGCTTATACCAAACTAGCAGCCTAAAAAGGTTTTTTTTTCTTCTTTCTGAGAGGAGGCATTTAGCTTGTGGA 2540

GCAAAATGTTGAAGCGGCTGGGCGAAAAAACCCTCGGCTGATCCGCGTGAGGGCACGACACGTGGCGTCCCGGTCCACGGGGTGTGTGGCCGTAGCGATT 2640

GCAAAATGTTGAAGCGGCTGGGCGAAAAAACCCTCGGCTGATCCGCGTGAGGGCACGACACGTGGCGTCCCGGTCCACGGGGTGTGTGGCCGTAGCGATT 2640

AGCGAGGCTCCGCTGCACAAAAATAGTTTACCTCTGATGCCCTTGGCCTCCCCGACGTCCCATCTCGCTTTCTCTCTTTCTCTTTCTCCCACTGGCCTGG 2740

AGCGAGGCTCCGCTGCACAAAAATAGTTTACCTCTGATGCCCTTGGCCTCCCCGACGTCCCATCTCGCTTTCTCTCTTTCTCTTTCTCCCACTGGCCTGG 2740

CCCCCTCTCCTCGTCGTCCTCCAGTCCTCATCCCCCGCCCCATGGCCACCCCCACCACGGCCGCCCCCCCCCCCCCTCCCCCCACCACTACTTCTACTCC 2840

CCCCCTCTCCTCGTCGTCCTCCAGTCCTCATCCCCCGCCCCATGGCCACCCCCACCACGGCCGCCCCCCCCCCCCCTCCCCCCACCACTACTTCTACTCC 2840

CCCCGCCCCGCCCTCGCAGCCCGCGGCCACCGCGCTCCCATGCCATAGACGCGACCCCACTCATCGGTCCAGGTC 2915

CCCCGCCCCGCCCTCGCAGCCCGCGGCCACCGCGCTCCCATGCCATAGACGCGACCCCACTCATCGGTCCAGGTC 2915

**Figure S7.** Alignment of the *Q* gene (chromosome 5A) promoter from cultivars Chinese Spring and Bobwhite. The top sequence is Chinese Spring, and the bottom sequence is cultivar Bobwhite. The promoter region is 2,990 bp-long upstream of the start codon of the *Q* gene in both cultivars. The indel in the alignment are shown as “-”; the mismatch in the alignment is shown in red fonts. The position of the last nucleotide in each row is shown on the right side of the alignment.

**Figure S8.** Expression of the *Q* gene’s A genome homoeolog in the M1 plants at 6-leaf stage. The A genome specific primers, Q5ArtF4 and Q5ArtR4 (Table S1), were applied. *TaActin* gene was used as reference. The 3^rd^ and 6^th^ leaf in M1 plants at 6-leaf stage were sampled for RNA isolation followed by reverse transcription to get cDNA. The plant pQT-C413-1-12-28-7 and pQT-C413-1-12-28-21 carrying homozygous long deletions are highlighted with red box in the bar plots. All the rest of the plants have wild type alleles all the promoter of Q gene. The results are shown as mean ± SE based on three technical repeats.

**Figure S9.** The *Q*-5A gene expression level in spikes of M2 plants with edits in the *Q*-5A promoter. The morphology of mature spikes for wild-type and mutants is shown at the bottom. The spikes of M2 progeny of pQT-C413-1-12-28-6 (wild type), pQT-C413-1-12-28-7 (71 bp deletion) and pQT-C413-1-12-28-21 (387 bp deletion) were grown, and the spikes were sampled at Zadok stage 50. The expression levels were assessed using qRT-PCR.

**Figure S10.** Validation of the A genome-specific primers for RT-PCR of the *Q* gene. PCR amplification of cv. Bobwhite, cv. Chinese Spring (CS) and nullisomic-tetrasomic lines using the genome-specific primer Q5ArtF4 and Q5ArtR4 (Table S1) was performed. DNA isolated from six nullisomic-tetrasomic lines (N5A-T5D, N5B-T5A, N5DT5A, and N5DT5B), cv. Chinese Spring (CS), and cv. Bobwhite (BW). NTC is no template control of PCRs.

**Figure S11.** The somatic editing efficiency of BSMV-QT1 and BSMV-GW7T6 in line 3613474 and the somatic editing efficiency of BSMV-GW7T6 in KS080093K-18. The editing efficiency was obtained in the 4^th^ leaf developed after the inoculation. The data from all three genomes are separated to calculate the somatic editing efficiency for both targets.

**Figure S12.** The Cas9 plasmids used for creating transgenic plants. The plasmid pA9-Cas9 was used to make transgenic line 7438. The plasmid pBUN421 was used to make transgenic lines 707 and C413. The plasmid backbone was shown with black lines. The promoters are shown as white color filled arrows. The terminators are shown as grey color filled rectangles. The nuclear localization signal peptides and 3 tandem FLAG tags coding sequences are shown in yellow or dark blue rectangles. The wheat or maize codon optimized Cas9 coding sequences are shown in light blue rectangles.

**Table S1.** The primers, oligos and synthesized double-strand DNA used in this study.

| **Names** | **Sequences** | **Notes** | **Usage** |
| --- | --- | --- | --- |
| QT1-F | cttgatgaggaactggaccaagg |  | gRNA QT1 |
| QT1-R | aaacccttggtccagttcctcat |  | gRNA QT1 |
| QT1check-F | tgagcgactacgaggaggat |  | gRNA QT1 checking |
| QT1check-R | cagctgccctgtcacatcta |  | gRNA QT1 checking |
|  |  |  |  |
| targetVIGSsF | ATATTAATTAACGCTTGCTGCATCAGACTTG | Pac1 cut site is in red | Amplify sgRNA from CRISPR-Cas9 plasmids and insert into BSMV gamma chain plasmid on the sense direction. |
| targetVIGSsR | TATGCGGCCGCAAAGCACCGACTCGGTGCC | Not1 cut site is in red |  |
| targetVIGSaF | ATATTAATTAAAAAGCACCGACTCGGTGCC | Pac1 cut site is in red | Amplify sgRNA from CRISPR-Cas9 plasmids and insert into BSMV gamma chain plasmid on the antisense direction. |
| targetVIGSaR | TATGCGGCCGCCGCTTGCTGCATCAGACTTG | Not1 cut site is in red |  |
| VIGS-GW2T2sgRNA-F | CTTCCGTTGCTAGCTGATTAATTAAgctcgcgccctgctacccggGTTTTAGAGCTAGAA | Capital letters on the 5' end overlap with PacI and NotI digested pBSMVgammaPDS, lower case letters in the middle are the spacer of GW2T2, capital letters on the 3' end is specific primers fo 5' end of sgRNA scafold. | Amplify sgRNA from CRISPR-Cas9 plasmids and insert into BSMV gamma chain plasmid using Gibson reaction. |
| VIGS-sgRNA-R | tttttttagctagctgagcggccgcGCACCGACTCGGTGCCACTT | Lower case letters on the 5' end overlap with PacI and NotI digested pBSMVgammaPDS, capital letters on the 3' end is specific primers fo 3' end of sgRNA scafold. |  |
| SgRNA scalfold::AtFT_CDS | CTTCCGTTGCTAGCTGATTAATTAAgctcgcgccctgctacccggGTTTTAGAGCTAGAAATAGCAAGTTAAAATAAGGCTAGTCCGTTATCAACTTGAAAAAGTGGCACCGAGTCGGTGCatgtctataaatataagagaccctcttatagtaagcagagttgttggagacgttcttgatccgtttaatagatcaatcactctaaaggttacttatggccaaagagaggtgactaatggcttggatctaaggccttctcaggttcaaaacaagccaagagttgagattggtggagaagacctcaggaacttctatactttggttatggtggatccagatgttccaagtcctagcaaccctcacctccgagaatatctccattggttggtgactgatatccctgctacaactggaacaacctttggcaatgagattgtgtgttacgaaaatccaagtcccactgcaggaattcatcgtgtcgtgtttatattgtttcgacagcttggcaggcaaacagtgtatgcaccagggtggcgccagaacttcaacactcgcgagtttgctgagatctacaatctcggccttcccgtggccgcagttttctacaattgtcagagggagagtggctgcggaggaagaagactttagGCGGCCGCTCAGCTAGCTAAAAAAA | Each part are differenttiated with upper and lower case letters. From left to right, overlap with plasmid, spacer, sgRNA scalfold, mobile elements, overlap with plasmid. | Construct BSMV gamma chain plasmid with fused sgRNA and mobile elements. |
| SgRNA scalfold::Vrn3 CDS | CTTCCGTTGCTAGCTGATTAATTAAgctcgcgccctgctacccggGTTTTAGAGCTAGAAATAGCAAGTTAAAATAAGGCTAGTCCGTTATCAACTTGAAAAAGTGGCACCGAGTCGGTGCatggccggtagggatagggacccgctggtggttggcagggttgtgggggacgtgctggaccccttcgtccggaccaccaacctcagggtgaccttcgggaacaggaccgtgtccaacggctgcgagctcaagccgtccatggtcgcccagcagcccagggttgaggtgggcggcaatgagatgaggaccttctacacactcgtgatggtagacccagatgctccaagtccaagcgatcccaaccttagggagtatctccactggcttgtgacagatatccccggtacaactggtgcgtcgttcgggcaggaggtgatgtgctacgagagccctcgtccgaccatggggatccaccgcttcgtgctcgtactcttccagcagctcgggcggcagacggtgtacgcccccgggtggcgccagaacttcaacaccagggacttcgccgagctctacaacctcggcccgcctgtcgccgccgtctacttcaactgccagcgtgaggccggctccggcggcaggaggatgtacaattgaGCGGCCGCTCAGCTAGCTAAAAAAA |  |  |
| SgRNA scalfold::tRNA^Met^ | CTTCCGTTGCTAGCTGATTAATTAAgctcgcgccctgctacccggGTTTTAGAGCTAGAAATAGCAAGTTAAAATAAGGCTAGTCCGTTATCAACTTGAAAAAGTGGCACCGAGTCGGTGCatcagagtggcgcagcggaagcgtggtgggcccataacccacaggtcccaggatcgaaacctggctctgataGCGGCCGCTCAGCTAGCTAAAAAAA |  |  |
| SgRNA scalfold::tRNA^Ile^ | CTTCCGTTGCTAGCTGATTAATTAAgctcgcgccctgctacccggGTTTTAGAGCTAGAAATAGCAAGTTAAAATAAGGCTAGTCCGTTATCAACTTGAAAAAGTGGCACCGAGTCGGTGCgctcccgtagctcagttggttagagcgttggtcttatgagccgaaggtcgcgggttcgagccccgccgggagcaGCGGCCGCTCAGCTAGCTAAAAAAA |  |  |
|  |  |  |  |
| BSMV-UPL3T11-SgRNA | CTTCCGTTGCTAGCTGATTAATTAAcaaggagcagcaggagccctGTTTTAGAGCTAGAAATAGCAAGTTAAAATAAGGCTAGTCCGTTATCAACTTGAAAAAGTGGCACCGAGTCGGTGCgcggccgctcagctagctaaaaaaa | Each part are differenttiated with upper and lower case letters. From left to right, overlap with plasmid, spacer, sgRNA scalfold, overlap with plasmid. | Construct BSMV gamma chain plasmid by inserting into BSMV gamma chain plasmid using Gibson reaction. |
| VIGS-pQT17FsgRNA-F | CTTCCGTTGCTAGCTGATTAATTAAgtttccgatggggcgagcggGTTTTAGAGCTAGAA | Capital letters on the 5' end overlap with PacI and NotI digested pBSMVgammaPDS, lower case letters in the middle are the spacer of target sites, capital letters on the 3' end is specific primers fo 5' end of sgRNA scafold. | Work in pair with primer VIGS-sgRNA-R, amplify sgRNA from CRISPR-Cas9 plasmids and insert into BSMV gamma chain plasmid using Gibson reaction. |
| VIGS-pQT18FsgRNA-F | CTTCCGTTGCTAGCTGATTAATTAAgacgtagacccacgcgagggGTTTTAGAGCTAGAA |  |  |
| VIGS-pQT23FsgRNA-F | CTTCCGTTGCTAGCTGATTAATTAAcgtgccgacgagagcacgggGTTTTAGAGCTAGAA |  |  |
| VIGS-pQT25FsgRNA-F | CTTCCGTTGCTAGCTGATTAATTAAagcacagggcgtacggccggGTTTTAGAGCTAGAA |  |  |
| VIGS-pQT26FsgRNA-F | CTTCCGTTGCTAGCTGATTAATTAAcgctacggccacacaccccgGTTTTAGAGCTAGAA |  |  |
| VIGS-GW7T6FsgRNA-F | CTTCCGTTGCTAGCTGATTAATTAAtccatcaaccgggactcgggGTTTTAGAGCTAGAA |  |  |
|  |  |  |  |
| BSMVseqL | GGAGCTGAAACTTTCTCATATCG |  | Sanger sequencing of pBSMVgamma constructs with insertions |
| BSMVseqR | GTGGACTTGCAAACACTCCC |  |  |
|  |  |  |  |
| Q5ArtF4 | CTGATGCTCTTGACTTGGATCTG |  | Check the expression of Q gene A genome allele |
| Q5ArtR4 | TTTTGTTTCTTTACCTGAGAAGAGAT |  |  |
| zCAS9-F | CGGACTAGTATGGATTACAAGGACCACGACG |  | Check the expression of Cas9 in transgenic plants |
| zCAS9seq1 | accttgtactcgtcggtgatcac |  |  |
| TaActin-F | ACCTTCAGTTGCCCAGCAAT |  | Amplify TaActin gene as reference to check the expression level of other genes. |
| TaActin-R | CAGAGTCGAGCACAATACCAGTTG |  |  |
|  |  |  |  |
| QT1MiseqF1 | ctctttccctacacgacgctcttccgatctCGCTTtggtttgtccgatggttgat | The target specific primers are shown as lower case letter on the 3' end, the 5 additional barcoding nucleotides are shown as upper case letters, the 5' end lower case letters are part of Illumina Truseq adapter. | The first round PCR primers for NGS library of target site QT1. |
| QT1MiseqF2 | ctctttccctacacgacgctcttccgatctCTAGCtggtttgtccgatggttgat |  |  |
| QT1MiseqF3 | ctctttccctacacgacgctcttccgatctACAAAtggtttgtccgatggttgat |  |  |
| QT1MiseqF4 | ctctttccctacacgacgctcttccgatctTTCTCtggtttgtccgatggttgat |  |  |
| QT1MiseqF5 | ctctttccctacacgacgctcttccgatctAGCCCtggtttgtccgatggttgat |  |  |
| QT1MiseqF6 | ctctttccctacacgacgctcttccgatctGTATTtggtttgtccgatggttgat |  |  |
| QT1MiseqF7 | ctctttccctacacgacgctcttccgatctCTGTAtggtttgtccgatggttgat |  |  |
| QT1MiseqF8 | ctctttccctacacgacgctcttccgatctACCGTtggtttgtccgatggttgat |  |  |
| QT1MiseqR1 | ctggagttcagacgtgtgctcttccgatctGCTTAtgtgcctgcttacttcttgc |  |  |
| QT1MiseqR2 | ctggagttcagacgtgtgctcttccgatctGGTGTtgtgcctgcttacttcttgc |  |  |
| QT1MiseqR3 | ctggagttcagacgtgtgctcttccgatctAGGATtgtgcctgcttacttcttgc |  |  |
| QT1MiseqR4 | ctggagttcagacgtgtgctcttccgatctATTGAtgtgcctgcttacttcttgc |  |  |
| QT1MiseqR5 | ctggagttcagacgtgtgctcttccgatctCATCTtgtgcctgcttacttcttgc |  |  |
| QT1MiseqR6 | ctggagttcagacgtgtgctcttccgatctCCTACtgtgcctgcttacttcttgc |  |  |
| QT1MiseqR7 | ctggagttcagacgtgtgctcttccgatctGAGGAtgtgcctgcttacttcttgc |  |  |
| QT1MiseqR8 | ctggagttcagacgtgtgctcttccgatctGGAACtgtgcctgcttacttcttgc |  |  |
| QT1MiseqR9 | ctggagttcagacgtgtgctcttccgatctGTCAAtgtgcctgcttacttcttgc |  |  |
| QT1MiseqR10 | ctggagttcagacgtgtgctcttccgatctTAATAtgtgcctgcttacttcttgc |  |  |
| QT1MiseqR11 | ctggagttcagacgtgtgctcttccgatctTACATtgtgcctgcttacttcttgc |  |  |
| QT1MiseqR12 | ctggagttcagacgtgtgctcttccgatctTCGTTtgtgcctgcttacttcttgc |  |  |
|  |  |  |  |
| GW2T2MiseqF1 | ctctttccctacacgacgctcttccgatctCTCCatggggaacagaataggagg | The target specific primers are shown as lower case letter on the 3' end, the 5 additional barcoding nucleotides are shown as upper case letters, the 5' end lower case letters are part of Illumina Truseq adapter. | The first round PCR primers for NGS library of target site GW2T2. |
| GW2T2MiseqF2 | ctctttccctacacgacgctcttccgatctTGCAatggggaacagaataggagg |  |  |
| GW2T2MiseqF3 | ctctttccctacacgacgctcttccgatctACTAatggggaacagaataggagg |  |  |
| GW2T2MiseqF4 | ctctttccctacacgacgctcttccgatctCAGAatggggaacagaataggagg |  |  |
| GW2T2MiseqF5 | ctctttccctacacgacgctcttccgatctAACTatggggaacagaataggagg |  |  |
| GW2T2MiseqF6 | ctctttccctacacgacgctcttccgatctGCGTatggggaacagaataggagg |  |  |
| GW2T2MiseqF7 | ctctttccctacacgacgctcttccgatctCGATatggggaacagaataggagg |  |  |
| GW2T2MiseqF8 | ctctttccctacacgacgctcttccgatctGTAAatggggaacagaataggagg |  |  |
| GW2T2MiseqR1 | ctggagttcagacgtgtgctcttccgatctCTCCaggaagcagatggggcactc |  |  |
| GW2T2MiseqR2 | ctggagttcagacgtgtgctcttccgatctTGCAaggaagcagatggggcactc |  |  |
| GW2T2MiseqR3 | ctggagttcagacgtgtgctcttccgatctACTAaggaagcagatggggcactc |  |  |
| GW2T2MiseqR4 | ctggagttcagacgtgtgctcttccgatctCAGAaggaagcagatggggcactc |  |  |
| GW2T2MiseqR5 | ctggagttcagacgtgtgctcttccgatctAACTaggaagcagatggggcactc |  |  |
| GW2T2MiseqR6 | ctggagttcagacgtgtgctcttccgatctGCGTaggaagcagatggggcactc |  |  |
| GW2T2MiseqR7 | ctggagttcagacgtgtgctcttccgatctCGATaggaagcagatggggcactc |  |  |
| GW2T2MiseqR8 | ctggagttcagacgtgtgctcttccgatctGTAAaggaagcagatggggcactc |  |  |
| GW2T2MiseqR9 | ctggagttcagacgtgtgctcttccgatctAGGCaggaagcagatggggcactc |  |  |
| GW2T2MiseqR10 | ctggagttcagacgtgtgctcttccgatctGATCaggaagcagatggggcactc |  |  |
| GW2T2MiseqR11 | ctggagttcagacgtgtgctcttccgatctTCACaggaagcagatggggcactc |  |  |
| GW2T2MiseqR12 | ctggagttcagacgtgtgctcttccgatctTGCGAaggaagcagatggggcactc |  |  |
|  |  |  |  |
| GW7T6MiseqF1 | ctctttccctacacgacgctcttccgatctCGCTTgacctttcggtttattttgcag | The target specific primers are shown as lower case letter on the 3' end, the 5 additional barcoding nucleotides are shown as upper case letters, the 5' end lower case letters are part of Illumina Truseq adapter. | The first round PCR primers for NGS library of target site GW7T6. |
| GW7T6MiseqF2 | ctctttccctacacgacgctcttccgatctCTAGCgacctttcggtttattttgcag |  |  |
| GW7T6MiseqF3 | ctctttccctacacgacgctcttccgatctACAAAgacctttcggtttattttgcag |  |  |
| GW7T6MiseqF4 | ctctttccctacacgacgctcttccgatctTTCTCgacctttcggtttattttgcag |  |  |
| GW7T6MiseqF5 | ctctttccctacacgacgctcttccgatctAGCCCgacctttcggtttattttgcag |  |  |
| GW7T6MiseqF6 | ctctttccctacacgacgctcttccgatctGTATTgacctttcggtttattttgcag |  |  |
| GW7T6MiseqF7 | ctctttccctacacgacgctcttccgatctCTGTAgacctttcggtttattttgcag |  |  |
| GW7T6MiseqF8 | ctctttccctacacgacgctcttccgatctACCGTgacctttcggtttattttgcag |  |  |
| GW7T6MiseqR1 | ctggagttcagacgtgtgctcttccgatctGCTTAgtaggttccgtccatcgattt |  |  |
| GW7T6MiseqR2 | ctggagttcagacgtgtgctcttccgatctGGTGTgtaggttccgtccatcgattt |  |  |
| GW7T6MiseqR3 | ctggagttcagacgtgtgctcttccgatctAGGATgtaggttccgtccatcgattt |  |  |
| GW7T6MiseqR4 | ctggagttcagacgtgtgctcttccgatctATTGAgtaggttccgtccatcgattt |  |  |
| GW7T6MiseqR5 | ctggagttcagacgtgtgctcttccgatctCATCTgtaggttccgtccatcgattt |  |  |
| GW7T6MiseqR6 | ctggagttcagacgtgtgctcttccgatctCCTACgtaggttccgtccatcgattt |  |  |
| GW7T6MiseqR7 | ctggagttcagacgtgtgctcttccgatctGAGGAgtaggttccgtccatcgattt |  |  |
| GW7T6MiseqR8 | ctggagttcagacgtgtgctcttccgatctGGAACgtaggttccgtccatcgattt |  |  |
| GW7T6MiseqR9 | ctggagttcagacgtgtgctcttccgatctGTCAAgtaggttccgtccatcgattt |  |  |
| GW7T6MiseqR10 | ctggagttcagacgtgtgctcttccgatctTAATAgtaggttccgtccatcgattt |  |  |
| GW7T6MiseqR11 | ctggagttcagacgtgtgctcttccgatctTACATgtaggttccgtccatcgattt |  |  |
| GW7T6MiseqR12 | ctggagttcagacgtgtgctcttccgatctTCGTTgtaggttccgtccatcgattt |  |  |
|  |  |  |  |
| UPL3T11MiF1 | ctctttccctacacgacgctcttccgatctCGCTTcccaaccctaaccctagcc | The target specific primers are shown as lower case letter on the 3' end, the 5 additional barcoding nucleotides are shown as upper case letters, the 5' end lower case letters are part of Illumina Truseq adapter. | The first round PCR primers for NGS library of target site UPL3T11. |
| UPL3T11MiF2 | ctctttccctacacgacgctcttccgatctCTAGCcccaaccctaaccctagcc |  |  |
| UPL3T11MiF3 | ctctttccctacacgacgctcttccgatctACAAAcccaaccctaaccctagcc |  |  |
| UPL3T11MiF4 | ctctttccctacacgacgctcttccgatctTTCTCcccaaccctaaccctagcc |  |  |
| UPL3T11MiF5 | ctctttccctacacgacgctcttccgatctAGCCCcccaaccctaaccctagcc |  |  |
| UPL3T11MiF6 | ctctttccctacacgacgctcttccgatctGTATTcccaaccctaaccctagcc |  |  |
| UPL3T11MiF7 | ctctttccctacacgacgctcttccgatctCTGTAcccaaccctaaccctagcc |  |  |
| UPL3T11MiF8 | ctctttccctacacgacgctcttccgatctACCGTcccaaccctaaccctagcc |  |  |
| UPL3T11MiR1 | ctggagttcagacgtgtgctcttccgatctGCTTActcgtcgtcgtcgtccat |  |  |
| UPL3T11MiR2 | ctggagttcagacgtgtgctcttccgatctGGTGTctcgtcgtcgtcgtccat |  |  |
| UPL3T11MiR3 | ctggagttcagacgtgtgctcttccgatctAGGATctcgtcgtcgtcgtccat |  |  |
| UPL3T11MiR4 | ctggagttcagacgtgtgctcttccgatctATTGActcgtcgtcgtcgtccat |  |  |
| UPL3T11MiR5 | ctggagttcagacgtgtgctcttccgatctCATCTctcgtcgtcgtcgtccat |  |  |
| UPL3T11MiR6 | ctggagttcagacgtgtgctcttccgatctCCTACctcgtcgtcgtcgtccat |  |  |
| UPL3T11MiR7 | ctggagttcagacgtgtgctcttccgatctGAGGActcgtcgtcgtcgtccat |  |  |
| UPL3T11MiR8 | ctggagttcagacgtgtgctcttccgatctGGAACctcgtcgtcgtcgtccat |  |  |
| UPL3T11MiR9 | ctggagttcagacgtgtgctcttccgatctGTCAActcgtcgtcgtcgtccat |  |  |
| UPL3T11MiR10 | ctggagttcagacgtgtgctcttccgatctTAATActcgtcgtcgtcgtccat |  |  |
| UPL3T11MiR11 | ctggagttcagacgtgtgctcttccgatctTACATctcgtcgtcgtcgtccat |  |  |
| UPL3T11MiR12 | ctggagttcagacgtgtgctcttccgatctTCGTTctcgtcgtcgtcgtccat |  |  |
|  |  |  |  |
| pQT17MiF1 | ctctttccctacacgacgctcttccgatctCGCTTgtgaccacgagcgtaaggtt | The target specific primers are shown as lower case letter on the 3' end, the 5 additional barcoding nucleotides are shown as upper case letters, the 5' end lower case letters are part of Illumina Truseq adapter. | The first round PCR primers for NGS library of target site pQT17. |
| pQT17MiF2 | ctctttccctacacgacgctcttccgatctCTAGCgtgaccacgagcgtaaggtt |  |  |
| pQT17MiF3 | ctctttccctacacgacgctcttccgatctACAAAgtgaccacgagcgtaaggtt |  |  |
| pQT17MiF4 | ctctttccctacacgacgctcttccgatctTTCTCgtgaccacgagcgtaaggtt |  |  |
| pQT17MiF5 | ctctttccctacacgacgctcttccgatctAGCCCgtgaccacgagcgtaaggtt |  |  |
| pQT17MiF6 | ctctttccctacacgacgctcttccgatctGTATTgtgaccacgagcgtaaggtt |  |  |
| pQT17MiF7 | ctctttccctacacgacgctcttccgatctCTGTAgtgaccacgagcgtaaggtt |  |  |
| pQT17MiF8 | ctctttccctacacgacgctcttccgatctACCGTgtgaccacgagcgtaaggtt |  |  |
| pQT17MiR1 | ctggagttcagacgtgtgctcttccgatctGCTTAccacaggcgtacctgtcc |  |  |
| pQT17MiR2 | ctggagttcagacgtgtgctcttccgatctGGTGTccacaggcgtacctgtcc |  |  |
| pQT17MiR3 | ctggagttcagacgtgtgctcttccgatctAGGATccacaggcgtacctgtcc |  |  |
| pQT17MiR4 | ctggagttcagacgtgtgctcttccgatctATTGAccacaggcgtacctgtcc |  |  |
| pQT17MiR5 | ctggagttcagacgtgtgctcttccgatctCATCTccacaggcgtacctgtcc |  |  |
| pQT17MiR6 | ctggagttcagacgtgtgctcttccgatctCCTACccacaggcgtacctgtcc |  |  |
| pQT17MiR7 | ctggagttcagacgtgtgctcttccgatctGAGGAccacaggcgtacctgtcc |  |  |
| pQT17MiR8 | ctggagttcagacgtgtgctcttccgatctGGAACccacaggcgtacctgtcc |  |  |
| pQT17MiR9 | ctggagttcagacgtgtgctcttccgatctGTCAAccacaggcgtacctgtcc |  |  |
| pQT17MiR10 | ctggagttcagacgtgtgctcttccgatctTAATAccacaggcgtacctgtcc |  |  |
| pQT17MiR11 | ctggagttcagacgtgtgctcttccgatctTACATccacaggcgtacctgtcc |  |  |
| pQT17MiR12 | ctggagttcagacgtgtgctcttccgatctTCGTTccacaggcgtacctgtcc |  |  |
|  |  |  |  |
| pQT18MiF1 | ctctttccctacacgacgctcttccgatctCGCTTaaggaacaacgtttccgatg | The target specific primers are shown as lower case letter on the 3' end, the 5 additional barcoding nucleotides are shown as upper case letters, the 5' end lower case letters are part of Illumina Truseq adapter. | The first round PCR primers for NGS library of target site pQT18. |
| pQT18MiF2 | ctctttccctacacgacgctcttccgatctCTAGCaaggaacaacgtttccgatg |  |  |
| pQT18MiF3 | ctctttccctacacgacgctcttccgatctACAAAaaggaacaacgtttccgatg |  |  |
| pQT18MiF4 | ctctttccctacacgacgctcttccgatctTTCTCaaggaacaacgtttccgatg |  |  |
| pQT18MiF5 | ctctttccctacacgacgctcttccgatctAGCCCaaggaacaacgtttccgatg |  |  |
| pQT18MiF6 | ctctttccctacacgacgctcttccgatctGTATTaaggaacaacgtttccgatg |  |  |
| pQT18MiF7 | ctctttccctacacgacgctcttccgatctCTGTAaaggaacaacgtttccgatg |  |  |
| pQT18MiF8 | ctctttccctacacgacgctcttccgatctACCGTaaggaacaacgtttccgatg |  |  |
| pQT18MiR1 | ctggagttcagacgtgtgctcttccgatctGCTTAcgctctctttcgtgtgtgtg |  |  |
| pQT18MiR2 | ctggagttcagacgtgtgctcttccgatctGGTGTcgctctctttcgtgtgtgtg |  |  |
| pQT18MiR3 | ctggagttcagacgtgtgctcttccgatctAGGATcgctctctttcgtgtgtgtg |  |  |
| pQT18MiR4 | ctggagttcagacgtgtgctcttccgatctATTGAcgctctctttcgtgtgtgtg |  |  |
| pQT18MiR5 | ctggagttcagacgtgtgctcttccgatctCATCTcgctctctttcgtgtgtgtg |  |  |
| pQT18MiR6 | ctggagttcagacgtgtgctcttccgatctCCTACcgctctctttcgtgtgtgtg |  |  |
| pQT18MiR7 | ctggagttcagacgtgtgctcttccgatctGAGGAcgctctctttcgtgtgtgtg |  |  |
| pQT18MiR8 | ctggagttcagacgtgtgctcttccgatctGGAACcgctctctttcgtgtgtgtg |  |  |
| pQT18MiR9 | ctggagttcagacgtgtgctcttccgatctGTCAAcgctctctttcgtgtgtgtg |  |  |
| pQT18MiR10 | ctggagttcagacgtgtgctcttccgatctTAATAcgctctctttcgtgtgtgtg |  |  |
| pQT18MiR11 | ctggagttcagacgtgtgctcttccgatctTACATcgctctctttcgtgtgtgtg |  |  |
| pQT18MiR12 | ctggagttcagacgtgtgctcttccgatctTCGTTcgctctctttcgtgtgtgtg |  |  |
|  |  |  |  |
| pQT23MiF1 | ctctttccctacacgacgctcttccgatctCGCTTaattgcacccatcccattac | The target specific primers are shown as lower case letter on the 3' end, the 5 additional barcoding nucleotides are shown as upper case letters, the 5' end lower case letters are part of Illumina Truseq adapter. | The first round PCR primers for NGS library of target site pQT23. |
| pQT23MiF2 | ctctttccctacacgacgctcttccgatctCTAGCaattgcacccatcccattac |  |  |
| pQT23MiF3 | ctctttccctacacgacgctcttccgatctACAAAaattgcacccatcccattac |  |  |
| pQT23MiF4 | ctctttccctacacgacgctcttccgatctTTCTCaattgcacccatcccattac |  |  |
| pQT23MiF5 | ctctttccctacacgacgctcttccgatctAGCCCaattgcacccatcccattac |  |  |
| pQT23MiF6 | ctctttccctacacgacgctcttccgatctGTATTaattgcacccatcccattac |  |  |
| pQT23MiF7 | ctctttccctacacgacgctcttccgatctCTGTAaattgcacccatcccattac |  |  |
| pQT23MiF8 | ctctttccctacacgacgctcttccgatctACCGTaattgcacccatcccattac |  |  |
| pQT23MiR1 | ctggagttcagacgtgtgctcttccgatctGCTTAaggcctcgtgtgttgacc |  |  |
| pQT23MiR2 | ctggagttcagacgtgtgctcttccgatctGGTGTaggcctcgtgtgttgacc |  |  |
| pQT23MiR3 | ctggagttcagacgtgtgctcttccgatctAGGATaggcctcgtgtgttgacc |  |  |
| pQT23MiR4 | ctggagttcagacgtgtgctcttccgatctATTGAaggcctcgtgtgttgacc |  |  |
| pQT23MiR5 | ctggagttcagacgtgtgctcttccgatctCATCTaggcctcgtgtgttgacc |  |  |
| pQT23MiR6 | ctggagttcagacgtgtgctcttccgatctCCTACaggcctcgtgtgttgacc |  |  |
| pQT23MiR7 | ctggagttcagacgtgtgctcttccgatctGAGGAaggcctcgtgtgttgacc |  |  |
| pQT23MiR8 | ctggagttcagacgtgtgctcttccgatctGGAACaggcctcgtgtgttgacc |  |  |
| pQT23MiR9 | ctggagttcagacgtgtgctcttccgatctGTCAAaggcctcgtgtgttgacc |  |  |
| pQT23MiR10 | ctggagttcagacgtgtgctcttccgatctTAATAaggcctcgtgtgttgacc |  |  |
| pQT23MiR11 | ctggagttcagacgtgtgctcttccgatctTACATaggcctcgtgtgttgacc |  |  |
| pQT23MiR12 | ctggagttcagacgtgtgctcttccgatctTCGTTaggcctcgtgtgttgacc |  |  |
|  |  |  |  |
| pQT25MiF1 | ctctttccctacacgacgctcttccgatctCGCTTtgcacttgggcaatgtaatg | The target specific primers are shown as lower case letter on the 3' end, the 5 additional barcoding nucleotides are shown as upper case letters, the 5' end lower case letters are part of Illumina Truseq adapter. | The first round PCR primers for NGS library of target site pQT25. |
| pQT25MiF2 | ctctttccctacacgacgctcttccgatctCTAGCtgcacttgggcaatgtaatg |  |  |
| pQT25MiF3 | ctctttccctacacgacgctcttccgatctACAAAtgcacttgggcaatgtaatg |  |  |
| pQT25MiF4 | ctctttccctacacgacgctcttccgatctTTCTCtgcacttgggcaatgtaatg |  |  |
| pQT25MiF5 | ctctttccctacacgacgctcttccgatctAGCCCtgcacttgggcaatgtaatg |  |  |
| pQT25MiF6 | ctctttccctacacgacgctcttccgatctGTATTtgcacttgggcaatgtaatg |  |  |
| pQT25MiF7 | ctctttccctacacgacgctcttccgatctCTGTAtgcacttgggcaatgtaatg |  |  |
| pQT25MiF8 | ctctttccctacacgacgctcttccgatctACCGTtgcacttgggcaatgtaatg |  |  |
| pQT25MiR1 | ctggagttcagacgtgtgctcttccgatctGCTTAagtggcatggacaaagaacc |  |  |
| pQT25MiR2 | ctggagttcagacgtgtgctcttccgatctGGTGTagtggcatggacaaagaacc |  |  |
| pQT25MiR3 | ctggagttcagacgtgtgctcttccgatctAGGATagtggcatggacaaagaacc |  |  |
| pQT25MiR4 | ctggagttcagacgtgtgctcttccgatctATTGAagtggcatggacaaagaacc |  |  |
| pQT25MiR5 | ctggagttcagacgtgtgctcttccgatctCATCTagtggcatggacaaagaacc |  |  |
| pQT25MiR6 | ctggagttcagacgtgtgctcttccgatctCCTACagtggcatggacaaagaacc |  |  |
| pQT25MiR7 | ctggagttcagacgtgtgctcttccgatctGAGGAagtggcatggacaaagaacc |  |  |
| pQT25MiR8 | ctggagttcagacgtgtgctcttccgatctGGAACagtggcatggacaaagaacc |  |  |
| pQT25MiR9 | ctggagttcagacgtgtgctcttccgatctGTCAAagtggcatggacaaagaacc |  |  |
| pQT25MiR10 | ctggagttcagacgtgtgctcttccgatctTAATAagtggcatggacaaagaacc |  |  |
| pQT25MiR11 | ctggagttcagacgtgtgctcttccgatctTACATagtggcatggacaaagaacc |  |  |
| pQT25MiR12 | ctggagttcagacgtgtgctcttccgatctTCGTTagtggcatggacaaagaacc |  |  |
|  |  |  |  |
| pQT26MiF1 | ctctttccctacacgacgctcttccgatctCGCTTaggaggcatttagcttgtggag | The target specific primers are shown as lower case letter on the 3' end, the 5 additional barcoding nucleotides are shown as upper case letters, the 5' end lower case letters are part of Illumina Truseq adapter. | The first round PCR primers for NGS library of target site pQT26. |
| pQT26MiF2 | ctctttccctacacgacgctcttccgatctCTAGCaggaggcatttagcttgtggag |  |  |
| pQT26MiF3 | ctctttccctacacgacgctcttccgatctACAAAaggaggcatttagcttgtggag |  |  |
| pQT26MiF4 | ctctttccctacacgacgctcttccgatctTTCTCaggaggcatttagcttgtggag |  |  |
| pQT26MiF5 | ctctttccctacacgacgctcttccgatctAGCCCaggaggcatttagcttgtggag |  |  |
| pQT26MiF6 | ctctttccctacacgacgctcttccgatctGTATTaggaggcatttagcttgtggag |  |  |
| pQT26MiF7 | ctctttccctacacgacgctcttccgatctCTGTAaggaggcatttagcttgtggag |  |  |
| pQT26MiF8 | ctctttccctacacgacgctcttccgatctACCGTaggaggcatttagcttgtggag |  |  |
| pQT26MiR1 | ctggagttcagacgtgtgctcttccgatctGCTTAgccagtgggagaaagagaaaga |  |  |
| pQT26MiR2 | ctggagttcagacgtgtgctcttccgatctGGTGTgccagtgggagaaagagaaaga |  |  |
| pQT26MiR3 | ctggagttcagacgtgtgctcttccgatctAGGATgccagtgggagaaagagaaaga |  |  |
| pQT26MiR4 | ctggagttcagacgtgtgctcttccgatctATTGAgccagtgggagaaagagaaaga |  |  |
| pQT26MiR5 | ctggagttcagacgtgtgctcttccgatctCATCTgccagtgggagaaagagaaaga |  |  |
| pQT26MiR6 | ctggagttcagacgtgtgctcttccgatctCCTACgccagtgggagaaagagaaaga |  |  |
| pQT26MiR7 | ctggagttcagacgtgtgctcttccgatctGAGGAgccagtgggagaaagagaaaga |  |  |
| pQT26MiR8 | ctggagttcagacgtgtgctcttccgatctGGAACgccagtgggagaaagagaaaga |  |  |
| pQT26MiR9 | ctggagttcagacgtgtgctcttccgatctGTCAAgccagtgggagaaagagaaaga |  |  |
| pQT26MiR10 | ctggagttcagacgtgtgctcttccgatctTAATAgccagtgggagaaagagaaaga |  |  |
| pQT26MiR11 | ctggagttcagacgtgtgctcttccgatctTACATgccagtgggagaaagagaaaga |  |  |
| pQT26MiR12 | ctggagttcagacgtgtgctcttccgatctTCGTTgccagtgggagaaagagaaaga |  |  |
|  |  |  |  |
| PCR_Truseq_Amp_F | AATGATACGGCGACCACCGAGATCTACACTCTTTCCCTACACGAC | The red color upper case letters are Illumina Truseq barcodes, the black color upper case letters are part of Illumina Truseq adapter. PCR_Truseq_Amp_F is the forward primer while all others are reverse primers. The numbers in the name of reverse primers are the number of Truseq barcodes. | The second round PCR for NGS libraries. |
| PCR_Truseq_Amp_R_1 | CAAGCAGAAGACGGCATACGAGATCGTGATGTGACTGGAGTTCAGACG |  |  |
| PCR_Truseq_Amp_R_2 | CAAGCAGAAGACGGCATACGAGATACATCGGTGACTGGAGTTCAGACG |  |  |
| PCR_Truseq_Amp_R_3 | CAAGCAGAAGACGGCATACGAGATGCCTAAGTGACTGGAGTTCAGACG |  |  |
| PCR_Truseq_Amp_R_4 | CAAGCAGAAGACGGCATACGAGATTGGTCAGTGACTGGAGTTCAGACG |  |  |
| PCR_Truseq_Amp_R_5 | CAAGCAGAAGACGGCATACGAGATCACTGTGTGACTGGAGTTCAGACG |  |  |
| PCR_Truseq_Amp_R_6 | CAAGCAGAAGACGGCATACGAGATATTGGCGTGACTGGAGTTCAGACG |  |  |
| PCR_Truseq_Amp_R_7 | CAAGCAGAAGACGGCATACGAGATGATCTGGTGACTGGAGTTCAGACG |  |  |
| PCR_Truseq_Amp_R_8 | CAAGCAGAAGACGGCATACGAGATTCAAGTGTGACTGGAGTTCAGACG |  |  |
| PCR_Truseq_Amp_R_9 | CAAGCAGAAGACGGCATACGAGATCTGATCGTGACTGGAGTTCAGACG |  |  |
| PCR_Truseq_Amp_R_10 | CAAGCAGAAGACGGCATACGAGATAAGCTAGTGACTGGAGTTCAGACG |  |  |
| PCR_Truseq_Amp_R_11 | CAAGCAGAAGACGGCATACGAGATGTAGCCGTGACTGGAGTTCAGACG |  |  |
| PCR_Truseq_Amp_R_12 | CAAGCAGAAGACGGCATACGAGATTACAAGGTGACTGGAGTTCAGACG |  |  |
| PCR_Truseq_Amp_R_13 | CAAGCAGAAGACGGCATACGAGATTTGACTGTGACTGGAGTTCAGACG |  |  |
| PCR_Truseq_Amp_R_14 | CAAGCAGAAGACGGCATACGAGATGGAACTGTGACTGGAGTTCAGACG |  |  |
| PCR_Truseq_Amp_R_15 | CAAGCAGAAGACGGCATACGAGATTGACATGTGACTGGAGTTCAGACG |  |  |
| PCR_Truseq_Amp_R_16 | CAAGCAGAAGACGGCATACGAGATGGACGGGTGACTGGAGTTCAGACG |  |  |
| PCR_Truseq_Amp_R_18 | CAAGCAGAAGACGGCATACGAGATGCGGACGTGACTGGAGTTCAGACG |  |  |
| PCR_Truseq_Amp_R_19 | CAAGCAGAAGACGGCATACGAGATTTTCACGTGACTGGAGTTCAGACG |  |  |
| PCR_Truseq_Amp_R_20 | CAAGCAGAAGACGGCATACGAGATGGCCACGTGACTGGAGTTCAGACG |  |  |
| PCR_Truseq_Amp_R_21 | CAAGCAGAAGACGGCATACGAGATCGAAACGTGACTGGAGTTCAGACG |  |  |
| PCR_Truseq_Amp_R_22 | CAAGCAGAAGACGGCATACGAGATCGTACGGTGACTGGAGTTCAGACG |  |  |
| PCR_Truseq_Amp_R_23 | CAAGCAGAAGACGGCATACGAGATCCACTCGTGACTGGAGTTCAGACG |  |  |
| PCR_Truseq_Amp_R_25 | CAAGCAGAAGACGGCATACGAGATATCAGTGTGACTGGAGTTCAGACG |  |  |
| PCR_Truseq_Amp_R_27 | CAAGCAGAAGACGGCATACGAGATAGGAATGTGACTGGAGTTCAGACG |  |  |
|  |  |  |  |
| pQT4MiF | ctctttccctacacgacgctcttccgatctCCATAGCATGACCGACACAT | Paired to amplify *Q* gene promoter in cv. Bobwhite | Amplifying the *Q* gene promoter to confirm the sequence using Sanger sequencing. |
| pQT17MiR | ctggagttcagacgtgtgctcttccgatctCCACAGGCGTACCTGTCC |  |  |
| pQT17MiF | ctctttccctacacgacgctcttccgatctGTGACCACGAGCGTAAGGTT | Paired to amplify *Q* gene promoter in cv. Bobwhite |  |
| pQT25MiR | ctggagttcagacgtgtgctcttccgatctAGTGGCATGGACAAAGAACC |  |  |
| pQT25checkF | TGCACTTGGGCAATGTAATG | Paired to amplify *Q* gene promoter in cv. Bobwhite |  |
| Q5endR3 | GGAGCAGTCGTCATCATCAG |  |  |
| M13-F | CGCCAGGGTTTTCCCAGTCACGAC |  | Sanger sequencing of *Q* gene promoter region. |
| M13R | AGCGGATAACAATTTCACACAGGA |  |  |
| pQT25checkF | TGCACTTGGGCAATGTAATG |  |  |
| pQT25checkR | AGTGGCATGGACAAAGAACC |  |  |
| pQT26checkF | AGGAGGCATTTAGCTTGTGGAG |  |  |
| pQT26checkR | GCCAGTGGGAGAAAGAGAAAGA |  |  |
| Q5endR3 | GGAGCAGTCGTCATCATCAG |  |  |
|  |  |  |  |
| pQT17checkF | GTGACCACGAGCGTAAGGTT | The Cas9 target flanking primers without truseq adapter tails make it eaier to amplify long fragment from wheat genomic DNA. | Deletion detection on Q gene promoter in M1 progeny inoculated by BSMV-pQT. |
| pQT17checkR | CCACAGGCGTACCTGTCC |  |  |
| pQT18checkF | AAGGAACAACGTTTCCGATG |  |  |
| pQT18checkR | CGCTCTCTTTCGTGTGTGTG |  |  |
| pQT23checkF | AATTGCACCCATCCCATTAC |  |  |
| pQT23checkR | AGGCCTCGTGTGTTGACC |  |  |
| pQT25checkF | TGCACTTGGGCAATGTAATG |  |  |
| pQT25checkR | AGTGGCATGGACAAAGAACC |  |  |
| pQT26checkF | AGGAGGCATTTAGCTTGTGGAG |  |  |
| pQT26checkR | GCCAGTGGGAGAAAGAGAAAGA |  |  |

**Table S2.** The target sites selected for BSMV-sgRNA-based editing in this study.

| Gene targeted | Target name¶ | Spacer Sequence | PAM | Strand^‡^ targeted |
| --- | --- | --- | --- | --- |
| *Q* Gene ID^†^: TraesCS5A02G473800 | QT1 | ATGAGGAACTGGACCAAGG | AGG | Plus |
|  | pQT17 | GTTTCCGATGGGGCGAGCGG | GGG | Plus |
|  | pQT18 | GACGTAGACCCACGCGAGGG | CGG | Minus |
|  | pQT23 | CGTGCCGACGAGAGCACGGG | GGG | Minus |
|  | pQT25 | AGCACAGGGCGTACGGCCGG | GGG | Plus |
|  | pQT26 | CGCTACGGCCACACACCCCG | TGG | Minus |
| *TaGW7* Gene ID: TraesCS2A02G176000 TraesCS2B02G202300 TraesCS2D02G183400 | GW7T6 | TCCATCAACCGGGACTCGGG | AGG | Plus |
| *TaGW2* Gene ID: TraesCS6A02G189300  TraesCS6B02G215300  TraesCS6D02G176900 | GW2T2 | GCTCGCGCCCTGCTACCCGG | GGG | Plus |
| *TaMTL* Gene ID: TraesCS4A02G018100 TraesCS4B02G286000 TraesCS4D02G284700 | MTLT5 | GCATCCGCTCGCCGATCCTG | AGG | Minus |
| TaUPL3^§^ *Gene ID*: TraesCS2A02G064700 TraesCS2B02G076900 | UPL3T11 | CAAGGAGCAGCAGGAGCCCT | CGG | Plus |
|  |  |  |  |  |
|  |  |  |  |  |
|  |  |  |  |  |
| † : The gene IDs are based on the IWGSC RefSeq v1.1 gene annotation. | | |  |  |
| ‡: The plus strand stand for the direction from start codon to the stop codon of a gene. | | |  |  |
| §: The Chromosome 2D genome copy of TaUPL3 is in the region of chr2D:26764500-26774638, which was not annotated. But the D genome copy is functional protein coding gene. | | | | |
| ¶: All the CRISPR-Cas9 targets targeting the coding region of the genes except for the targets start with lower case letter p which target the promoter region of *Q* gene. | | | | |

**Table S3.** The efficiency of the *Q* gene (chromosome 5A) editing based on the BSMV-sgRNA delivery system in transgenic wheat lines with the low (7438) and high (C413) levels of Cas9 expression.

| **Plants and Constructs** | **Genome** | **Mutated Reads** | **Total Reads** | **Mutated Percentage** | **Average** |
| --- | --- | --- | --- | --- | --- |
| 7438-1-3-14 BSMV-QT1 | A | 99 | 17104 | 0.58% | 0.57% |
|  | B | 85 | 18766 | 0.45% |  |
|  | D | 128 | 18617 | 0.69% |  |
| 7438-1-7-1 BSMV-QT1 | A | 145 | 43415 | 0.33% | 0.33% |
|  | B | 165 | 47613 | 0.35% |  |
|  | D | 163 | 52476 | 0.31% |  |
| 7438-1-7-2 BSMV-QT1 | A | 65 | 17375 | 0.37% | 0.38% |
|  | B | 82 | 19484 | 0.42% |  |
|  | D | 73 | 21206 | 0.34% |  |
| C413-1-12-47 BSMV-QT1 | A | 268 | 274 | 97.81% | 98.50% |
|  | B | 419 | 427 | 98.13% |  |
|  | D | 499 | 503 | 99.20% |  |
| C413-1-12-116 BSMV-QT1 | A | 381 | 385 | 98.96% | 99.03% |
|  | B | 400 | 404 | 99.01% |  |
|  | D | 446 | 450 | 99.11% |  |
| C413-1-12-117 BSMV-QT1 | A | 132 | 138 | 95.65% | 96.14% |
|  | B | 162 | 164 | 98.78% |  |
|  | D | 204 | 216 | 94.44% |  |
| C413-1-12-118 BSMV-QT1 | A | 64 | 64 | 100.00% | 99.58% |
|  | B | 65 | 66 | 98.48% |  |
|  | D | 107 | 107 | 100.00% |  |
| C413-1-12-122 BSMV-QT1 | A | 32 | 32 | 100.00% | 96.15% |
|  | B | 48 | 48 | 100.00% |  |
|  | D | 70 | 76 | 92.11% |  |
| C413-1-12-126 BSMV-PDS | A | 0 | 92 | 0.00% | 0.00% |
|  | B | 0 | 124 | 0.00% |  |
|  | D | 0 | 104 | 0.00% |  |
| C413-1-12-128 BSMV-PDS | A | 0 | 330 | 0.00% | 0.00% |
|  | B | 0 | 337 | 0.00% |  |
|  | D | 0 | 454 | 0.00% |  |

**Table S4.** The efficiency of somatic editing in the wheat leaves inoculated with the BSMV transcripts carrying the TaGW2 gRNA with and without mobile elements.

| **Plant ID** | **Inoculation** | **Genome** | **Mutated Reads** | **Total Reads** | **Mutated Percentage** | **Mean** |
| --- | --- | --- | --- | --- | --- | --- |
| C413-1-1-80 | FES | A | 38 | 4827 | 0.79% | 0.76% |
|  |  | B | 32 | 2829 | 1.13% |  |
|  |  | D | 23 | 4622 | 0.50% |  |
| C413-1-1-76 | FES | A | 22 | 3917 | 0.56% | 0.80% |
|  |  | B | 29 | 2169 | 1.34% |  |
|  |  | D | 27 | 3684 | 0.73% |  |
| C413-1-1-84 | FES | A | 37 | 5591 | 0.66% | 0.64% |
|  |  | B | 23 | 2871 | 0.80% |  |
|  |  | D | 29 | 5456 | 0.53% |  |
| C413-1-1-88 | FES | A | 62 | 5455 | 1.14% | 0.81% |
|  |  | B | 19 | 2905 | 0.65% |  |
|  |  | D | 29 | 5243 | 0.55% |  |
| C413-1-1-92 | FES | A | 70 | 5225 | 1.34% | 1.23% |
|  |  | B | 47 | 2621 | 1.79% |  |
|  |  | D | 40 | 4923 | 0.81% |  |
| C413-1-1-91 | BSMV | A | 43 | 4366 | 0.98% | 0.74% |
|  |  | B | 18 | 2474 | 0.73% |  |
|  |  | D | 21 | 4235 | 0.50% |  |
| C413-1-1-79 | BSMV | A | 55 | 5541 | 0.99% | 0.84% |
|  |  | B | 24 | 2635 | 0.91% |  |
|  |  | D | 36 | 5438 | 0.66% |  |
| C413-1-1-83 | BSMV | A | 70 | 4619 | 1.52% | 1.19% |
|  |  | B | 21 | 2445 | 0.86% |  |
|  |  | D | 48 | 4592 | 1.05% |  |
| C413-1-1-87 | BSMV | A | 64 | 5154 | 1.24% | 1.28% |
|  |  | B | 62 | 3044 | 2.04% |  |
|  |  | D | 43 | 5016 | 0.86% |  |
| C413-1-1-96 | BSMV | A | 67 | 4276 | 1.57% | 1.95% |
|  |  | B | 105 | 2277 | 4.61% |  |
|  |  | D | 38 | 4241 | 0.90% |  |
| C413-1-1-75 | PDS | A | 65 | 4171 | 1.56% | 1.88% |
|  |  | B | 106 | 3799 | 2.79% |  |
|  |  | D | 59 | 4252 | 1.39% |  |
| C413-1-1-1 | PDS | A | 47 | 4721 | 1.00% | 0.81% |
|  |  | B | 8 | 2630 | 0.30% |  |
|  |  | D | 41 | 4555 | 0.90% |  |
| C413-1-1-71 | PDS | A | 35 | 5368 | 0.65% | 0.79% |
|  |  | B | 18 | 3196 | 0.56% |  |
|  |  | D | 53 | 4825 | 1.10% |  |
| C413-1-1-72 | PDS | A | 30 | 4527 | 0.66% | 0.52% |
|  |  | B | 21 | 2886 | 0.73% |  |
|  |  | D | 11 | 4455 | 0.25% |  |
| C413-1-1-73 | PDS | A | 36 | 4303 | 0.84% | 1.58% |
|  |  | B | 52 | 2141 | 2.43% |  |
|  |  | D | 83 | 4375 | 1.90% |  |
| C413-1-1-77 | PDS | A | 25 | 4032 | 0.62% | 0.66% |
|  |  | B | 18 | 2443 | 0.74% |  |
|  |  | D | 25 | 3807 | 0.66% |  |
| C413-1-1-78 | PDS | A | 9 | 2971 | 0.30% | 0.26% |
|  |  | B | 8 | 2376 | 0.34% |  |
|  |  | D | 5 | 3118 | 0.16% |  |
| C413-1-1-86 | PDS | A | 148 | 3554 | 4.16% | 3.85% |
|  |  | B | 90 | 2323 | 3.87% |  |
|  |  | D | 119 | 3398 | 3.50% |  |
| C413-1-1-89 | PDS | A | 28 | 5468 | 0.51% | 0.52% |
|  |  | B | 24 | 3317 | 0.72% |  |
|  |  | D | 23 | 5626 | 0.41% |  |
| C413-1-1-90 | PDS | A | 26 | 5935 | 0.44% | 0.76% |
|  |  | B | 37 | 3525 | 1.05% |  |
|  |  | D | 52 | 5727 | 0.91% |  |
| C413-1-1-85 | PDS | A | 27 | 5429 | 0.50% | 0.43% |
|  |  | B | 7 | 2121 | 0.33% |  |
|  |  | D | 20 | 5150 | 0.39% |  |
| C413-1-1-95 | PDS | A | 63 | 5685 | 1.11% | 1.06% |
|  |  | B | 55 | 3110 | 1.77% |  |
|  |  | D | 36 | 5766 | 0.62% |  |
| C413-1-1-6 | AtFT | A | 3845 | 4446 | 86.48% | 84.54% |
|  |  | B | 1899 | 2367 | 80.23% |  |
|  |  | D | 3411 | 4016 | 84.94% |  |
| C413-1-1-2 | AtFT | A | 76 | 5867 | 1.30% | 1.54% |
|  |  | B | 72 | 2842 | 2.53% |  |
|  |  | D | 74 | 5709 | 1.30% |  |
| C413-1-1-3 | AtFT | A | 2086 | 3479 | 59.96% | 59.71% |
|  |  | B | 1928 | 3360 | 57.38% |  |
|  |  | D | 2204 | 3574 | 61.67% |  |
| C413-1-1-4 | AtFT | A | 279 | 1418 | 19.68% | 18.45% |
|  |  | B | 166 | 852 | 19.48% |  |
|  |  | D | 260 | 1552 | 16.75% |  |
| C413-1-1-8 | AtFT | A | 49 | 4808 | 1.02% | 0.91% |
|  |  | B | 38 | 4083 | 0.93% |  |
|  |  | D | 37 | 4755 | 0.78% |  |
| C413-1-1-9 | AtFT | A | 1060 | 5340 | 19.85% | 19.80% |
|  |  | B | 782 | 3581 | 21.84% |  |
|  |  | D | 1020 | 5530 | 18.44% |  |
| C413-1-1-11 | AtFT | A | 1548 | 4853 | 31.90% | 29.43% |
|  |  | B | 919 | 2920 | 31.47% |  |
|  |  | D | 1204 | 4702 | 25.61% |  |
| C413-1-1-21 | AtFT | A | 2214 | 5214 | 42.46% | 41.61% |
|  |  | B | 1222 | 3119 | 39.18% |  |
|  |  | D | 2043 | 4836 | 42.25% |  |
| C413-1-1-26 | AtFT | A | 2745 | 4271 | 64.27% | 60.68% |
|  |  | B | 1574 | 2675 | 58.84% |  |
|  |  | D | 2358 | 4058 | 58.11% |  |
| C413-1-1-27 | AtFT | A | 1481 | 4650 | 31.85% | 29.60% |
|  |  | B | 776 | 2707 | 28.67% |  |
|  |  | D | 1288 | 4621 | 27.87% |  |
| C413-1-1-32 | AtFT | A | 39 | 3506 | 1.11% | 1.11% |
|  |  | B | 42 | 2046 | 2.05% |  |
|  |  | D | 17 | 3255 | 0.52% |  |
| C413-1-1-33 | AtFT | A | 124 | 4350 | 2.85% | 3.06% |
|  |  | B | 60 | 2829 | 2.12% |  |
|  |  | D | 161 | 4091 | 3.94% |  |
| C413-1-1-5 | Vrn3 | A | 3034 | 3681 | 82.42% | 83.11% |
|  |  | B | 1694 | 2016 | 84.03% |  |
|  |  | D | 2905 | 3487 | 83.31% |  |
| C413-1-1-10 | Vrn3 | A | 1427 | 5078 | 28.10% | 27.54% |
|  |  | B | 598 | 2413 | 24.78% |  |
|  |  | D | 1334 | 4707 | 28.34% |  |
| C413-1-1-12 | Vrn3 | A | 3462 | 4069 | 85.08% | 83.45% |
|  |  | B | 1903 | 2330 | 81.67% |  |
|  |  | D | 3529 | 4259 | 82.86% |  |
| C413-1-1-14 | Vrn3 | A | 123 | 4483 | 2.74% | 1.99% |
|  |  | B | 38 | 2916 | 1.30% |  |
|  |  | D | 74 | 4439 | 1.67% |  |
| C413-1-1-15 | Vrn3 | A | 1137 | 4354 | 26.11% | 25.95% |
|  |  | B | 684 | 2611 | 26.20% |  |
|  |  | D | 1157 | 4509 | 25.66% |  |
| C413-1-1-17 | Vrn3 | A | 83 | 4701 | 1.77% | 1.40% |
|  |  | B | 46 | 2978 | 1.54% |  |
|  |  | D | 41 | 4428 | 0.93% |  |
| C413-1-1-19 | Vrn3 | A | 380 | 3786 | 10.04% | 7.93% |
|  |  | B | 143 | 2156 | 6.63% |  |
|  |  | D | 235 | 3614 | 6.50% |  |
| C413-1-1-20 | Vrn3 | A | 521 | 2870 | 18.15% | 17.86% |
|  |  | B | 382 | 2080 | 18.37% |  |
|  |  | D | 488 | 2839 | 17.19% |  |
| C413-1-1-22 | Vrn3 | A | 3629 | 4646 | 78.11% | 73.88% |
|  |  | B | 1628 | 2419 | 67.30% |  |
|  |  | D | 3211 | 4397 | 73.03% |  |
| C413-1-1-23 | Vrn3 | A | 104 | 6536 | 1.59% | 1.88% |
|  |  | B | 96 | 3235 | 2.97% |  |
|  |  | D | 94 | 5894 | 1.59% |  |
| C413-1-1-28 | Vrn3 | A | 2041 | 4346 | 46.96% | 44.13% |
|  |  | B | 1090 | 2572 | 42.38% |  |
|  |  | D | 1707 | 4045 | 42.20% |  |
| C413-1-1-34 | Vrn3 | A | 1052 | 4741 | 22.19% | 21.71% |
|  |  | B | 601 | 2618 | 22.96% |  |
|  |  | D | 912 | 4457 | 20.46% |  |
| C413-1-1-38 | tRNAmet | A | 3052 | 3442 | 88.67% | 88.44% |
|  |  | B | 1897 | 2098 | 90.42% |  |
|  |  | D | 2976 | 3421 | 86.99% |  |
| C413-1-1-18 | tRNAmet | A | 1853 | 1999 | 92.70% | 91.28% |
|  |  | B | 1019 | 1157 | 88.07% |  |
|  |  | D | 1764 | 1923 | 91.73% |  |
| C413-1-1-24 | tRNAmet | A | 3966 | 4158 | 95.38% | 94.27% |
|  |  | B | 2486 | 2652 | 93.74% |  |
|  |  | D | 3727 | 3988 | 93.46% |  |
| C413-1-1-29 | tRNAmet | A | 1948 | 3987 | 48.86% | 47.47% |
|  |  | B | 1110 | 2495 | 44.49% |  |
|  |  | D | 1750 | 3647 | 47.98% |  |
| C413-1-1-30 | tRNAmet | A | 3949 | 4156 | 95.02% | 93.49% |
|  |  | B | 2429 | 2589 | 93.82% |  |
|  |  | D | 3290 | 3596 | 91.49% |  |
| C413-1-1-35 | tRNAmet | A | 2675 | 4696 | 56.96% | 55.02% |
|  |  | B | 1530 | 3037 | 50.38% |  |
|  |  | D | 2514 | 4480 | 56.12% |  |
| C413-1-1-36 | tRNAmet | A | 1324 | 3645 | 36.32% | 32.22% |
|  |  | B | 890 | 2540 | 35.04% |  |
|  |  | D | 938 | 3599 | 26.06% |  |
| C413-1-1-37 | tRNAmet | A | 4343 | 4476 | 97.03% | 96.40% |
|  |  | B | 2663 | 2770 | 96.14% |  |
|  |  | D | 4320 | 4503 | 95.94% |  |
| C413-1-1-39 | tRNAmet | A | 3827 | 4230 | 90.47% | 91.25% |
|  |  | B | 2601 | 2826 | 92.04% |  |
|  |  | D | 3806 | 4159 | 91.51% |  |
| C413-1-1-40 | tRNAmet | A | 4213 | 4453 | 94.61% | 93.81% |
|  |  | B | 2475 | 2681 | 92.32% |  |
|  |  | D | 4208 | 4481 | 93.91% |  |
| C413-1-1-44 | tRNAmet | A | 3301 | 3730 | 88.50% | 89.17% |
|  |  | B | 2419 | 2700 | 89.59% |  |
|  |  | D | 3025 | 3377 | 89.58% |  |
| C413-1-1-45 | tRNAmet | A | 1023 | 2978 | 34.35% | 33.58% |
|  |  | B | 817 | 2181 | 37.46% |  |
|  |  | D | 780 | 2643 | 29.51% |  |
| C413-1-1-42 | tRNAIle | A | 59 | 3997 | 1.48% | 1.45% |
|  |  | B | 58 | 3273 | 1.77% |  |
|  |  | D | 45 | 3892 | 1.16% |  |
| C413-1-1-41 | tRNAIle | A | 3220 | 5612 | 57.38% | 55.88% |
|  |  | B | 1548 | 2818 | 54.93% |  |
|  |  | D | 2908 | 5307 | 54.80% |  |
| C413-1-1-43 | tRNAIle | A | 140 | 4332 | 3.23% | 2.08% |
|  |  | B | 46 | 3223 | 1.43% |  |
|  |  | D | 57 | 4146 | 1.37% |  |
| C413-1-1-46 | tRNAIle | A | 60 | 4565 | 1.31% | 1.55% |
|  |  | B | 15 | 2573 | 0.58% |  |
|  |  | D | 107 | 4605 | 2.32% |  |
| C413-1-1-49 | tRNAIle | A | 17 | 2519 | 0.67% | 1.34% |
|  |  | B | 44 | 1737 | 2.53% |  |
|  |  | D | 32 | 2659 | 1.20% |  |
| C413-1-1-54 | tRNAIle | A | 16 | 4701 | 0.34% | 0.53% |
|  |  | B | 21 | 2725 | 0.77% |  |
|  |  | D | 28 | 4734 | 0.59% |  |
| C413-1-1-55 | tRNAIle | A | 14 | 2228 | 0.63% | 0.46% |
|  |  | B | 4 | 1416 | 0.28% |  |
|  |  | D | 9 | 2228 | 0.40% |  |
| C413-1-1-61 | tRNAIle | A | 23 | 1559 | 1.48% | 0.95% |
|  |  | B | 9 | 1103 | 0.82% |  |
|  |  | D | 7 | 1462 | 0.48% |  |
| C413-1-1-67 | tRNAIle | A | 116 | 4474 | 2.59% | 2.75% |
|  |  | B | 106 | 2178 | 4.87% |  |
|  |  | D | 79 | 4292 | 1.84% |  |
| C413-1-1-68 | tRNAIle | A | 21 | 4195 | 0.50% | 0.60% |
|  |  | B | 17 | 2685 | 0.63% |  |
|  |  | D | 30 | 4436 | 0.68% |  |
| C413-1-1-70 | tRNAIle | A | 61 | 4780 | 1.28% | 1.22% |
|  |  | B | 29 | 2795 | 1.04% |  |
|  |  | D | 58 | 4524 | 1.28% |  |
| C413-1-1-69 | tRNAIle | A | 87 | 3998 | 2.18% | 1.50% |
|  |  | B | 29 | 2296 | 1.26% |  |
|  |  | D | 33 | 3634 | 0.91% |  |
| C413-1-1-53 | GW2T2 | A | 2783 | 4400 | 63.25% | 59.93% |
|  |  | B | 1467 | 2571 | 57.06% |  |
|  |  | D | 2434 | 4182 | 58.20% |  |
| C413-1-1-51 | GW2T2 | A | 2049 | 4010 | 51.10% | 50.68% |
|  |  | B | 1316 | 2611 | 50.40% |  |
|  |  | D | 1852 | 3674 | 50.41% |  |
| C413-1-1-52 | GW2T2 | A | 42 | 4468 | 0.94% | 0.95% |
|  |  | B | 21 | 2441 | 0.86% |  |
|  |  | D | 43 | 4210 | 1.02% |  |
| C413-1-1-56 | GW2T2 | A | 2658 | 3810 | 69.76% | 64.61% |
|  |  | B | 1333 | 2191 | 60.84% |  |
|  |  | D | 2310 | 3752 | 61.57% |  |
| C413-1-1-57 | GW2T2 | A | 5029 | 5234 | 96.08% | 95.22% |
|  |  | B | 2440 | 2616 | 93.27% |  |
|  |  | D | 4339 | 4551 | 95.34% |  |
| C413-1-1-58 | GW2T2 | A | 4644 | 4964 | 93.55% | 93.02% |
|  |  | B | 2783 | 2990 | 93.08% |  |
|  |  | D | 4581 | 4955 | 92.45% |  |
| C413-1-1-59 | GW2T2 | A | 3733 | 3989 | 93.58% | 94.22% |
|  |  | B | 2492 | 2623 | 95.01% |  |
|  |  | D | 3687 | 3908 | 94.34% |  |
| C413-1-1-60 | GW2T2 | A | 3458 | 3582 | 96.54% | 96.55% |
|  |  | B | 2229 | 2309 | 96.54% |  |
|  |  | D | 3393 | 3513 | 96.58% |  |
| C413-1-1-62 | GW2T2 | A | 2989 | 3234 | 92.42% | 93.10% |
|  |  | B | 2586 | 2744 | 94.24% |  |
|  |  | D | 3178 | 3424 | 92.82% |  |
| C413-1-1-63 | GW2T2 | A | 2710 | 2829 | 95.79% | 94.94% |
|  |  | B | 1636 | 1720 | 95.12% |  |
|  |  | D | 2533 | 2697 | 93.92% |  |
| C413-1-1-64 | GW2T2 | A | 3624 | 3940 | 91.98% | 91.88% |
|  |  | B | 2657 | 2895 | 91.78% |  |
|  |  | D | 3433 | 3738 | 91.84% |  |
| C413-1-1-65 | GW2T2 | A | 3278 | 3603 | 90.98% | 90.07% |
|  |  | B | 1966 | 2221 | 88.52% |  |
|  |  | D | 3140 | 3484 | 90.13% |  |

**Table S5.** Somatic editing efficiency of individual and pooled (GUG) BSMV transcripts.

| **Plant ID** | **Inoculation** | **Sequenced_target** | **Genome** | **Mutated Reads** | **Total Reads** | **Mutated Percentage** | **Mean** |
| --- | --- | --- | --- | --- | --- | --- | --- |
| C413-1-12-38 | BSMV-GUG | GW2T2 | A | 1745 | 4409 | 39.58% | 37.70% |
|  |  |  | B | 1329 | 3772 | 35.23% |  |
|  |  |  | D | 1610 | 4245 | 37.93% |  |
| C413-1-12-44 | BSMV-GUG | GW2T2 | A | 1860 | 3639 | 51.11% | 48.22% |
|  |  |  | B | 1260 | 2905 | 43.37% |  |
|  |  |  | D | 1702 | 3457 | 49.23% |  |
| C413-1-12-41 | BSMV-GUG | GW2T2 | A | 2397 | 5221 | 45.91% | 36.48% |
|  |  |  | B | 1072 | 3942 | 27.19% |  |
|  |  |  | D | 1748 | 5138 | 34.02% |  |
| C413-1-12-48 | BSMV-GUG | GW2T2 | A | 1549 | 4806 | 32.23% | 30.68% |
|  |  |  | B | 1069 | 3829 | 27.92% |  |
|  |  |  | D | 1440 | 4590 | 31.37% |  |
| C413-1-12-61 | BSMV-GUG | GW2T2 | A | 2098 | 4061 | 51.66% | 48.96% |
|  |  |  | B | 1526 | 3403 | 44.84% |  |
|  |  |  | D | 1953 | 3926 | 49.75% |  |
| C413-1-12-66 | BSMV-GUG | GW2T2 | A | 1306 | 3641 | 35.87% | 35.11% |
|  |  |  | B | 1053 | 3010 | 34.98% |  |
|  |  |  | D | 1212 | 3520 | 34.43% |  |
| C413-1-12-68 | BSMV-GUG | GW2T2 | A | 1264 | 5176 | 24.42% | 22.33% |
|  |  |  | B | 851 | 4154 | 20.49% |  |
|  |  |  | D | 1109 | 5110 | 21.70% |  |
| C413-1-12-86 | BSMV-GUG | GW2T2 | A | 1636 | 2902 | 56.37% | 53.96% |
|  |  |  | B | 1244 | 2387 | 52.12% |  |
|  |  |  | D | 1368 | 2583 | 52.96% |  |
| C413-1-12-72 | BSMV-GUG | GW2T2 | A | 376 | 3986 | 9.43% | 8.24% |
|  |  |  | B | 193 | 3214 | 6.00% |  |
|  |  |  | D | 331 | 3724 | 8.89% |  |
| C413-1-12-69 | BSMV-GUG | GW2T2 | A | 158 | 4365 | 3.62% | 2.92% |
|  |  |  | B | 89 | 3838 | 2.32% |  |
|  |  |  | D | 120 | 4363 | 2.75% |  |
| C413-1-12-125 | BSMV-GW2T2 | GW2T2 | A | 3230 | 3870 | 83.46% | 83.63% |
|  |  |  | B | 2676 | 3179 | 84.18% |  |
|  |  |  | D | 3134 | 3760 | 83.35% |  |
| C413-1-12-106 | BSMV-GW2T2 | GW2T2 | A | 5348 | 5590 | 95.67% | 95.42% |
|  |  |  | B | 3875 | 4078 | 95.02% |  |
|  |  |  | D | 5270 | 5521 | 95.45% |  |
| C413-1-12-105 | BSMV-GW2T2 | GW2T2 | A | 4100 | 4441 | 92.32% | 92.39% |
|  |  |  | B | 3555 | 3817 | 93.14% |  |
|  |  |  | D | 3914 | 4264 | 91.79% |  |
| C413-1-12-63 | BSMV-GW2T2 | GW2T2 | A | 5004 | 5351 | 93.52% | 93.88% |
|  |  |  | B | 3576 | 3803 | 94.03% |  |
|  |  |  | D | 4672 | 4962 | 94.16% |  |
| C413-1-12-104 | BSMV-GW2T2 | GW2T2 | A | 4309 | 4642 | 92.83% | 92.37% |
|  |  |  | B | 4086 | 4464 | 91.53% |  |
|  |  |  | D | 4045 | 4361 | 92.75% |  |
| C413-1-12-99 | BSMV-GW2T2 | GW2T2 | A | 4376 | 4643 | 94.25% | 94.63% |
|  |  |  | B | 3607 | 3788 | 95.22% |  |
|  |  |  | D | 4277 | 4525 | 94.52% |  |
| C413-1-12-102 | BSMV-GW2T2 | GW2T2 | A | 4209 | 4493 | 93.68% | 93.64% |
|  |  |  | B | 3315 | 3527 | 93.99% |  |
|  |  |  | D | 4113 | 4407 | 93.33% |  |
| C413-1-12-98 | BSMV-GW2T2 | GW2T2 | A | 3416 | 3731 | 91.56% | 91.54% |
|  |  |  | B | 2897 | 3137 | 92.35% |  |
|  |  |  | D | 4661 | 5120 | 91.04% |  |
| C413-1-12-101 | BSMV-GW2T2 | GW2T2 | A | 1572 | 1684 | 93.35% | 93.78% |
|  |  |  | B | 1224 | 1311 | 93.36% |  |
|  |  |  | D | 1623 | 1717 | 94.53% |  |
| C413-1-12-129 | BSMV-GW2T2 | GW2T2 | A | 4089 | 4903 | 83.40% | 81.84% |
|  |  |  | B | 3723 | 4610 | 80.76% |  |
|  |  |  | D | 2133 | 2639 | 80.83% |  |
| C413-1-12-38 | BSMV-GUG | UPL3T11 | A | 485 | 2976 | 16.30% | 19.04% |
|  |  |  | B | 444 | 2121 | 20.93% |  |
|  |  |  | D | 593 | 2898 | 20.46% |  |
| C413-1-12-44 | BSMV-GUG | UPL3T11 | A | 473 | 3123 | 15.15% | 19.55% |
|  |  |  | B | 505 | 2353 | 21.46% |  |
|  |  |  | D | 718 | 3198 | 22.45% |  |
| C413-1-12-41 | BSMV-GUG | UPL3T11 | A | 543 | 4444 | 12.22% | 16.77% |
|  |  |  | B | 733 | 3361 | 21.81% |  |
|  |  |  | D | 750 | 4279 | 17.53% |  |
| C413-1-12-48 | BSMV-GUG | UPL3T11 | A | 1108 | 5050 | 21.94% | 27.37% |
|  |  |  | B | 1115 | 3783 | 29.47% |  |
|  |  |  | D | 1534 | 4893 | 31.35% |  |
| C413-1-12-61 | BSMV-GUG | UPL3T11 | A | 1631 | 8570 | 19.03% | 21.91% |
|  |  |  | B | 1485 | 7262 | 20.45% |  |
|  |  |  | D | 2276 | 8783 | 25.91% |  |
| C413-1-12-66 | BSMV-GUG | UPL3T11 | A | 989 | 5683 | 17.40% | 22.80% |
|  |  |  | B | 948 | 4087 | 23.20% |  |
|  |  |  | D | 1563 | 5580 | 28.01% |  |
| C413-1-12-68 | BSMV-GUG | UPL3T11 | A | 217 | 2247 | 9.66% | 12.89% |
|  |  |  | B | 257 | 1768 | 14.54% |  |
|  |  |  | D | 322 | 2159 | 14.91% |  |
| C413-1-12-86 | BSMV-GUG | UPL3T11 | A | 548 | 5071 | 10.81% | 16.35% |
|  |  |  | B | 714 | 4048 | 17.64% |  |
|  |  |  | D | 1067 | 5129 | 20.80% |  |
| C413-1-12-72 | BSMV-GUG | UPL3T11 | A | 56 | 2169 | 2.58% | 3.61% |
|  |  |  | B | 68 | 1599 | 4.25% |  |
|  |  |  | D | 89 | 2125 | 4.19% |  |
| C413-1-12-69 | BSMV-GUG | UPL3T11 | A | 229 | 1755 | 13.05% | 19.78% |
|  |  |  | B | 291 | 1489 | 19.54% |  |
|  |  |  | D | 491 | 1866 | 26.31% |  |
| C413-1-12-76 | BSMV-UPL3T11 | UPL3T11 | A | 644 | 5385 | 11.96% | 16.98% |
|  |  |  | B | 723 | 3806 | 19.00% |  |
|  |  |  | D | 1090 | 5279 | 20.65% |  |
| C413-1-12-92 | BSMV-UPL3T11 | UPL3T11 | A | 660 | 4965 | 13.29% | 18.28% |
|  |  |  | B | 723 | 3586 | 20.16% |  |
|  |  |  | D | 1092 | 4990 | 21.88% |  |
| C413-1-12-97 | BSMV-UPL3T11 | UPL3T11 | A | 869 | 4322 | 20.11% | 23.95% |
|  |  |  | B | 778 | 3161 | 24.61% |  |
|  |  |  | D | 1161 | 4239 | 27.39% |  |
| C413-1-12-94 | BSMV-UPL3T11 | UPL3T11 | A | 589 | 3812 | 15.45% | 20.40% |
|  |  |  | B | 541 | 2883 | 18.77% |  |
|  |  |  | D | 1030 | 3892 | 26.46% |  |
| C413-1-12-96 | BSMV-UPL3T11 | UPL3T11 | A | 385 | 3641 | 10.57% | 15.58% |
|  |  |  | B | 510 | 2979 | 17.12% |  |
|  |  |  | D | 729 | 3803 | 19.17% |  |
| C413-1-12-38 | BSMV-GUG | GW7T6 | A | 88 | 125 | 70.40% | 79.95% |
|  |  |  | B | 348 | 414 | 84.06% |  |
|  |  |  | D | 270 | 344 | 78.49% |  |
| C413-1-12-44 | BSMV-GUG | GW7T6 | A | 121 | 228 | 53.07% | 53.79% |
|  |  |  | B | 390 | 680 | 57.35% |  |
|  |  |  | D | 255 | 516 | 49.42% |  |
| C413-1-12-41 | BSMV-GUG | GW7T6 | A | 130 | 196 | 66.33% | 81.37% |
|  |  |  | B | 483 | 565 | 85.49% |  |
|  |  |  | D | 483 | 586 | 82.42% |  |
| C413-1-12-48 | BSMV-GUG | GW7T6 | A | 150 | 268 | 55.97% | 58.29% |
|  |  |  | B | 443 | 718 | 61.70% |  |
|  |  |  | D | 342 | 618 | 55.34% |  |
| C413-1-12-61 | BSMV-GUG | GW7T6 | A | 239 | 365 | 65.48% | 72.53% |
|  |  |  | B | 457 | 603 | 75.79% |  |
|  |  |  | D | 463 | 630 | 73.49% |  |
| C413-1-12-66 | BSMV-GUG | GW7T6 | A | 111 | 301 | 36.88% | 42.08% |
|  |  |  | B | 319 | 703 | 45.38% |  |
|  |  |  | D | 274 | 669 | 40.96% |  |
| C413-1-12-68 | BSMV-GUG | GW7T6 | A | 114 | 256 | 44.53% | 52.81% |
|  |  |  | B | 290 | 565 | 51.33% |  |
|  |  |  | D | 329 | 567 | 58.02% |  |
| C413-1-12-86 | BSMV-GUG | GW7T6 | A | 104 | 266 | 39.10% | 47.48% |
|  |  |  | B | 317 | 649 | 48.84% |  |
|  |  |  | D | 249 | 496 | 50.20% |  |
| C413-1-12-72 | BSMV-GUG | GW7T6 | A | 261 | 296 | 88.18% | 91.27% |
|  |  |  | B | 483 | 517 | 93.42% |  |
|  |  |  | D | 438 | 482 | 90.87% |  |
| C413-1-12-69 | BSMV-GUG | GW7T6 | A | 88 | 283 | 31.10% | 36.84% |
|  |  |  | B | 193 | 491 | 39.31% |  |
|  |  |  | D | 185 | 491 | 37.68% |  |
| C413-1-12-81 | BSMV-GW7T6 | GW7T6 | A | 207 | 317 | 65.30% | 79.01% |
|  |  |  | B | 577 | 681 | 84.73% |  |
|  |  |  | D | 428 | 536 | 79.85% |  |
| C413-1-12-84 | BSMV-GW7T6 | GW7T6 | A | 192 | 241 | 79.67% | 79.13% |
|  |  |  | B | 436 | 537 | 81.19% |  |
|  |  |  | D | 335 | 439 | 76.31% |  |
| C413-1-12-85 | BSMV-GW7T6 | GW7T6 | A | 243 | 254 | 95.67% | 97.54% |
|  |  |  | B | 560 | 572 | 97.90% |  |
|  |  |  | D | 544 | 555 | 98.02% |  |
| C413-1-12-87 | BSMV-GW7T6 | GW7T6 | A | 149 | 240 | 62.08% | 75.70% |
|  |  |  | B | 413 | 502 | 82.27% |  |
|  |  |  | D | 379 | 501 | 75.65% |  |
| C413-1-12-89 | BSMV-GW7T6 | GW7T6 | A | 99 | 150 | 66.00% | 75.83% |
|  |  |  | B | 295 | 369 | 79.95% |  |
|  |  |  | D | 224 | 296 | 75.68% |  |
| C413-1-12-125 | BSMV-GW2T2 | GW7T6 | A | 0 | 304 | 0.00% | 0.26% |
|  |  |  | B | 2 | 634 | 0.32% |  |
|  |  |  | D | 2 | 577 | 0.35% |  |
| C413-1-12-106 | BSMV-GW2T2 | GW7T6 | A | 0 | 372 | 0.00% | 0.06% |
|  |  |  | B | 1 | 707 | 0.14% |  |
|  |  |  | D | 0 | 656 | 0.00% |  |

**Table S6.** Somatic editing efficiency in the promoter region of the *Q* gene (chr. 5A).

| **Plant ID** | **Inoculation** | **Sequenced target** | **Genome** | **Mutated Reads** | **Total Reads** | **Mutated Percentage** |
| --- | --- | --- | --- | --- | --- | --- |
| C413-1-12-37 | BSMV-pQT17, 18, 23, 25 and 26 mix | pQT17 | A | 3663 | 4646 | 78.84% |
| C413-1-12-34 | BSMV-pQT17, 18, 23, 25 and 26 mix | pQT17 | A | 4824 | 10504 | 45.93% |
| C413-1-12-40 | BSMV-pQT17, 18, 23, 25 and 26 mix | pQT17 | A | 1994 | 8829 | 22.58% |
| C413-1-12-35 | BSMV-pQT17, 18, 23, 25 and 26 mix | pQT17 | A | 1546 | 7786 | 19.86% |
| C413-1-12-28 | BSMV-pQT17, 18, 23, 25 and 26 mix | pQT17 | A | 1980 | 7283 | 27.19% |
| C413-1-12-36 | BSMV-pQT17, 18, 23, 25 and 26 mix | pQT17 | A | 6218 | 6636 | 93.70% |
| C413-1-12-29 | BSMV-pQT17, 18, 23, 25 and 26 mix | pQT17 | A | 538 | 2478 | 21.71% |
| C413-1-12-30 | BSMV-pQT17, 18, 23, 25 and 26 mix | pQT17 | A | 2428 | 5474 | 44.36% |
| C413-1-12-31 | BSMV-pQT17, 18, 23, 25 and 26 mix | pQT17 | A | 1693 | 8308 | 20.38% |
| C413-1-12-32 | BSMV-pQT17, 18, 23, 25 and 26 mix | pQT17 | A | 3761 | 10385 | 36.22% |
| C413-1-12-88 | BSMV-MTLT5 | pQT17 | A | 81 | 5522 | 1.47% |
|  |  |  |  |  |  |  |
| C413-1-12-37 | BSMV-pQT17, 18, 23, 25 and 26 mix | pQT18 | A | 91 | 428 | 21.26% |
| C413-1-12-34 | BSMV-pQT17, 18, 23, 25 and 26 mix | pQT18 | A | 321 | 879 | 36.52% |
| C413-1-12-40 | BSMV-pQT17, 18, 23, 25 and 26 mix | pQT18 | A | 219 | 759 | 28.85% |
| C413-1-12-35 | BSMV-pQT17, 18, 23, 25 and 26 mix | pQT18 | A | 374 | 855 | 43.74% |
| C413-1-12-28 | BSMV-pQT17, 18, 23, 25 and 26 mix | pQT18 | A | 240 | 609 | 39.41% |
| C413-1-12-36 | BSMV-pQT17, 18, 23, 25 and 26 mix | pQT18 | A | 59 | 481 | 12.27% |
| C413-1-12-29 | BSMV-pQT17, 18, 23, 25 and 26 mix | pQT18 | A | 91 | 200 | 45.50% |
| C413-1-12-30 | BSMV-pQT17, 18, 23, 25 and 26 mix | pQT18 | A | 81 | 365 | 22.19% |
| C413-1-12-31 | BSMV-pQT17, 18, 23, 25 and 26 mix | pQT18 | A | 169 | 661 | 25.57% |
| C413-1-12-32 | BSMV-pQT17, 18, 23, 25 and 26 mix | pQT18 | A | 148 | 589 | 25.13% |
| C413-1-12-88 | BSMV-MTLT5 | pQT18 | A | 29 | 602 | 4.82% |
|  |  |  |  |  |  |  |
| C413-1-12-37 | BSMV-pQT17, 18, 23, 25 and 26 mix | pQT23 | A | 861 | 4274 | 20.15% |
| C413-1-12-34 | BSMV-pQT17, 18, 23, 25 and 26 mix | pQT23 | A | 1326 | 7755 | 17.10% |
| C413-1-12-40 | BSMV-pQT17, 18, 23, 25 and 26 mix | pQT23 | A | 1472 | 6634 | 22.19% |
| C413-1-12-35 | BSMV-pQT17, 18, 23, 25 and 26 mix | pQT23 | A | 791 | 6927 | 11.42% |
| C413-1-12-28 | BSMV-pQT17, 18, 23, 25 and 26 mix | pQT23 | A | 1327 | 6637 | 19.99% |
| C413-1-12-36 | BSMV-pQT17, 18, 23, 25 and 26 mix | pQT23 | A | 1930 | 5934 | 32.52% |
| C413-1-12-29 | BSMV-pQT17, 18, 23, 25 and 26 mix | pQT23 | A | 507 | 2273 | 22.31% |
| C413-1-12-30 | BSMV-pQT17, 18, 23, 25 and 26 mix | pQT23 | A | 2867 | 5262 | 54.48% |
| C413-1-12-31 | BSMV-pQT17, 18, 23, 25 and 26 mix | pQT23 | A | 945 | 6024 | 15.69% |
| C413-1-12-32 | BSMV-pQT17, 18, 23, 25 and 26 mix | pQT23 | A | 4469 | 7388 | 60.49% |
| C413-1-12-88 | BSMV-MTLT5 | pQT23 | A | 143 | 5461 | 2.62% |
|  |  |  |  |  |  |  |
| C413-1-12-37 | BSMV-pQT17, 18, 23, 25 and 26 mix | pQT25 | A | 1457 | 9263 | 16.00% |
| C413-1-12-34 | BSMV-pQT17, 18, 23, 25 and 26 mix | pQT25 | A | 1392 | 21333 | 7.00% |
| C413-1-12-40 | BSMV-pQT17, 18, 23, 25 and 26 mix | pQT25 | A | 1239 | 4934 | 25.00% |
| C413-1-12-35 | BSMV-pQT17, 18, 23, 25 and 26 mix | pQT25 | A | 6248 | 17439 | 36.00% |
| C413-1-12-28 | BSMV-pQT17, 18, 23, 25 and 26 mix | pQT25 | A | 1652 | 4477 | 37.00% |
| C413-1-12-36 | BSMV-pQT17, 18, 23, 25 and 26 mix | pQT25 | A | 4966 | 14678 | 34.00% |
| C413-1-12-29 | BSMV-pQT17, 18, 23, 25 and 26 mix | pQT25 | A | 1793 | 5244 | 34.00% |
| C413-1-12-30 | BSMV-pQT17, 18, 23, 25 and 26 mix | pQT25 | A | 3452 | 12789 | 27.00% |
| C413-1-12-31 | BSMV-pQT17, 18, 23, 25 and 26 mix | pQT25 | A | 2225 | 6324 | 35.00% |
| C413-1-12-32 | BSMV-pQT17, 18, 23, 25 and 26 mix | pQT25 | A | 7473 | 16708 | 45.00% |
| C413-1-12-88 | BSMV-MTLT5 | pQT25 | A | 116 | 10718 | 1.00% |
|  |  |  |  |  |  |  |
| C413-1-12-37 | BSMV-pQT17, 18, 23, 25 and 26 mix | pQT26 | A | 761 | 4252 | 17.90% |
| C413-1-12-34 | BSMV-pQT17, 18, 23, 25 and 26 mix | pQT26 | A | 6 | 7709 | 0.08% |
| C413-1-12-40 | BSMV-pQT17, 18, 23, 25 and 26 mix | pQT26 | A | 227 | 5555 | 4.09% |
| C413-1-12-35 | BSMV-pQT17, 18, 23, 25 and 26 mix | pQT26 | A | 132 | 7164 | 1.84% |
| C413-1-12-28 | BSMV-pQT17, 18, 23, 25 and 26 mix | pQT26 | A | 213 | 6789 | 3.14% |
| C413-1-12-36 | BSMV-pQT17, 18, 23, 25 and 26 mix | pQT26 | A | 108 | 6175 | 1.75% |
| C413-1-12-29 | BSMV-pQT17, 18, 23, 25 and 26 mix | pQT26 | A | 270 | 2758 | 9.79% |
| C413-1-12-30 | BSMV-pQT17, 18, 23, 25 and 26 mix | pQT26 | A | 207 | 5812 | 3.56% |
| C413-1-12-31 | BSMV-pQT17, 18, 23, 25 and 26 mix | pQT26 | A | 129 | 6862 | 1.88% |
| C413-1-12-32 | BSMV-pQT17, 18, 23, 25 and 26 mix | pQT26 | A | 744 | 9235 | 8.06% |
| C413-1-12-88 | BSMV-MTLT5 | pQT26 | A | 15 | 4896 | 0.31% |

**Table S7.** Proportion of NGS reads carrying 71-bp deletion.

| **M0_Plant_ID** | **Reads with 71 bp deletion** | **total reads** | **Ratio** |
| --- | --- | --- | --- |
| C413-1-12-28 | 215 | 1947 | 11.04% |
| C413-1-12-29 | 554 | 2785 | 19.89% |
| C413-1-12-30 | 265 | 2579 | 10.28% |
| C413-1-12-31 | 393 | 2910 | 13.51% |
| C413-1-12-32 | 393 | 2580 | 15.23% |
| C413-1-12-34 | 205 | 2572 | 7.97% |
| C413-1-12-35 | 179 | 1634 | 10.95% |
| C413-1-12-36 | 321 | 2289 | 14.02% |
| C413-1-12-37 | 147 | 2841 | 5.17% |
| C413-1-12-40 | 279 | 2767 | 10.08% |

**Table S8.** Somatic editing efficiency of targets in the background of other cultivars.

| **Plant ID** | **Inoculation** | **Sequenced target** | **Genome** | **Mutated Reads** | **Total Reads** | **Mutated Percentage** | **Mean** |
| --- | --- | --- | --- | --- | --- | --- | --- |
| 3613474/707-1BC2F1-8-38-15 | BSMV-QT1 | QT1 | A | 120 | 124 | 96.77% | 98.00% |
|  |  | QT1 | B | 224 | 226 | 99.12% |  |
|  |  | QT1 | D | 196 | 201 | 97.51% |  |
| 3613474/707-1BC2F1-8-38-29 | BSMV-QT1 | QT1 | A | 70 | 72 | 97.22% | 94.98% |
|  |  | QT1 | B | 54 | 58 | 93.10% |  |
|  |  | QT1 | D | 103 | 109 | 94.50% |  |
| 3613474/707-1BC2F1-8-38-31 | BSMV-QT1 | QT1 | A | 311 | 316 | 98.42% | 98.73% |
|  |  | QT1 | B | 342 | 345 | 99.13% |  |
|  |  | QT1 | D | 515 | 522 | 98.66% |  |
| 3613474/707-1BC2F1-8-38-23 | BSMV-QT1 | QT1 | A | 92 | 100 | 92.00% | 95.00% |
|  |  | QT1 | B | 138 | 144 | 95.83% |  |
|  |  | QT1 | D | 150 | 156 | 96.15% |  |
| 3613474/707-1BC2F1-8-38-5 | BSMV-GW7T6 | GW7T6 | A | 198 | 352 | 56.25% | 64.39% |
|  |  | GW7T6 | B | 268 | 393 | 68.19% |  |
|  |  | GW7T6 | D | 252 | 370 | 68.11% |  |
| 3613474/707-1BC2F1-8-38-6 | BSMV-GW7T6 | GW7T6 | A | 202 | 699 | 28.90% | 34.01% |
|  |  | GW7T6 | B | 256 | 670 | 38.21% |  |
|  |  | GW7T6 | D | 215 | 610 | 35.25% |  |
| 3613474/707-1BC2F1-8-38-20 | BSMV-GW7T6 | GW7T6 | A | 235 | 507 | 46.35% | 55.41% |
|  |  | GW7T6 | B | 306 | 473 | 64.69% |  |
|  |  | GW7T6 | D | 227 | 406 | 55.91% |  |
| 3613474/707-1BC2F1-8-38-4 | BSMV-GW7T6 | GW7T6 | A | 194 | 263 | 73.76% | 83.73% |
|  |  | GW7T6 | B | 290 | 313 | 92.65% |  |
|  |  | GW7T6 | D | 247 | 297 | 83.16% |  |
| 3613474/707-1BC2F1-8-38-22 | BSMV-GW7T6 | GW7T6 | A | 136 | 209 | 65.07% | 67.77% |
|  |  | GW7T6 | B | 184 | 252 | 73.02% |  |
|  |  | GW7T6 | D | 151 | 234 | 64.53% |  |
| 3613474/707-1BC2F1-8-38-27 | BSMV-GW7T6 | GW7T6 | A | 185 | 475 | 38.95% | 43.84% |
|  |  | GW7T6 | B | 261 | 565 | 46.19% |  |
|  |  | GW7T6 | D | 223 | 486 | 45.88% |  |
| 3613474/707-1BC2F1-8-38-24 | BSMV-GW7T6 | GW7T6 | A | 289 | 400 | 72.25% | 71.41% |
|  |  | GW7T6 | B | 305 | 469 | 65.03% |  |
|  |  | GW7T6 | D | 310 | 397 | 78.09% |  |
| 3613474/707-1BC2F1-8-38-7 | BSMV-GW7T6 | GW7T6 | A | 155 | 578 | 26.82% | 34.78% |
|  |  | GW7T6 | B | 241 | 553 | 43.58% |  |
|  |  | GW7T6 | D | 190 | 554 | 34.30% |  |
| 3613474/707-1BC2F1-8-38-25 | BSMV-GW7T6 | GW7T6 | A | 360 | 512 | 70.31% | 78.36% |
|  |  | GW7T6 | B | 453 | 516 | 87.79% |  |
|  |  | GW7T6 | D | 389 | 506 | 76.88% |  |
| 3613474/707-1BC2F1-8-38-16 | BSMV-GW7T6 | GW7T6 | A | 265 | 549 | 48.27% | 56.29% |
|  |  | GW7T6 | B | 418 | 617 | 67.75% |  |
|  |  | GW7T6 | D | 297 | 575 | 51.65% |  |
| 3613474/707-1BC2F1-8-38-23 | BSMV-QT1 | GW7T6  GW7T6  GW7T6 | A | 0 | 434 | 0.00% | 0.00% |
|  |  |  | B | 0 | 484 | 0.00% |  |
|  |  |  | D | 0 | 396 | 0.00% |  |
| KSc44-8-3 | BSMV-GW7T6 | GW7T6 | A | 208 | 392 | 53.06% | 70.62% |
|  |  | GW7T6 | B | 377 | 477 | 79.04% |  |
|  |  | GW7T6 | D | 333 | 431 | 77.26% |  |
| KSc44-8-4 | BSMV-GW7T6 | GW7T6 | A | 87 | 294 | 29.59% | 41.45% |
|  |  | GW7T6 | B | 132 | 250 | 52.80% |  |
|  |  | GW7T6 | D | 113 | 257 | 43.97% |  |
| KSc44-8-6 | BSMV-GW7T6 | GW7T6 | A | 350 | 542 | 64.58% | 78.46% |
|  |  | GW7T6 | B | 529 | 579 | 91.36% |  |
|  |  | GW7T6 | D | 436 | 555 | 78.56% |  |
| KSc44-8-5 | BSMV-PDS | GW7T6  GW7T6  GW7T6 | A | 0 | 410 | 0.00% | 0.09% |
|  |  |  | B | 1 | 345 | 0.29% |  |
|  |  |  | D | 0 | 354 | 0.00% |  |
